# An improved large signal model of full-bridge LLC converter

**DOI:** 10.1371/journal.pone.0205904

**Published:** 2018-10-19

**Authors:** Lele Yao, Donghui Li, Lingling Liu

**Affiliations:** School of Electrical and Information Engineering, Tianjin University, Tianjin, China; University of Science and Technology Beijing, CHINA

## Abstract

For the full-bridge LLC converter, it is necessary to establish a large signal model with low-complexity and high-precision when the current of filter inductor works under the continuous conduction mode (CCM) situation. In terms of low complexity, peak values of resonant state variables and average values of slow state variables are taken as the measured indexes between models and actual converter. Models based on the first harmonic approximation (FHA) are studied in depth because they have lower complexity than the existing models. From prospective of high precision, deficiencies of typical FHA model are explained in the light of harmonic generation mechanism of primary current and influence of Fourier series on the typical equivalent circuit. Therefore, transient process of each metallic oxide semiconductor field effect transistor (MOSFET) and its related fast recovery diode, transient process of Schottky rectifier diodes, piecewise equivalent circuit, appropriate selection of variables, and simplified modified dynamic equations are all considered. Furthermore, the unified proposed model is achieved according to the equivalent principle of indexes between aforementioned improved analyses and proposed model. Numerical results of typical FHA model and proposed model are given in accordance with the key indexes. Then, corresponding experimental results are also presented. Differences of indexes between the two models and the actual converter are compared respectively. Though complexity of proposed model is the same as that of typical FHA model, precision of proposed model is higher than that of typical FHA model.

## 1 Introduction

With the popularity of renewable energy generation, kinds of DC-DC converters play important roles in the power conversion system. In the photovoltaic (PV) system, DC-DC converter is connected to PV array and inverter can be taken as load. A large number of advanced maximum power point tracking (MPPT) algorithms have been successfully implemented in the DC-DC converter. These algorithms mainly include two-step algorithm for global MPPT [1], natural cubic-spline-guided Jaya algorithm (S-Jaya) [2], compensation power DC-DC converter for the distributed model-based MPPT (CPDC-DMBMPPT) [3], and model predictive-based controller (MPC) with a fixed step that is combined with the traditional incremental conductance (INC) algorithm [4]. In the electric vehicle (EV) system, DC-DC converter is intermediate link between different energy storages and DC bus. For example, in [5], high voltage battery-supercapacitor and DC motor are connected to the two ports of a kind of typical bidirectional DC-DC converter respectively. Proposed power-split strategy is developed to track real-time load profiles and determine cutoff frequency. In [6], dual battery energy source and DC-bus of different voltage levels are connected to the interfaces of proposed bidirectional DC-DC converter respectively. Furthermore, different modes of power transfer can be effectively carried out according to the hybrid model. In the modern electric ship medium voltage DC power system such as [7], DC-DC modular multilevel converter with a medium-frequency transformer is the promising transmission device. For the complicated converter, a novel fundamental period averaging (FPA) method is applied to obtain the steady states and dynamics. In the mining applications such as [8], DC-DC converter is necessary in the mobile mining equipment. In [8], the converter employs a passive LCL filter instead of high-frequency transformer so that core loss of transformer can be eliminated. Meanwhile, the proposed control strategy based on single phase *pq* theory is developed to ensure stable and robust operation.

Isolated full-bridge DC-DC converter is widely used in the aforementioned fields when electromagnetic isolation is required. Mastering the properties of full-bridge converter help researchers establish the appropriate mathematical model which mainly includes small signal model and large signal model. Furthermore, this kind of DC-DC converter can be effectively improved and designed. Small signal model can be the transfer functions of duty cycle to filter current, filter current to output voltage, duty cycle to output voltage, and input voltage to output voltage respectively [9–10]. All the transfer functions are formulated according to respective small signal equivalent circuit which decides characteristics of DC operating points and their neighborhood operating points. However, analysis on the characteristics of operating points is no longer appropriate when input voltage and load occur to a wide range of changes in the certain region. In this case, large signal model need to be founded and models in the following text are referred to it. Large signal model can be state equations on the average current of primary inductor and average voltage of parallel capacitor [11], the average output voltage and current [12], the average voltage of filter capacitor, average input current and output current [13], and so on. All the state equations are also derived from respective equivalent circuit which can reflect the properties of converter under the certain region. According to steady state and transient state of converter, large signal model can be obtained when input voltage and load work at the specific region.

Full-bridge LLC converter is one kind of isolated full-bridge DC-DC converters. Similarly, it is necessary to recognize the analyses bases related to steady state and transient state of converter before modeling and further design. In [14], proposed automatic resonant frequency tracking (ARFT) method is based on the model which contains phase and gain relationship of an exclusive variable pair in the LLC network. In addition, realistic ramp variation in resonant frequency is also reflected in the model. In [15], synchronous rectifier turn-on time is extended to improve control capability under light load condition. Investigated model cannot live without equivalent circuit which is made fully use of so that proposed Fourier series analysis can be developed. In [16], Lagrangian model is established according to the energy equivalency and equivalent circuit which includes magnetic circuit of proposed integrated transformer. State variables comply with the flux cancellation concept and operation rules of equivalent magnetic components. In summary, it is crucial to have knowledge of analyses on the steady state and transient state before establishing large signal model.

Analysis bases on the steady state properties of LLC converter are formulated in detail as follows. In [17–19], six operation modes are illustrated and they are classified by the relationship between switching frequency, resonant frequency, and conduction mode of output current. Equivalent circuits are normalized according to the FHA. Switching loss, conduction loss of switch devices, copper loss and core loss of inductive components are all considered in [18]. However, influence of transient processes related to switch devices on the resonance variables is neglected. In [20], the steady state characteristics are divided into three regions based on the FHA and corresponding equivalent circuit. Voltage conversion ratio, resonance frequency, characteristic impedance, quality factor, inductance ratio, normalized frequency, and equivalent load are all given. However, these relationships are obtained by the assumption that resonant network has well selectivity of sinusoidal signals. In [21] and [22], secondary leakage inductance is contained in the equivalent circuit. Developed analyses and equations are established according to the modified equivalent circuit. However, the equivalent converted load at primary side still comes from the typical FHA, which influences the precision accuracy of proposed circuit. In [23], equivalent isolated transformer circuit is improved by using the coupling coefficient to express inductances and turns ratio. Voltage gain and switching frequency can be predicted with high accuracy but equivalent load is still derived from the FHA. In addition, the transient processes of switch devices are not considered. In [24], time domain analysis is introduced to improve the accuracy of voltage gain. Relationship between voltage gain, duty cycle, quality factor, and inductor ratio can be obtained by solving the numerical equations. However, Definition of quality factor still comes from the FHA. In [25], denominator of voltage gain is divided into resonant factor and load factor. They are related to the inductance ratio and quality factor respectively. The modified gain is obtained by linearizing and averaging proposed equivalent circuit and its theoretical curves. However, the transient characteristics of switches are neglected and it will cause deviations. In [26], proposed LLC converter is equivalent by applying the FHA. Input source and load are also derived from the FHA. Secondary leakage inductance is also neglected. However, it is necessary that the LLC network has well capacity of selecting fundamental wave so that boundary conduction mode can be strongly applied. In [27], frequency modulation and duty ratio modulation are synchronously implemented. Primary current and voltage of resonant capacitor are not sinusoidal because of the asymmetrical hybrid modulation. However, equivalent single output circuit is obtained by the typical FHA.

From what has been analyzed above, the following drawbacks can be concluded. (1) Dead time of the two MOSFETs at same arm of bridge is neglected. Furthermore, the analysis on the primary current is not exactly because influence of transient process is not considered. (2) Commutation process of two Schottky rectifier diodes is also neglected. It is important to take the process into account when filter inductor works under the CCM condition because not only output current but also primary current is affected by the process. (3) Resonant variables such as primary current, current of magnetizing inductance, and voltage of resonant capacitor are non-sinusoidal due to the transient processes of MOSFETs and Schottky rectifier diodes. Equivalent converted load at primary side is derived from the FHA and it needs to be modified under the CCM condition.

Analysis bases on the transient state properties of LLC converter are discussed in detail as follows. In [28], differential equations deduced from the corresponding equivalent circuit are transformed according to the typical averaging method. Averaged variables are investigated when load is sudden to change. Their trends of change are similar to the corresponding peak variables but not completely coincident. In [29], oscillation frequency and amplitude of output current are obtained by the FHA and extended describing functions (EDF) respectively. Method of transient current ripple reduction can be directly designed by the FHA and numerical solutions can be given by the EDF. In [30], feedback linearization is based on two-order dynamic equations derived from the equivalent FHA circuit. Characteristics of FHA are fully used of so that transient states can be further simplified. In [31], eigenvalues displacement for different operating conditions defined by switching frequency and displacement of dominant poles for different load are analyzed by equivalent resonant circuit deduced from the FHA. However, aforementioned analyses can be used when the LLC network has well sinusoidal. In [32], segmented equivalent circuits are obtained according to the different operation modes. Furthermore, state-plane composed of voltage of resonant capacitor and primary current is analyzed so that the optimal trajectory can be found during load transients. In [33], the main analysis method is similar to [32]. Voltage and average current of filter capacitor make up the two dimensional state-plane. In [34], normalized output voltage, primary current, and voltage of resonant capacitor are shown in the three dimensional state-plane. Furthermore, startup dynamic performance of voltage and average current of filter capacitor are analyzed in the time domain by the two dimensional state-plane. However, aforementioned variation tendencies of other unemployed variables are not clear due to the simplified state-planes in [32–34]. In order to get other something important, complex analytic variables need to be taken full use of. In [35] and [36], seventh-order state equations which contain real and imaginary parts are used. Average absolute primary current and output voltage are the output variables of converter. These dynamic equations are derived from the equivalent circuit composed of sine and cosine components. When the parameters suddenly change, transient process can be verified. However, it is benefit when the complexity of state variables is reduced. In [37], resonant variables and output voltage can be predicted by numerical equations during the startup process. Furthermore, the relationships between startup current, initial startup frequency, and duty cycle are presented. However, it is complicated that twelve modes need to be decomposed for the combination of different state variables.

From what has been discussed above, the following conclusions can be drawn. (1) Variables which are used to analyze transient state properties of converter need to be appropriate. A few numbers of variables result in the lack of something important and transient states are generally reflected by the approximate solutions of differential equations. On the contrary, a large numbers of variables lead to the high complexity of state equations. It is inconvenient to further design the converter by transient process. (2) Only the LLC network has well sinusoidal selectivity, can analytic equations based on the FHA be effectively used. If resonant variables are much more different from sinusoidal signal, the typical FHA cannot be applied directly and they need to be modified. Routine process of correction are much complex because large number of series are used.

In this paper, the condition that current of filter inductor works under CCM is investigated. In order to found a large signal model with low-complexity and high-precision, aforementioned five existing shortcomings have to be paid attention to. Firstly, three existing typical models are presented and respective complexities are considered according to the size of each table in the digital signal processor (DSP). Peak values of resonant state variables and average values of slow state variables are taken as the indexes between models and actual converter. Models based on the FHA are researched in the following text because they have the same lowest complexity. Secondly, equivalent circuit is obtained by the FHA. Differences between equivalent circuit and LLC converter are input source, simplified models of components, and equivalent load. Meanwhile, analyses on the steady state and transient state of model are given. Large signal model is established according to the aforementioned analyses. Variables of the nonlinear mathematical model are the instantaneous values of resonant variables and the average values of slow variables. Thirdly, reasons for the low accuracy of FHA under the studied condition are explained. There are two main viewpoints to formulate the reasons. They are harmonic generation mechanism of primary current and influence of Fourier series on the equivalent circuit respectively. In the meantime, work region of converter is given. Furthermore, combined with the five drawbacks mentioned above, improved equivalent circuit and corresponding improved analyses on the steady state and transient state are shown. Proposed mathematical model is established based on the equivalent principle of indexes. Correction coefficients need to be solved so that proposed model is certain. Lastly but very important, numerical operations and experiments need to be done. For the typical FHA model and the proposed model, steady state and transient state of mentioned indexes are calculated by Matlab. Correspondingly, results of actual converter are presented. What is more, accuracy of proposed model is verified by comparing differences of indexes between two models and actual converter.

## 2 Existing models and complexity

Abbreviations in this section are defined in Table 1.

**Table 1:** Nomenclature.

Symbol	Definition	Symbol	Definition
**U**_**in**_	DC input source	***u***_***Ca***_	Instantaneous voltage of C_a_
**Q1-Q4**	MOSFETs with fast recovery diodes	***i***_***Lp***_	Instantaneous current of L_P_
**C**_**a**_	Resonant capacitor	***i***_***Lm***_	Instantaneous current of L_M_
**L**_**P**_	Leakage inductance	***i***_***Lf***_	Instantaneous current of L_f_
**L**_**M**_	Excitation inductance	***u***_***C***_	Instantaneous voltage of C
**D1-D2**	Schottky rectifier diodes	***u***_***AB***_	Voltage between A and B
**L**_**f**_	Filter inductor	**ω**	Switching angular frequency
**C**	Filter capacitor	**C**_**DS**_	Drain-source capacitance of MOSFET
**R**	Load of converter		

When analysis bases related to steady state and transient state of converter are done, establishing large signal model is natural step. There are several modeling methods to be referenced. In [38], prediction model with improved extreme learning machine (ELM) is proposed. Prediction accuracy is enhanced by considering the distribution of data through the use of *L*_2_ norm. In [39], fine-grained activity recognition model is achieved by using improved ELM. The kernel risk-sensitive loss (KRSL) is incorporated into a novel multilayer neural network and identification accuracy can be further enhanced. In [40], generalized frequency-dependent averaged model is introduced by designing suitable jump mode which can approximate the transient states during switching time. The model can be treated as differential algebraic equations. In [41], approximate discrete-time model of nonisolated DC-DC converters is proposed and low-pass properties of converters are taken into account. Proposed model can capture the natural sampling characteristics and dimension of system matrices can be reduced. In [42], for the three-phase dual active bridge (3p-DAB) converter, generalized state space averaging (GSSA) model based on the dynamic phasor is developed. Proposed model is combined with state space averaging (SSA) method and precise for the stability analysis can be enhanced. In [43], generalized average models of dual active bridge (DAB) converters are proposed by using a triple phase shift modulation which includes single, dual and extended schemes. Modeling framework is a tradeoff between complexity and accuracy. Methods mentioned above have guiding significance for modeling. However, for the LLC converter, there are three typical models in the existing literatures and they are listed in detail as follows.

**Fig 1:**
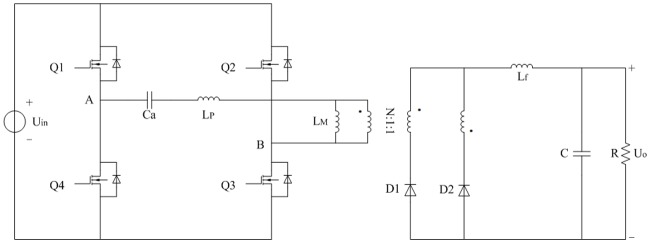
Topology of full-bridge LLC converter. This is the main circuit.

The studied full-bridge LLC converter is presented in Fig 1. Symbols in the figure are described in the nomenclature. In addition, the turn number ratio of primary side and secondary sides is N:1:1.

### Model 1

Typical model 1 is originated from the [44]. There are three steps to establish the model. Firstly, approximate expressions of resonant variables are presented. They are usually the combinations of sine function and cosine function. Secondly, nonlinear terms of state equations are approximated by the extended describing function based on the time domain and frequency domain analysis. Lastly, harmonic balance method is applied so that the sine and cosine part are separated. Then, the final simplified large signal model is obtained.

#### A First step: Approximate expressions of resonant variables

$$u_{Ca}=u_{1}\text{sin}\omega t-u_{2}\text{cos}\omega t$$

$$i_{Lp}=i_{1}\text{sin}\omega t-i_{2}\text{cos}\omega t$$

$$i_{Lm}=i_{3}\text{sin}\omega t-i_{4}\text{cos}\omega t$$

$${\dot{u}}_{Ca}=({\dot{u}}_{1}+\omega u_{2})\text{sin}\omega t-({\dot{u}}_{2}-\omega u_{1})\text{cos}\omega t$$

$${\dot{i}}_{Lp}=({\dot{i}}_{1}+\omega i_{2})\text{sin}\omega t-({\dot{i}}_{2}-\omega i_{1})\text{cos}\omega t$$

$${\dot{i}}_{Lm}=({\dot{i}}_{3}+\omega i_{4})\text{sin}\omega t-({\dot{i}}_{4}-\omega i_{3})\text{cos}\omega t$$

#### B Second step: Extended describing function

$$i_{P}=\sqrt{{\left(i_{1}-i_{3}\right)}^{2}+{\left(i_{2}-i_{4}\right)}^{2}}$$

$$u_{AB}=\frac{4}{\pi }U_{in}\text{sin}\omega t$$

$$sign(i_{Lp}-i_{Lm})(u_{Lf}+u_{C})=\frac{4}{\pi }\frac{i_{1}-i_{3}}{i_{P}}(u_{Lf}+u_{C})\text{sin}\omega t-\frac{4}{\pi }\frac{i_{2}-i_{4}}{i_{P}}(u_{Lf}+u_{C})\text{cos}\omega t$$

$$\left|i_{Lp}-i_{Lm}\right|=\frac{2}{\pi }i_{P}$$

#### C Third step: Harmonic balance method

$${\dot{i}}_{1}=\frac{4}{\pi L_{P}}U_{in}-\omega i_{2}-\frac{1}{L_{P}}u_{1}-\frac{4N}{\pi L_{P}}\frac{i_{1}-i_{3}}{i_{P}}(u_{Lf}+u_{C})$$

$${\dot{i}}_{2}=\omega i_{1}-\frac{1}{L_{P}}u_{2}-\frac{4N}{\pi L_{P}}\frac{i_{2}-i_{4}}{i_{P}}(u_{Lf}+u_{C})$$

$${\dot{u}}_{1}=-\omega u_{2}+\frac{1}{C_{a}}i_{1}$$

$${\dot{u}}_{2}=\omega u_{1}+\frac{1}{C_{a}}i_{2}$$

$${\dot{i}}_{3}=-\omega i_{4}+\frac{4N}{\pi L_{P}}\frac{i_{1}-i_{3}}{i_{P}}(u_{Lf}+u_{C})$$

$${\dot{i}}_{4}=\omega i_{3}+\frac{4N}{\pi L_{P}}\frac{i_{2}-i_{4}}{i_{P}}(u_{Lf}+u_{C})$$

$$u_{Lf}=L_{f}\frac{2N[(i_{1}-i_{3})({\dot{i}}_{1}-{\dot{i}}_{3})+(i_{2}-i_{4})({\dot{i}}_{2}-{\dot{i}}_{4})]}{\pi i_{P}}$$

$${\dot{u}}_{C}=\frac{2N}{\pi C}i_{P}-\frac{u_{C}}{R}$$

In conclusion, the final simplified large signal model can be expressed as follows:

$$\dot{X}(t)=F_{1}(X(t),\;U_{in}(t),\;R(t),\;\omega (t))$$ (1)

where *X*(*t*) is composed of *i*_1_, *i*_2_, *i*_3_, *i*_4_, *u*_1_, *u*_2_, and *u*_C_.

### Model 2

Typical model 2 is deduced from the [37]. There are three steps to establish the model. Firstly, different states are analyzed from the view of input port and output port. The unified LC equivalent circuit can be obtained in each state. Furthermore, the equivalent impedance and angular frequency are defined and solutions of the equivalent circuit are given. Secondly, the equivalent parameters contained in the solutions of equivalent circuit under twelve modes are listed in detail. The modes are described as *M*_*n*_ and the number *n* in each mode is fixed. Lastly, conduction modes and transition modes for the switches are defined as *S*_*in*_ respectively. Similarly, the modes for the rectifier diodes are also expressed as *S*_*out*_. Relationships between the *M*_*n*_, *S*_*in*_, and *S*_*out*_ are formulated and it is taken as the mode judger. Meanwhile, the voltage at secondary side of transformer at the current moment and the increment of output voltage can be obtained.

#### A First step

1. **Definition of equivalent impedance and angular frequency**
  $$\{\begin{array}{ll} L_{S}=L_{P}+L_{M}, & C_{S}=\frac{C_{DS}C_{a}}{C_{DS}+C_{a}}\\ Z_{0}=\sqrt{\frac{L_{P}}{C_{a}}}, & \omega_{0}=\frac{1}{\sqrt{L_{P}C_{a}}}\\ Z_{1}=\sqrt{\frac{L_{S}}{C_{a}}}, & \omega_{1}=\frac{1}{\sqrt{L_{S}C_{a}}}\\ Z_{2}=\sqrt{\frac{L_{P}}{C_{S}}}, & \omega_{2}=\frac{1}{\sqrt{L_{P}C_{S}}}\\ Z_{3}=\sqrt{\frac{L_{S}}{C_{S}}}, & \omega_{3}=\frac{1}{\sqrt{L_{S}C_{S}}} \end{array}$$
2. **Solutions of the equivalent circuit**
  $$\left\{ \begin{array}{l} u_{Ce}(n+1)=i_{Le}(n)Z_{e}\text{sin}\omega_{e}t+[u_{Ce}(n)-u_{e}(n)]\text{cos}\omega_{e}t+u_{e}(n)\\ i_{Le}(n+1)=i_{Le}(n)\text{cos}\omega_{e}t+\frac{[u_{Ce}(n)-u_{e}(n)]\text{sin}\omega_{e}t}{Z_{e}} \end{array}\right.$$ (2)

#### B Second step: Twelve modes

M_1_:

$$u_{e}(n)=U_{in}-Nu_{S}(n);L_{e}=L_{P};C_{e}=C_{a};Z_{e}=Z_{0};\omega_{e}=\omega_{0}$$

M_2_:

$$u_{e}(n)=U_{in};L_{e}=L_{S};C_{e}=C_{a};Z_{e}=Z_{1};\omega_{e}=\omega_{1}$$

M_3_:

$$u_{e}(n)=U_{in}+Nu_{S}(n);L_{e}=L_{P};C_{e}=C_{a};Z_{e}=Z_{0};\omega_{e}=\omega_{0}$$

M_4_:

$$u_{e}(n)=-Nu_{S}(n);L_{e}=L_{P};C_{e}=C_{S};Z_{e}=Z_{2};\omega_{e}=\omega_{2}$$

M_5_:

$$u_{e}(n)=0;L_{e}=L_{S};C_{e}=C_{S};Z_{e}=Z_{3};\omega_{e}=\omega_{3}$$

M_6_:

$$u_{e}(n)=Nu_{S}(n);L_{e}=L_{P};C_{e}=C_{S};Z_{e}=Z_{2};\omega_{e}=\omega_{2}$$

M_7_:

$$u_{e}(n)=-U_{in}-Nu_{S}(n);L_{e}=L_{P};C_{e}=C_{a};Z_{e}=Z_{0};\omega_{e}=\omega_{0}$$

M_8_:

$$u_{e}(n)=-U_{in};L_{e}=L_{S};C_{e}=C_{a};Z_{e}=Z_{1};\omega_{e}=\omega_{1}$$

M_9_:

$$u_{e}(n)=-U_{in}+Nu_{S}(n);L_{e}=L_{P};C_{e}=C_{a};Z_{e}=Z_{0};\omega_{e}=\omega_{0}$$

M_10_:

$$u_{e}(n)=-Nu_{S}(n);L_{e}=L_{P};C_{e}=C_{a};Z_{e}=Z_{0};\omega_{e}=\omega_{0}$$

M_11_:

$$u_{e}(n)=0;L_{e}=L_{S};C_{e}=C_{a};Z_{e}=Z_{1};\omega_{e}=\omega_{1}$$

M_12_:

$$u_{e}(n)=Nu_{S}(n);L_{e}=L_{P};C_{e}=C_{a};Z_{e}=Z_{0};\omega_{e}=\omega_{0}$$

#### C Third step

1. **Conduction modes for the switches**
  $$\{\begin{array}{ll} S_{in}=1, & Q1,\;Q3\;ON\\ S_{in}=0, & Q1,\;Q2\;or\;Q3,\;Q4\;ON\\ S_{in}=-1, & Q2,\;Q4\;ON \end{array}$$
2. **Transition modes for the switches**
  $$\{\begin{array}{ll} S_{in}=1, & u_{AB}\geq U_{in}\;and\;i_{Lp}<0\\ S_{in}=-2, & -U_{in}<u_{AB}<U_{in}\\ S_{in}=-1, & u_{AB}\leq -U_{in}\;and\;i_{Lp}>0 \end{array}$$
3. **Modes for the rectifier diodes**
  $$\{\begin{array}{ll} S_{out}=1, & i_{Lp}>i_{Lm}\\ S_{out}=0, & i_{Lp}=i_{Lm}\;and\;\left|u_{Lm}\right|<Nu_{S}(n)\\ S_{out}=-1, & i_{Lp}<i_{Lm} \end{array}$$
4. **Mode judger**
  $$M_{n}=-3S_{in}-S_{out}+5$$
5. **Voltage at secondary side of transformer**
  $$u_{S}(n)=L_{f}NS_{out}\frac{\left[i_{Lp}(n)-i_{Lm}(n)]-[i_{Lp}(n-1)-i_{Lm}(n-1)\right]}{t(M_{n-1})}+u_{C}(n)$$
6. **Increment of output voltage**
  $$u_{C}(n+1)-u_{C}(n)=\frac{NS_{out}C_{a}[u_{Ca}(n+1)-u_{Ca}(n)]}{C}-\frac{u_{C}(n)}{RC}t(M_{n})$$

In conclusion, the (2) are applied again and final simplified large signal model can be expressed as follows:

$$X(n+1)=F_{2}(X(n),\;U_{in}(n),\;R(n),\;\omega [S_{in}(n)])$$ (3)

where *X*(*n*) is composed of *i*_*Le*_(*n*) and *u*_*Ce*_(*n*).

### Model 3

Typical model 3 is derived from the [34]. There are three steps to establish the model. Firstly, the *t*_*k*_ is defined as the instants when the structures of converter change and *S* is defined as the switching function. Secondly, the main differential equation and auxiliary equations during the two adjacent half cycles are formulated. Integral upper limit functions exist in these equations. Lastly, a homogeneous linear differential equation with constant coefficients is obtained by differentiating the integral upper limit functions and applying the elimination method. The new higher order differential equation is unified. Its characteristic equation needs to be solved by combining with piecewise differential equations which occur in the second step. Therefore, other variables can be obtained. All the variables can be described by simplified expressions.

#### A First step: Definition of *t*_*k*_ and *S*

$$t_{k+1}-t_{k}=\frac{\pi }{\omega },\;k\in \{0,\;1,\;2,\;\ldots \}$$

$$\{\begin{array}{ll} S=1, & Q1,\;Q3\;ON\\ S=-1, & Q2,\;Q4\;ON \end{array}$$

#### B Second step

1. **Half cycle *k*th (*t***_***k***_ **< *t* < *t***_***k*+1**_**)**
  Main equation:
  $$-U_{in}+L_{P}{\dot{i}}_{Lp}+\frac{1}{C_{a}}\int_{t_{k}}^{t}i_{Lp}(\tau )d\tau +u_{Ca}(t_{k})+Nu_{S}=0$$
  Auxiliary equations:
  $$Nu_{S}=L_{M}{\dot{i}}_{Lm}$$
  $$Nu_{S}=N\{L_{f}{\dot{i}}_{Lf}+\frac{1}{C}\int_{t_{k}}^{t}[i_{Lf}(\tau )-\frac{u_{C}(t_{k})}{R}]d\tau +u_{C}(t_{k})\}$$
  $$i_{Lp}=i_{Lm}+\frac{i_{Lf}}{N}$$
2. **Half cycle (*k*+1)th (*t***_***k*+1**_ **< *t* < *t***_***k*+2**_**)**
  Main equation:
  $$U_{in}+L_{P}{\dot{i}}_{Lp}+\frac{1}{C_{a}}\int_{t_{k+1}}^{t}i_{Lp}(\tau )d\tau +u_{Ca}(t_{k+1})+Nu_{S}=0$$
  Auxiliary equations:
  $$Nu_{S}=L_{M}{\dot{i}}_{Lm}$$
  $$-Nu_{S}=N\{L_{f}{\dot{i}}_{Lf}+\frac{1}{C}\int_{t_{k+1}}^{t}[i_{Lf}(\tau )-\frac{u_{C}(t_{k+1})}{R}]d\tau +u_{C}(t_{k+1})\}$$
  $$i_{Lp}=i_{Lm}-\frac{i_{Lf}}{N}$$

#### C Third step

1. **Unified differential equation**
  $$(\frac{NL_{P}L_{f}}{L_{M}}+\frac{L_{P}}{N}+NL_{f})\frac{d^{5}i_{Lf}}{dt^{5}}+(\frac{L_{P}}{L_{M}C}+\frac{1}{NC_{a}}+\frac{N}{C}+\frac{NL_{f}}{C_{a}L_{M}})\frac{d^{3}i_{Lf}}{dt^{3}}+\frac{N}{C_{a}CL_{M}}\frac{di_{Lf}}{dt}=0$$
2. **Descriptions of variables**
  $$i_{Lp}(n+1)=f_{31}(u_{Ca}(n),\;u_{C}(n),\;S)$$
  $$i_{Lm}(n+1)=f_{32}(u_{Ca}(n),\;u_{C}(n),\;S)$$
  $$u_{Ca}(n+1)=f_{33}(u_{Ca}(n),\;u_{C}(n),\;S)$$
  $$i_{Lf}(n+1)=f_{34}(u_{Ca}(n),\;u_{C}(n),\;S)$$
  $$u_{C}(n+1)=f_{35}(u_{Ca}(n),\;u_{C}(n),\;S)$$

In conclusion, the final simplified large signal model can be expressed as follows:

$$X(n+1)=F_{3}(u_{Ca}(n),\;u_{C}(n),\;U_{in}(n),\;R(n),\;\omega [S(n)])$$ (4)

where *X*(*n+*1) is composed of *i*_*Lp*_(*n+*1), *i*_*Lm*_(*n+*1), *u*_*Ca*_(*n+*1), *i*_*Lf*_(*n+*1), and *u*_*C*_(*n+*1).

Model is applied in the DSP and look-up table method is widely used. Therefore, complexity of model can be defined as the size of each table in this paper. According to the (1), (3), and (4), it can be concluded that state variables at next moment are determined by state variables, input voltage, load, switching angular frequency corresponding to different modes at present moment no matter what the functions *F* are when the tables are used. State variables, input voltage, and load at present moment are deterministic and optional modes of switching angular frequency at present moment are determined by the number of internal functions. The table means state variables at next moment under the certain state variables, input voltage, and load at present moment. In other words, size of each table is product of the dimension of *X* and the number of internal functions related to *ω*. The value is namely the complexity of corresponding model. Complexity for the different existing models can be seen in Table 2.

**Table 2:** Complexity for different models.

	Model 1	Model 2	Model 3
Dimension of *X*	7	2	5
Number of internal functions related to *ω*	1	3	2
Size of each table	7	6	10

For the LLC converter, resonant state variables are *u*_*Ca*_, *i*_*Lp*_, and *i*_*Lm*_. Slow state variables are *i*_*Lf*_ and *u*_*C*_. Most important indexes between models and actual converter are peak values of resonant state variables and average values of slow state variables because they can reflect basic characteristics of LLC converter and they are direct references of further designing converter. Analyses on the FHA provide concise relationships between indexes and it is convenient to establish models. Variables in the FHA models are instantaneous values of resonant state and average values of slow state, but only the indexes are available in the DSP. Namely, dimension of state variables in the FHA models is five. In addition, mode of switching angular frequency in the FHA models is deterministic because the switching angular frequency is not compound function. So the number of internal functions related to switching angular frequency is one. Size of each table is five and it represents the complexity of FHA models. Given the lowest complexity, models based on the FHA analyses are formulated in detail as follows.

## 3 Typical FHA model

Abbreviations in this section are defined in Table 3 except that those have been appeared in aforementioned sections.

**Table 3:** Nomenclature.

Symbol	Definition	Symbol	Definition
**U**_**AB(eq)**_	Fundamental voltage between A and B	***U***_***o***_	Average output voltage
**R**_**eq(FHA)**_	Equivalent primary load based on the FHA	***U***_***Ca_peak***_	Peak voltage of C_a_
***f***	Switching frequency	***I***_***Lp_peak***_	Peak current of L_P_
***f***_***r***_	Resonance frequency	***I***_***Lm_peak***_	Peak current of L_M_
***Z***	Characteristic impedance	***I***_***Lf_av***_	Average current of L_f_
***Q***	Quality factor	***U***_***C_av***_	Average voltage of C
***l***	Inductance ratio	***U***_***C(eq)***_	Equivalent average voltage
***f***_***n***_	Normalized frequency	${\bar{\text{R}}}_{\text{eq}(\text{FHA})}$	Average value of R_eq(FHA)_
***M***	Voltage conversion ratio		

**Fig 2:**
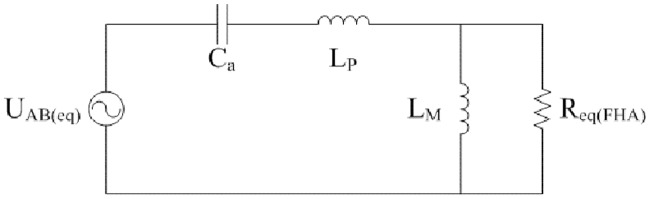
Equivalent circuit derived from FHA. This is the equivalent LLC circuit.

The equivalent circuit of Fig 1 is shown in Fig 2. It is deduced from the typical FHA. L_P_, C_a_, and L_M_ are the same as that in Fig 1. R_eq(FHA)_ is calculated according to the voltage-current characteristic of secondary side of isolated transformer. Equivalent loads R_eq(FHA)_ are different in the analyses on the steady state and transient state.

### 3.1 Analysis on the steady state

According to the [20], U_AB(eq)_, R_eq(FHA)_, *f*, *f*_*r*_, *Z*, *Q*, *l*, *f*_*n*_, *M*, and *U*_*o*_ can be listed as follows:

$$U_{AB(eq)}=\frac{4}{\pi }U_{in}\text{sin}(2\pi ft)$$

$$R_{eq(FHA)}=\frac{8}{\pi^{2}}N^{2}R$$

$$f_{r}=\frac{1}{2\pi \sqrt{L_{P}C_{a}}}$$

$$Z=\sqrt{\frac{L_{P}}{C_{a}}}$$

$$Q=\frac{\pi^{2}}{8}\frac{1}{N^{2}R}Z$$

$$l=\frac{L_{P}}{L_{M}}$$

$$f_{n}=\frac{f}{f_{r}}$$

$$M=\frac{NU_{o}}{U_{in}}=\frac{1}{\sqrt{{\left(1+l-\frac{l}{f_{n}^{2}}\right)}^{2}+Q^{2}{\left(f_{n}-\frac{1}{f_{n}}\right)}^{2}}}.$$

When the converter works under the steady state, Fig 2 is fully utilized. Indexes *U*_*Ca_peak*_, *I*_*Lp_peak*_, *I*_*Lm_peak*_, *I*_*Lf_av*_, and *U*_*C_av*_ are the basic elements of large signal model and the following expressions of them are shown:

$$U_{C\_av}=\frac{U_{in}M}{N}$$ (5)

$$I_{Lf\_av}=\frac{U_{C\_av}}{R}$$ (6)

$$I_{Lm\_peak}=\frac{2U_{C\_av}N}{\pi^{2}L_{M}f}$$ (7)

$$I_{Lp\_peak}=\text{max}[-I_{Lm\_peak}\text{cos}(2\pi ft)+(\frac{\pi I_{Lf\_av}}{2N})\text{sin}(2\pi ft)]$$ (8)

$$U_{Ca\_peak}=\frac{I_{Lp\_peak}}{8C_{a}f}.$$ (9)

### 3.2 Analysis on the transient state

When the input source or the load happens to sudden change, converter works under the transient state. The R_eq(FHA)_ can be expressed as follows:

$$R_{eq(FHA)}=N^{2}[\frac{U_{C(eq)}+L_{f}{\dot{I}}_{Lf}}{C{\dot{U}}_{C(eq)}+\frac{U_{C(eq)}}{\frac{8}{\pi^{2}}R}}].$$ (10)

Imagine that initial time of transient state is *t*_0_ and final time of transient state is *t*_1_. Average value of R_eq(FHA)_ is applied in Fig 2 and it can be indicated in the following expression:

$${\bar{R}}_{eq(FHA)}=\frac{1}{t_{1}-t_{0}}\int_{t_{0}}^{t_{1}}N^{2}[\frac{U_{C(eq)}(\tau )+L_{f}{\dot{I}}_{Lf}(\tau )}{C{\dot{U}}_{C(eq)}(\tau )+\frac{U_{C(eq)}(\tau )}{\frac{8}{\pi^{2}}R}}]d\tau$$ (11)

where the two differential terms are estimated according to the corresponding old and new steady state. Solving process of (11) can be seen in the Appendix.

Furthermore, Fig 2 is fully combined with principle of linear circuit again. Peak values of *u*_*Ca*_, *i*_*Lp*_, and *i*_*Lm*_ under the transient state can be approximately described as the positive envelopes of following state equations:

$$\left\{ \begin{array}{ll} {\dot{u}}_{Ca} & =\frac{1}{C_{a}}i_{Lp}\\ {\dot{i}}_{Lp} & =\frac{1}{L_{P}}\left[-{\bar{R}}_{eq(FHA)}i_{Lp}+{\bar{R}}_{eq(FHA)}i_{Lm}-u_{Ca}+\frac{4}{\pi }U_{in}\text{sin}(2\pi ft)\right]\\ {\dot{i}}_{Lm} & =\frac{{\bar{R}}_{eq(FHA)}}{L_{M}}\left(i_{Lp}-i_{Lm}\right) \end{array}.\right.$$ (12)

where initial values of states are the peak values under the old steady state.

### 3.3 Large signal model

In summary, a unified model needs to be founded so that the steady state and transient state of indexes can be well formulated. Definition of variables mentioned above continues to be used. Following nonlinear mathematical equations are taken as the large signal model based on the typical FHA:

$$C_{a}{\dot{u}}_{Ca}=i_{Lp}$$ (13a)

$$L_{M}{\dot{i}}_{Lm}+L_{P}{\dot{i}}_{Lp}+u_{Ca}=\frac{4}{\pi }U_{in}\text{sin}(2\pi ft)$$ (13b)

$$I_{Lf}=\frac{2}{T}\int_{t}^{t+\frac{T}{2}}\left|N(i_{Lp}-i_{Lm})\right|d\tau$$ (13c)

$$L_{f}{\dot{I}}_{Lf}=\frac{2}{T}\int_{t}^{t+\frac{T}{2}}\frac{\left|L_{M}{\dot{i}}_{Lm}\right|}{N}d\tau -U_{C(eq)}$$ (13d)

$$I_{Lf}=C{\dot{U}}_{C(eq)}+\frac{U_{C(eq)}}{\frac{8}{\pi^{2}}R}$$ (13e)

$$U_{C}=\frac{\pi^{2}}{8}U_{C(eq)}$$ (13f)

where *U*_*C*_ is actual average voltage of C in the theoretical analysis. Differential terms of *I*_*Lf*_ and *U*_*C*(*eq*)_ are zero under the steady state. Solving process of aforementioned model can be seen in the Appendix.

## 4 Insufficiency analysis on the typical FHA

Abbreviations in this section are defined in Table 4 except that those have been appeared in aforementioned sections.

**Table 4:** Nomenclature.

Symbol	Definition	Symbol	Definition
***T***	Switching cycle	***u***_***S***_	Instantaneous voltage of secondary winding
***t***_***on***_	Conduction time of every half cycle	***U***_***S***_	Effective voltage of secondary winding
***u***_***Lp***_	Instantaneous voltage of L_P_	***i***_***S***_	Instantaneous current of secondary winding
***u***_***P***_	Instantaneous voltage of primary winding	***I***_***S***_	Effective current of secondary winding
***i***_***P***_	Instantaneous current of primary winding	***I***_***o***_	Average output current
***t***_***D***_	Transition time of *u*_*AB*_ at half of switching period	**R**_**S(eq)**_	Equivalent load at secondary side of transformer

When the current of filter inductor works under the CCM condition, the typical FHA is not precise to analyze the properties of converter because of the common drawbacks mentioned in the introduction. Furthermore, both harmonic generation mechanism of primary current and influence of Fourier series on the equivalent circuit can explain the reason that why aforementioned deficiencies about typical FHA cannot be neglected. The situation of transient state is similar to that of steady state in every switching period. So the situation of steady state is analyzed in this section.

### 4.1 Harmonic generation mechanism of primary current

In Fig 1, primary current is also the current of L_P_. In fact, voltage between A and B is the square wave and it is described as *u*_*AB*_. The cycle of *u*_*AB*_ is as same as the switching cycle *T*. According to the Fig 2 and definitions in the tables of nomenclature, following equation is easily obtained:

$$i_{Lp}=i_{Lm}+i_{P}.$$ (14)

The *u*_*P*_ and *i*_*P*_ are the quasi square waves which are synchronously changing with *u*_*AB*_ due to the interaction of rectifier diodes, large filter inductor, and filter capacitor. *i*_*P*_ is almost constant in the time *t*_*on*_. Meanwhile, *i*_*Lm*_ monotonously and linearly changes in this time. According to the (14), changing situation of *i*_*Lp*_ is the same as that of *i*_*Lm*_ in the time *t*_*on*_. Furthermore, following equations can be obtained:

$$\frac{du_{Lp}}{dt}=0$$ (15)

$$\frac{du_{Ca}}{dt}=-\frac{du_{P}}{dt}.$$ (16)

It is inferred that *u*_*P*_ and *u*_*Ca*_ both monotonously change in the time *t*_*on*_. In other words, *i*_*Lp*_ is always nonnegative or nonpositive in this time.

Each first half of switching period is defined from moment 0 to moment *T*/2, and each second half of switching period is defined from moment *T*/2 to moment *T*. When the commutation process of rectifier diodes and the nearly constant current of filter inductor are taken into consideration, the load R needs to satisfy the following inequalities so that primary current *i*_*Lp*_ is nonnegative at the moment *T*/2 and *i*_*Lp*_ is nonpositive at the moment *T*:

$$\frac{NU_{o}T}{2L_{M}}\times 2-\frac{NU_{o}T}{2L_{M}}\geq \frac{U_{o}}{NR}$$ (17)

$$\frac{NT}{2L_{M}}\geq \frac{1}{NR}$$ (18)

where the (18) represents the work region of converter.

*u*_*AB*_ and *i*_*Lp*_ at first half of switching period are taken as the example. When the load *R* meets the (18), *i*_*Lp*_ at the moment *T*/2 satisfies the following inequality:

$$i_{Lp}(T/2)\geq \;0.$$ (19)

In addition, following inequality can be achieved according to the monotonicity of *u*_*Ca*_:

$$i_{Lp}(T/2)\leq \;0.$$ (20)

Based on (19) and (20), the relationship of *i*_*Lp*_ between the moment 0 and moment *T*/2 can be listed as follows:

$$i_{Lp}(0)=-i_{Lp}(T/2)=0.$$ (21)

**Fig 3:**
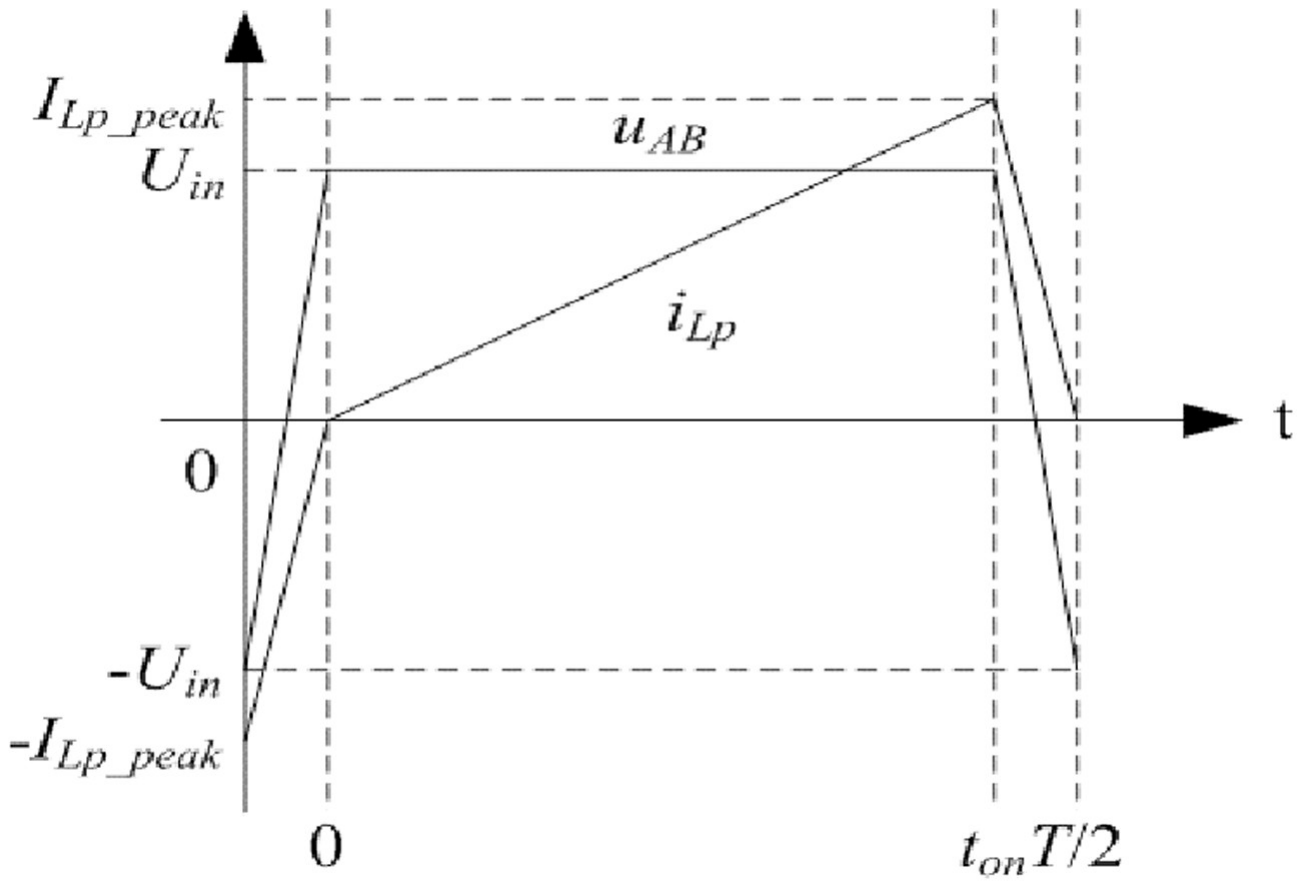
Diagram of *u*_*AB*_ and *i*_*Lp*_ at first half of switching period. This is the illustration of *u*_*AB*_ and *i*_*Lp*_.

From what has been analyzed above, the diagram of *u*_*AB*_ and *i*_*Lp*_ at first half of switching period is shown in Fig 3. Similarly, they are reverse at second half of switching period. It is the reason for harmonic generation in the converter.

### 4.2 Influence of Fourier series on the equivalent circuit

According to the Fig 3, it can be known that *u*_*AB*_ can be taken as square wave. The Fourier series of *u*_*AB*_ is presented as follows:

$$u_{AB}(t)=\frac{4}{\pi }U_{in}\underset{n=1,3,5,\cdots }{\sum }\frac{1}{n}\text{sin}(2\pi nft).$$ (22)

In the light of definition of *t*_*D*_, following equality can be shown:

$$t_{D}=\frac{T}{2}-t_{on}.$$ (23)

What is more, the following inequality can be obtained:

$$u_{AB}(\frac{t_{D}}{2})=U_{in}\gg \frac{4}{\pi }U_{in}\text{sin}(\pi ft_{D}).$$ (24)

The aforementioned relationship (24) indicates that the transition process of *u*_*AB*_ cannot be simply depicted by its fundamental component.

In accordance with Fourier series of square wave and definition of effective value, variables *u*_*S*_, *U*_*S*_, *i*_*S*_, *I*_*S*_, *U*_*o*_, and *I*_*o*_ can be described as follows:

$$u_{S}(t)=\frac{4}{\pi }U_{o}\underset{n=1,3,5,\cdots }{\sum }\frac{1}{n}\text{sin}(2\pi nft-\psi )$$ (25)

$$U_{S}=\frac{2\sqrt{2}}{\pi }U_{o}\sqrt{\underset{n=1,3,5,\cdots }{\sum }\frac{1}{n^{2}}}$$ (26)

$$i_{S}(t)=\sqrt{2}I_{S}^{\prime }\sum_{n=1,3,5,\cdots }\frac{1}{n}\text{sin}(2\pi nft-\psi )$$ (27)

$$I_{S}=I_{S}^{\prime }\sqrt{\underset{n=1,3,5,\cdots }{\sum }\frac{1}{n^{2}}}$$ (28)

$$I_{o}=\frac{2}{T}\int_{0}^{\frac{T}{2}}|i_{S}(t)|dt=\frac{I_{S}}{\sqrt{\sum_{n=1,3,5,\cdots }\frac{1}{n^{2}}}}\frac{2\sqrt{2}}{T}\int_{0}^{\frac{T}{2}}|\underset{n=1,3,5,\cdots }{\sum }\frac{1}{n}\text{sin}(2\pi nft-\psi )|dt=\frac{U_{o}}{R}.$$ (29)

Furthermore, in terms of definition of resistance, R_S(eq)_ can be shown as follows:

$$R_{S(eq)}=\frac{U_{S}}{I_{S}}=\frac{8}{T\pi }R\int_{0}^{\frac{T}{2}}\left|\underset{n=1,2,5,\cdots }{\sum }\frac{1}{n}\text{sin}(2\pi nft-\psi )\right|dt\neq \frac{8}{\pi^{2}}R.$$ (30)

The aforementioned relationship (30) states that R_S(eq)_ cannot be replaced directly by the equivalent load derived from the typical FHA.

According to the (24) and (30), the conclusion can be drawn that Fourier series of variables in the converter need to be applied with several more items when the output of LLC network has not well sinusoidal selectivity. It can be observed from Fig 3 that the primary current is composed of many harmonic components and it cannot be equivalent to the single fundamental component effectively. Therefore, for the studied situation, analyses on the steady state and transient state need to be corrected. Furthermore, the modified large signal model is obtained in the following section.

## 5 Proposed model

Abbreviations in this section are defined in Table 5 except that those have been appeared in aforementioned sections.

**Table 5:** Nomenclature.

Symbol	Definition	Symbol	Definition
**R**_**P**_	Primary AC resistance	***I***_***Lp***_	Average primary current
**R**_**S1**_**-R**_**S2**_	Secondary AC resistances	***u***_**D1**_-***u***_**D2**_	Forward voltage of D1 and D2
**R**_**Lf**_	AC resistance of L_f_	***i***_**D1**_-***i***_**D2**_	Forward current of D1 and D2
**U**_**GS**_	Equivalent pulse driving source	**R**_**loss**_	Equivalent loss resistance
**Ch**	Controlled current source	**R**_**D**_	Equivalent on-resistance of D1 and D2
**C**_**GS**_	Gate-source capacitance	**R**_**eq(pro)**_	Primary load
**C**_**GD**_	Gate-drain capacitance	***T***_***h***_	Half of switching period
**D**	Equivalent ideal diode	**R**_**eq(proposed)**_	Equivalent primary load based on proposed method
**C**_**R**_	Reverse junction capacitance	**R**_**o(eq)**_	Equivalent load of converter
***u***_***DS***_	Instantaneous drain-source voltage	${\bar{\text{R}}}_{\text{eq}(\text{proposed})}$	Average value of R_eq(proposed)_
**C**_**eq**_	Equivalent drain-source capacitance	***k***_**1**_-***k***_**5**_	Correction coefficients

**Fig 4:**
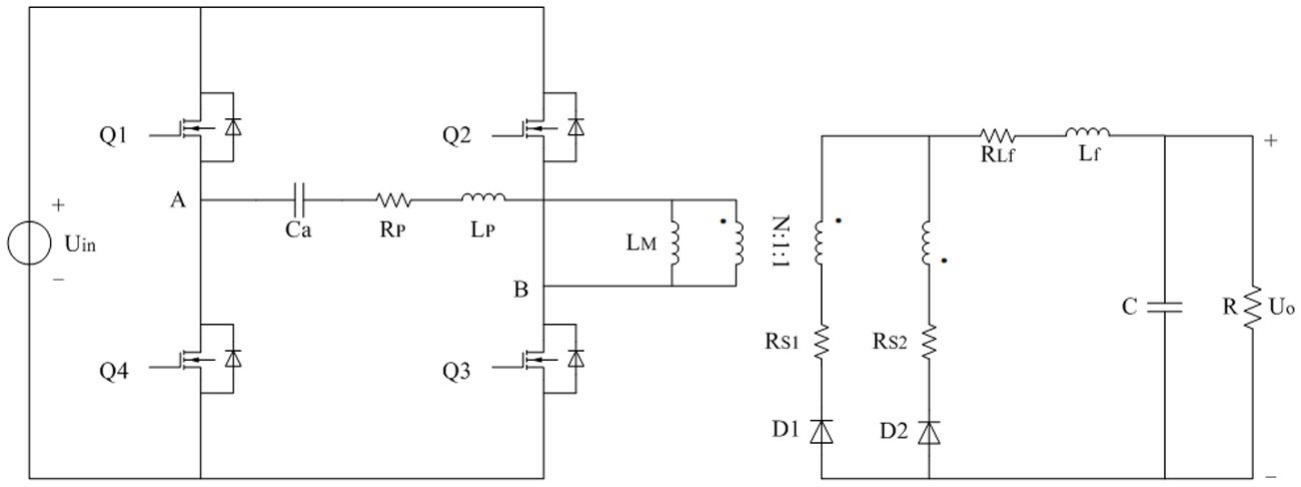
Improved topology of full-bridge LLC converter. This is the improved equivalent main circuit.

The improved topology of full-bridge LLC converter based on the Fig 1 is shown in Fig 4. R_P_ includes primary AC resistance of isolated transformer, equivalent series resistance (ESR) of C_a_, and current sampling resistor. R_S1_ and R_S2_ are the same secondary AC resistances of transformer respectively. R_S_ represents the general term of R_S1_ and R_S2_. R_Lf_ includes AC resistance of L_f_ and current sampling resistor.

When improved equivalent circuit and large signal model are proposed, the insufficiencies mentioned in the introduction are taken into account at the same time. Correspondingly, analyses on the steady state and transient state of converter are formulated in detail as follows.

### 5.1 Analysis on the steady state

#### 5.1.1 Transient process of switches at the arms of full-bridge

**Fig 5:**
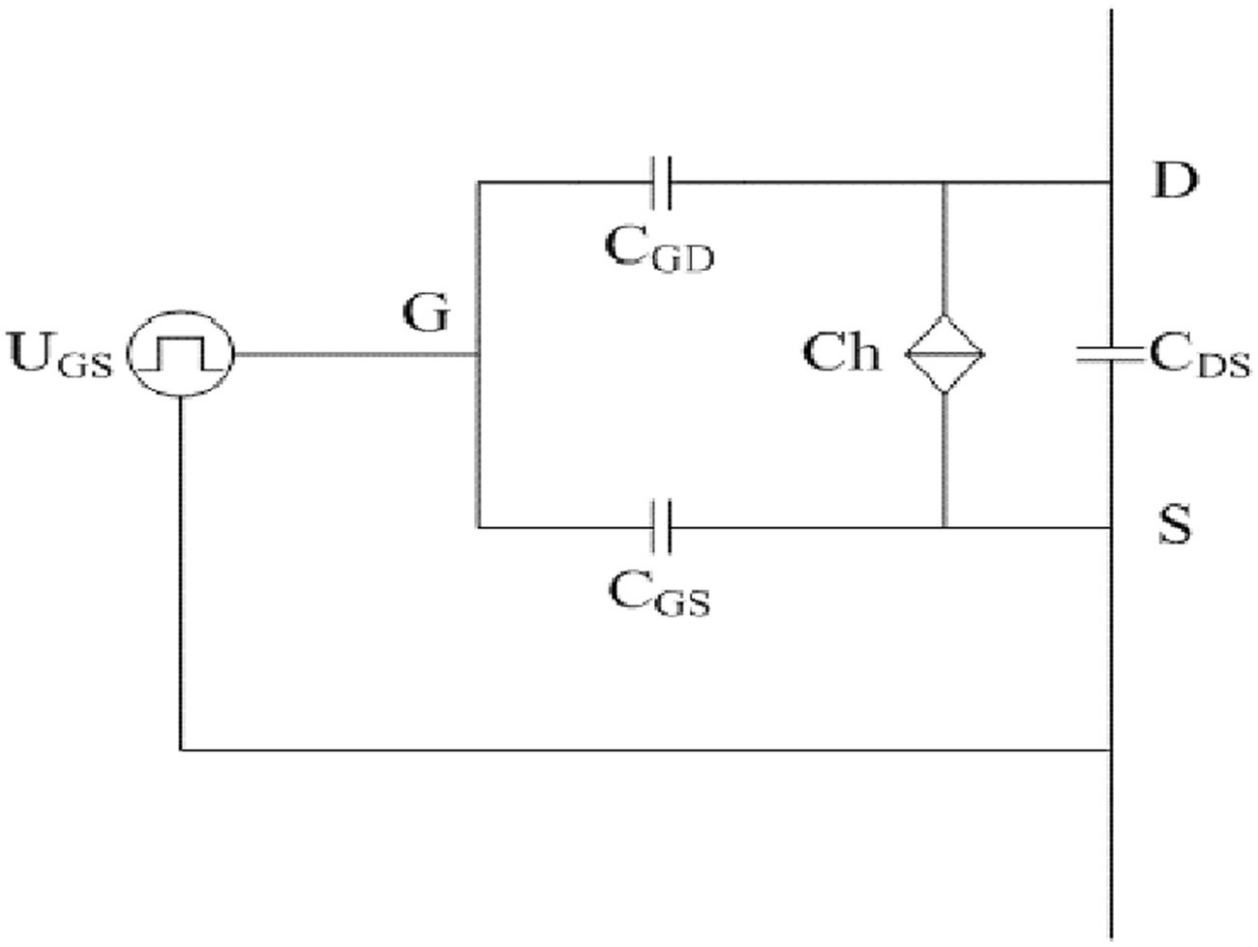
Simplified equivalent model of MOSFET. This is the illustration of MOSFET.

**Fig 6:**
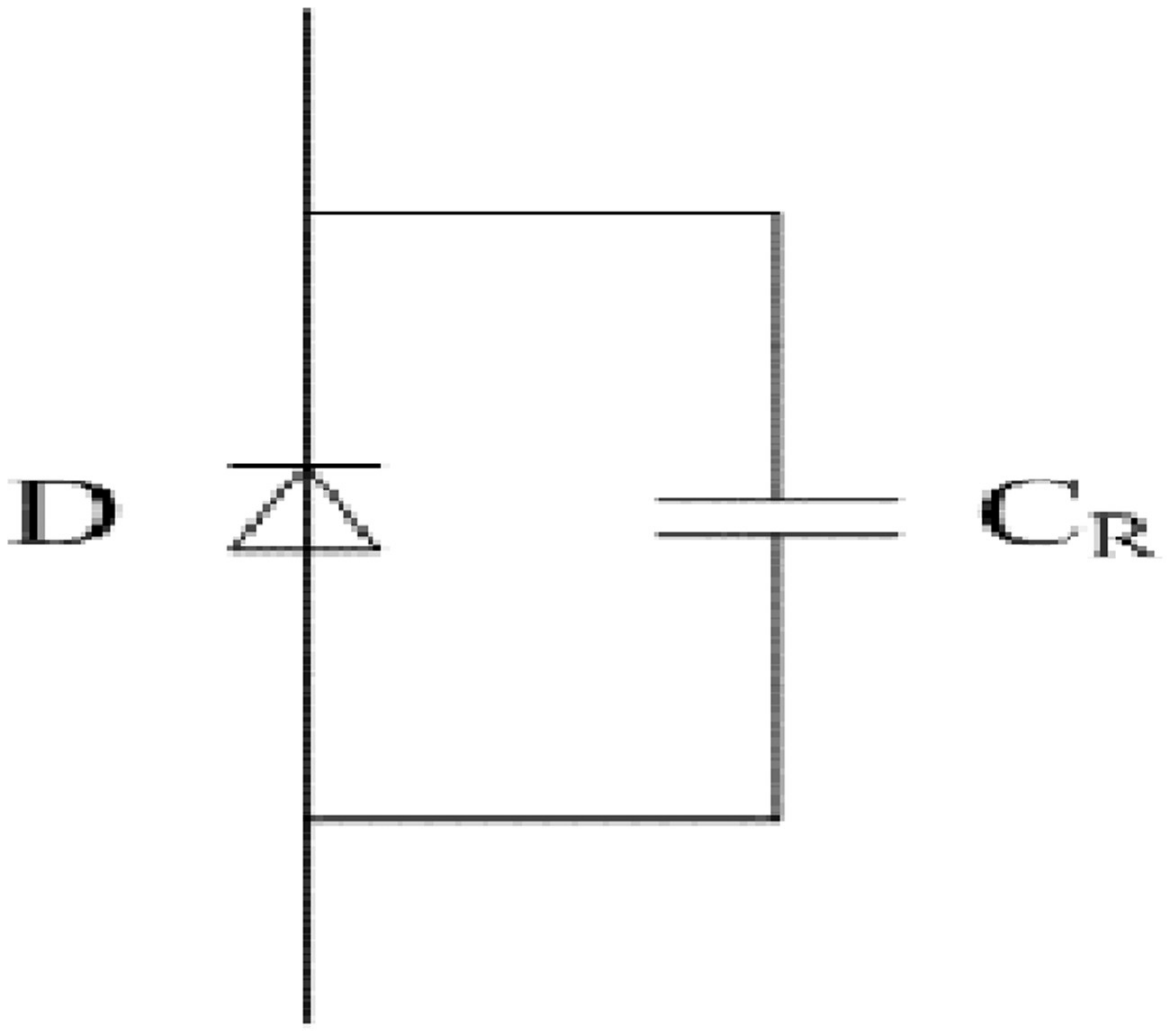
Simplified equivalent model of fast recovery diode. This is the illustration of fast recovery diode.

The switches at the arms of full-bridge are MOSFETs and corresponding fast recovery diodes. Simplified equivalent models of the two main switches are presented in Figs 5 and 6. It can be seen from Fig 5 that the MOSFET is mainly composed of U_GS_, Ch, C_GS_, C_GD_, and C_DS_. Analogously, it can also be observed from Fig 6 that the fast recovery diode is mainly comprised of D and C_R_.

For every MOSFET and corresponding fast recovery diode, the transient process of midpoint voltage U_AB_ is mainly determined by the junction capacitances C_DS_ and C_R_ in each switching period. Both C_DS_ and C_R_ are the nonlinear functions of *u*_*DS*_ and the functions are symbolically shown as follows:

$$C_{DS}=f(u_{DS})$$ (31)

$$C_{R}=g(u_{DS})$$ (32)

where the two functions can be fitted in accordance with the related datasheet.

C_DS_ and C_R_ can fully charge and discharge when work region satisfy the (14). Furthermore, the common equivalent C_eq_ is proposed so that the transient process can be effectively simplified. Based on the *u*_*AB*_ in Fig 3, C_eq_ can be expressed as follows:

$$C_{eq}=\frac{1}{U_{in}}\int_{0}^{U_{in}}\left[f(u_{DS})+g(u_{DS})\right]du_{DS}.$$ (33)

According to the (33), it can be known that C_eq_ is varied with U_in_. The transient process can be formulated by the following equation:

$$I_{Lp}=2C_{eq}\frac{du_{DS}}{dt}.$$ (34)

#### 5.1.2 Transient process of Schottky rectifier diodes

**Fig 7:**
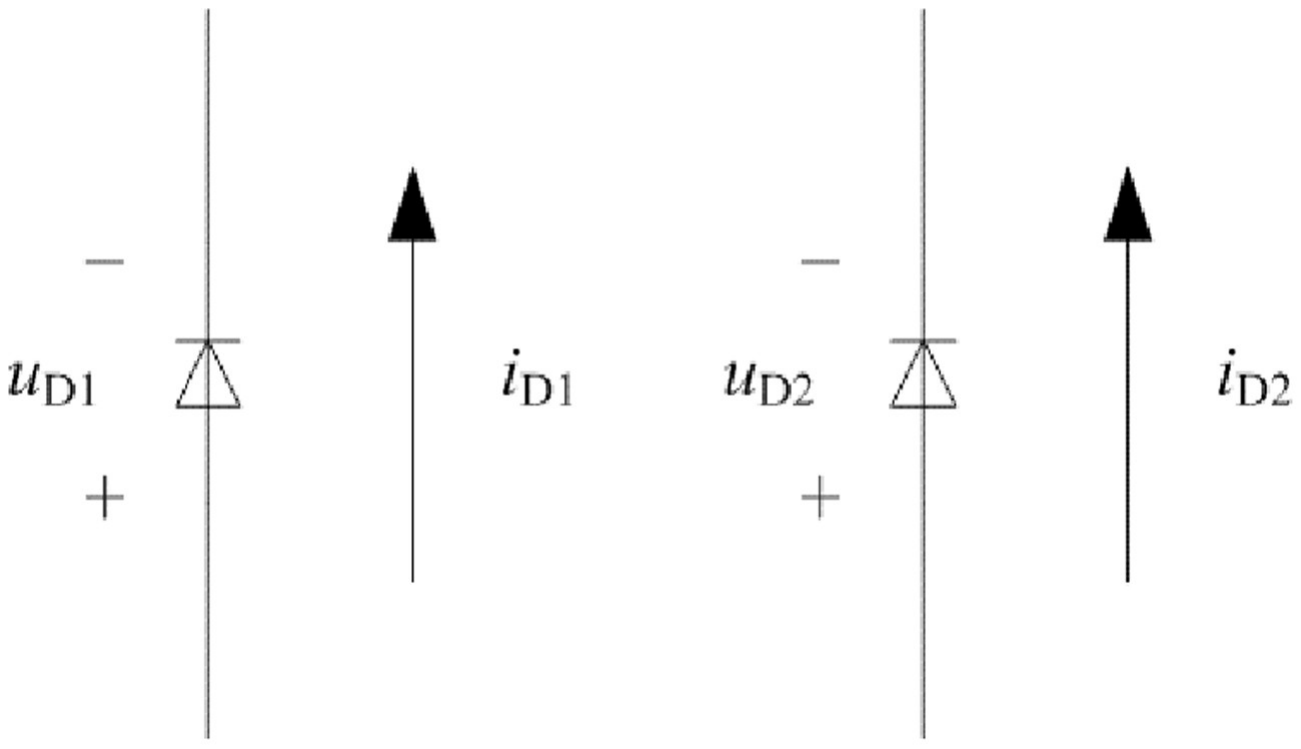
Forward direction of voltage and current of rectifier diodes. This is the diagram of voltage and current.

**Fig 8:**
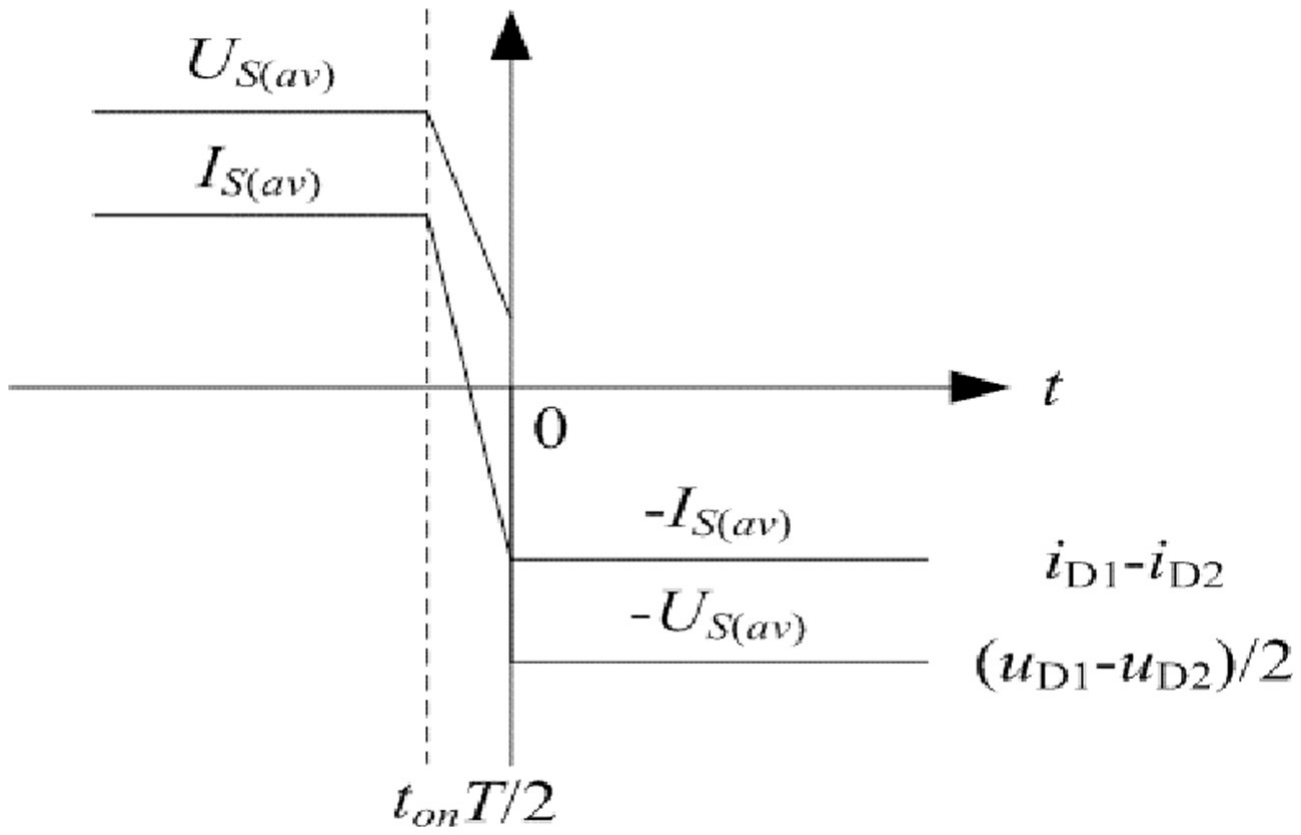
Approximate volt-ampere characteristics of rectifier diodes. This is the relationships between voltage and current.

The Schottky rectifier diodes are D1 and D2 in Fig 4. When the state of MOSFET is switching, it is important to consider the mutual commutation between D1 and D2 under the CCM condition because the current of filter inductor is almost constant. *u*_D1_, *u*_D2_, *i*_D1_, and *i*_D2_ are shown in Fig 7. Approximate current characteristic of D1 and D2 is illustrated in Fig 8.

The *t*_*on*_ and *T*/2 have been defined in the aforementioned section. *U*_*S*(*av*)_ and *I*_*S*(*av*)_ are the average voltage and current of secondary winding from moment 0 to moment *t*_*on*_ respectively. Average voltage and current of secondary winding, average voltage of L_M_ from moment *t*_*on*_ to moment *T*/2 are *U*_*S*(*av*1)_, *I*_*S*(*av*1)_, *U*_*Lm*1_ respectively. Relationships of *U*_*S*(*av*1)_, *I*_*S*(*av*1)_, and *U*_*Lm*1_ are expressed as follows:

$$U_{S(av1)}=\frac{U_{Lm1}}{N}$$ (35)

$$I_{S(av1)}=0.$$ (36)

#### 5.1.3 Piecewise equivalent circuit

Switching loss of MOSFETs, reverse recovery loss, and core loss of transformer objectively exist in the converter. They are varied with the working condition. When the converter operates under the steady state, the corresponding resistance R_loss_ is used to equate the aforementioned loss approximately. R_loss_ is constant under the certain steady state but changes under the different steady states.

According to the Fig 4, it can be known that other conduction loss is caused by the R_P_, R_S_ (R_S1_ = R_S2_), R_Lf_, and R_D_. The R_D_ is equivalent resistance in the forward conduction mode and it can be inferred as follows:

$$u_{D}=ki_{D}+b$$ (37)

$$R_{D}I_{D}=kI_{D}+b$$ (38)

where *k* and *b* are the fitting coefficients according to the forward volt-ampere characteristics. *u*_D_ and *i*_D_ can be seen from the Fig 7. *I*_D_ is the average value of *i*_D_ at the certain time interval.

**Fig 9:**
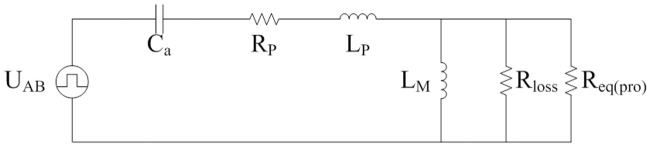
Equivalent circuit from moment 0 to moment *t*_*on*_. This is the equivalent LLC circuit in this time interval.

**Fig 10:**
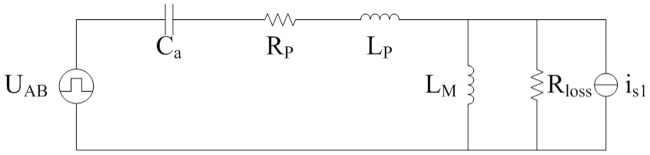
Equivalent circuit from moment *t*_*on*_ to moment *T*/2. This is the equivalent LLC circuit in this time interval.

The first half of switching period from moment 0 to moment *T*/2 is investigated and it is opposite to second half of switching period from moment *T*/2 to moment *T*. According to the Figs 3, 4, 7, 8, and mentioned above, the equivalent circuit from moment 0 to moment *T*/2 is proposed in Figs 9 and 10. In Fig 9, the influence of L_f_ and C is neglected because voltage and current at secondary side are almost constant. Equivalent resistance R_eq(pro)_ can reflect the volt-ampere characteristic of secondary side. In Fig 10, equivalent time-varying current source i_s1_ can be deduced from the Fig 8. The volt-ampere characteristic of secondary side in this time can be shown by the curves in Fig 8.

Characteristic of pulse source U_AB_ can be seen in Fig 3. C_a_, L_P_, R_P_, and L_M_ are the same as that in Fig 4. Average current of i_s1_ is shown in the (36). Based on the definition of resistance, R_eq(pro)_ is expressed as follows:

$$R_{eq(pro)}=N^{2}\frac{U_{S(av)}}{I_{S(av)}}=N^{2}(R_{S}+R_{D}+R_{Lf}+R).$$ (39)

The *T*_*h*_ is used to represent the first half of switching period. The instantaneous voltage of C_a_, instantaneous current of L_P_ and L_M_ at the moment 0 are *u*_*Ca*_(0), *i*_*Lp*_(0), and *i*_*Lm*_(0) respectively. Similarly at the moment *t*_*on*_, they are *u*_*Ca*_(*t*_*on*_), *i*_*Lp*_(*t*_*on*_), and *i*_*Lm*_(*t*_*on*_) respectively. According to the aforementioned analyses and the equivalent circuit shown in Figs 9 and 10, the following algebraic equations are listed by piecewise averaging and linearization:

$${\text{R}}_{eq(proposed)}=\frac{R_{loss}R_{eq(pro)}}{R_{loss}+R_{eq(pro)}}$$ (40a)

$$NL_{M}\frac{i_{Lm}(t_{on})-i_{Lm}(0)}{T_{h}R_{eq(pro)}}R_{D}=kNL_{M}\frac{i_{Lm}(t_{on})-i_{Lm}(0)}{T_{h}R_{eq(pro)}}+b$$ (40b)

$$\frac{i_{Lp}(t_{on})+i_{Lp}(0)}{2}=C_{a}\frac{u_{Ca}(t_{on})-u_{Ca}(0)}{t_{on}}$$ (40c)

$$\frac{i_{Lm}(t_{on})+i_{Lm}(0)}{2}=C_{a}\frac{u_{Ca}(t_{on})-u_{Ca}(0)}{t_{on}}-\frac{L_{M}[i_{Lm}(t_{on})-i_{Lm}(0)]}{t_{on}R_{eq(proposed)}}$$ (40d)

$$L_{P}\frac{i_{Lp}(t_{on})-i_{Lp}(0)}{t_{on}}+L_{M}\frac{i_{Lm}(t_{on})-i_{Lm}(0)}{t_{on}}+\frac{u_{Ca}(t_{on})+u_{Ca}(0)}{2}+\frac{i_{Lp}(t_{on})+i_{Lp}(0)}{2}R_{P}=U_{in}$$ (40e)

$$\frac{-i_{Lp}(0)+i_{Lp}(t_{on})}{2}=C_{a}\frac{-u_{Ca}(0)-u_{Ca}(t_{on})}{T_{h}-t_{on}}$$ (40f)

$$\frac{-i_{Lm}(0)+i_{Lm}(t_{on})}{2}=C_{a}\frac{-u_{Ca}(0)-u_{Ca}(t_{on})}{T_{h}-t_{on}}-\frac{L_{M}[-i_{Lm}(0)-i_{Lm}(t_{on})]}{(T_{h}-t_{on})R_{loss}}-0$$ (40g)

$$L_{P}\frac{-i_{Lp}(0)-i_{Lp}(t_{on})}{T_{h}-t_{on}}+L_{M}\frac{-i_{Lm}(0)-i_{Lm}(t_{on})}{T_{h}-t_{on}}+\frac{-u_{Ca}(0)+u_{Ca}(t_{on})}{2}+\frac{-i_{Lp}(0)+i_{Lp}(t_{on})}{2}R_{P}=0$$ (40h)

$$2C_{eq}\frac{U_{in}}{T_{h}-t_{on}}=\frac{i_{Lp}(t_{on})-i_{Lp}(0)}{2}.$$ (40i)

In the aforementioned equations, *i*_*Lp*_(0) is zero and nine unknown variables are *t*_*on*_, *R*_*loss*_, *R*_*eq*(*pro*)_, *R*_*eq*(*proposed*)_, *u*_*Ca*_(0), *i*_*Lm*_(0), *u*_*Ca*_(*t*_*on*_), *i*_*Lp*_(*t*_*on*_), and *i*_*Lm*_(*t*_*on*_) respectively. The steady states can be reflected by applying the nine equations. In addition, it is -*u*_*Ca*_(0), *i*_*Lp*_(*t*_*on*_), and -*i*_*Lm*_(0) that are *U*_*Ca_peak*_, *I*_*Lp_peak*_, and *I*_*Lm_peak*_ respectively. Meanwhile, *I*_*Lf_av*_ and *U*_*C_av*_ can be shown as follows:

$$I_{Lf\_av}=\frac{NL_{M}[i_{Lm}(t_{on})-i_{Lm}(0)]}{t_{on}R_{eq(pro)}}$$ (40j)

$$U_{C\_av}=I_{Lf\_av}R.$$ (40k)

### 5.2 Analysis on the transient state

#### 5.2.1 Appropriate selection of variables

When the parameters of converter are sudden to change, such as input voltage or load, the converter will work under the transient state. If the new steady state after transient state is regarded as the reference, the old steady state before transient state can be as the initial state. Variables in the transient state analysis need to be appropriate so that the analysis is not high complexity and key information of converter can be effectively included. By means of the aforementioned typical FHA model, the peak voltage of C_a_, the peak current of L_P_ and L_M_, the average current of L_f_, and the equivalent average voltage of C between old and new steady state can be applied to analyze the transient state behaviors. The five variables just make up the indexes of large signal model.

#### 5.2.2 Simplified modified dynamic equations

The Figs 9 and 10 are still applicable for every half of switching period in the transient process except the R_eq(pro)_ and i_s1_ are different in the each period. It can be concluded that Fig 9 determines the transient properties of aforementioned variables because its duration is far longer than that of Fig 10. The equivalent circuit shown in Fig 9 can be treated as linear circuit when the R_eq(pro)_ is approximately averaged. Aforementioned indexes are the same between different analysis models under the steady state. The dynamic equations based on the FHA are benefit to analyze the transient process expediently. Considered the undesirable sinusoidal selectivity of LLC network, the correction coefficients related to the equivalent indexes need to be added in the proposed FHA model.

**Fig 11:**
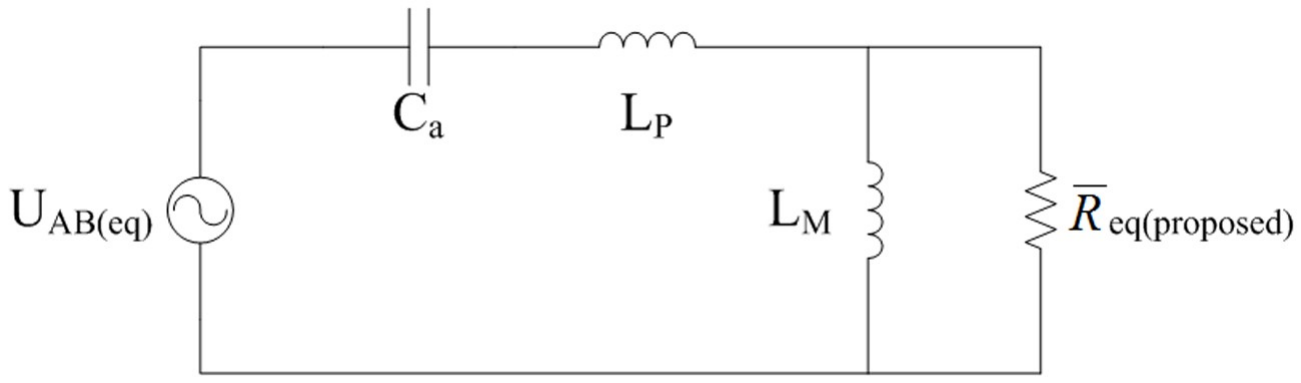
Proposed equivalent circuit based on the improved FHA. This is the proposed equivalent LLC circuit.

The definitions of variables are in accordance with that in the typical FHA model. Suppose that the correction coefficients from *k*_1_ to *k*_5_ are corresponding to the *u*_*Ca*_, *i*_*Lp*_, *i*_*Lm*_, *I*_*Lf*_, and *U*_*C*_ respectively. Proposed equivalent circuit can be seen in Fig 11 and it is originated from the typical FHA. Considered the invariance of zero-state response after step change of parameters, modified dynamic equations are obtained according to the principle that the indexes are approximate to the transient process which is only influenced by the characteristic equation of proposed equivalent circuit. Solving method of *k*_1_ to *k*_5_ can be seen in the next subsection.

The equivalent primary load in Fig 11 comes from R_eq(proposed)_ which can be expressed as follows in the transient process:

$$R_{eq(proposed)}=N^{2}[\frac{U_{C(eq)}+L_{f}k_{4}{\dot{I}}_{Lf}}{C{\dot{U}}_{C(eq)}+\frac{U_{C(eq)}}{R_{o(eq)}}}]$$ (41)

where R_o(eq)_ is the equivalent load of converter under the new steady state. R_o(eq)_ is solved in accordance with the (40a) to (40i) and it is equal to *R*_*eq*(*proposed*)_/*N*^2^ when the converter works under the steady state.

Imagine that the initial and final moments of transient state are *t*_0_ and *t*_2_ respectively. Average value of R_eq(proposed)_ in Fig 11 is presented as follows:

$${\bar{R}}_{eq(proposed)}=\frac{1}{t_{2}-t_{0}}\int_{t_{0}}^{t_{2}}N^{2}[\frac{U_{C(eq)}(\tau )+L_{f}k_{4}{\dot{I}}_{Lf}(\tau )}{C{\dot{U}}_{C(eq)}(\tau )+\frac{U_{C(eq)}(\tau )}{R_{o(eq)}}}]d\tau$$ (42)

where the two differential terms can be estimated in the light of relevant old and new steady state. Solving process of (42) is the same as that of (11).

Transient process of indexes can be approximately expressed as the positive envelopes of following state equations which the structure is similar to the (12):

$$\{\begin{array}{ll} k_{1}{\dot{u}}_{Ca} & =\frac{1}{C_{a}}k_{2}i_{Lp}\\ k_{2}{\dot{i}}_{Lp} & =\frac{1}{L_{P}}\left[-{\bar{R}}_{eq(proposed)}k_{2}i_{Lp}+{\bar{R}}_{eq(proposed)}k_{3}i_{Lm}-k_{1}u_{Ca}+\frac{4}{\pi }U_{in}\text{sin}(2\pi ft)\right]\\ k_{3}{\dot{i}}_{Lm} & =\frac{{\bar{R}}_{eq(proposed)}}{L_{M}}\left(k_{2}i_{Lp}-k_{3}i_{Lm}\right) \end{array}$$ (43)

where the initial values of states are the indexes under the old steady state.

### 5.3 Large signal model

In summary, the peak values of *u*_*Ca*_, *i*_*Lp*_, *i*_*Lm*_ and the average values of *I*_*Lf*_, *U*_*C*_ are the indexes respectively. The equivalent principle of indexes between aforementioned analyses and improved FHA model is the meaning that indexes are the same under the steady state and they are approximate under the transient state. Furthermore, a unified large signal model which is deduced from the typical FHA can be established according to the equivalent principle of indexes. The definitions of variables are the same as that in the (13a) to (13f). Nonlinear mathematical state equations are listed as follows:

$$C_{a}k_{1}{\dot{u}}_{Ca}=k_{2}i_{Lp}$$ (44a)

$$L_{M}k_{3}{\dot{i}}_{Lm}+L_{P}k_{2}{\dot{i}}_{Lp}+k_{1}u_{Ca}=\frac{4}{\pi }U_{in}\text{sin}(2\pi ft)$$ (44b)

$$k_{4}I_{Lf}=\frac{2}{T}\int_{t}^{t+\frac{T}{2}}\left|N(k_{2}i_{Lp}-k_{3}i_{Lm})\right|d\tau$$ (44c)

$$L_{f}k_{4}{\dot{I}}_{Lf}=\frac{2}{T}\int_{t}^{t+\frac{T}{2}}\frac{\left|L_{M}k_{3}{\dot{i}}_{Lm}\right|}{N}d\tau -U_{C(eq)}$$ (44d)

$$k_{4}I_{Lf}=C{\dot{U}}_{C(eq)}+\frac{U_{C(eq)}}{R_{o(eq)}}$$ (44e)

$$k_{5}U_{C}=\frac{\pi^{2}}{8}U_{C(eq)}$$ (44f)

where the *k*_1_ to *k*_5_ are obtained by combining the (5) to (9) and the solutions of indexes in the (40a) to (40k). When converter works under the steady state, aforementioned solving process of *k*_1_ to *k*_5_ is based on the equivalent principle of indexes under the steady state. Similarly, when the converter works under the transient state, the initial values of states are the indexes under the old steady state. Then, the indexes under the new steady state can be applied to the aforementioned solving process of *k*_1_ to *k*_5_ because transient process of converter is just determined by the characteristic equation of improved equivalent circuit. Indexes can be approximate to the transient process under solved *k*_1_ to *k*_5_. On the other hand, it complies with the equivalent principle of indexes between improved analyses and proposed model. Matlab needs to be used in the aforementioned process. After the *k*_1_ to *k*_5_ are known, the solving process of proposed model is the same as that of typical FHA model.

From what has been analyzed above, it can be concluded that the precision of large signal model based on the typical FHA and the proposed method is the accuracy of indexes under the steady state and transient state in the two models.

## 6 Design and selection of parameters

Abbreviations in this section are defined in Table 6 except that those have been appeared in aforementioned sections.

**Table 6:** Nomenclature.

Symbol	Definition	Symbol	Definition
**U**_**G1**_	Driving source of Q1	***R***_**(*P*)**_	AC resistance of wire at primary side
**R**_**11**_	Driving resistor of Q1	***R***_***(S)***_	AC resistance of wire at secondary side
**V**_**Z1**_	Zener diode of Q1	**U**_**p(source)**_	High frequency sinusoidal source
**R**_**12**_	Discharge resistor of Q1	**I**_**p(short)**_	Ideal ammeter
***C***_***GS(av)***_	Average value of *C*_*GS*_	**U**_**s(open)**_	Ideal voltmeter
***C***_***GD(av)***_	Average value of *C*_*GD*_	***I***_***o(max)***_	Maximum output current
***L***_***G***_	Parasitic inductance in the driving circuit	***f***_***max***_	Maximum switching frequency
***U***_***DS(max)***_	Maximum value of *u*_*DS*_	***U***_***o(n)***_	Nominal output voltage
***U***_***th***_	Threshold voltage of gate-source	***U***_***D(e)***_	Forward conduction voltage of rectifier diode
***t***_***f***_	Turn-off fall time	***δ***_***Lf***_	Air gap of L_f_
***U***_***in(max)***_	Maximum input voltage	***μ***_**0**_	Magnetic permeability of vacuum
***U***_***o(max)***_	Maximum output voltage	***A***_***e(Lf)***_	Effective cross-sectional area of L_f_
***f***_***min(n)***_	Allowed minimum switching frequency	***N***_***Lf***_	Turn number of L_f_
***A***_***e(Tr)***_	Effective cross-sectional area of transformer	***B***_***S(Lf)***_	Saturation magnetic flux density of L_f_
***B***_***m(Tr)***_	Maximum magnetic flux density of transformer	***D***_***wire(Lf)***_	Diameter of wire winded at L_f_
***N***_***P***_	Turn number of primary side	***L***_***wire(Lf)***_	Number of layers referred to the wire winded at L_f_
***N***_***S***_	Turn number of secondary side	**Δ**_***Lf***_	Penetration depth of wire winded at L_f_
***B***_***S(Tr)***_	Saturation magnetic flux density of transformer	***H***_***Lf***_	Height of window referred to L_f_
**Δ**_***Tr***_	Penetration depth of wire winded at transformer	***W***_***Lf***_	Width of window referred to L_f_
***γ***	Electrical conductivity of wire winded at transformer	***R***_***(Lf)***_	AC resistance of wire winded at L_f_
***μ***	Magnetic permeability of wire winded at transformer	**Δ*U***_***o(min)***_	Minimum voltage ripple of C
***D***_***wire(P)***_	Diameter of wire at primary side	***U***_***Q***_	Withstand voltage of MOSFET
***L***_***wire(P)***_	Number of layers at primary side	***I***_***Q***_	Withstand current of MOSFET
***H***_***Tr***_	Height of window referred to transformer	***U***_***VDR***_	Rated reverse voltage of related fast recovery diode
***D***_***wire(S)***_	Diameter of wire at secondary side	***I***_***VD***_	Rated forward current related fast recovery diode
***L***_***wire(S)***_	Number of layers at secondary side	***U***_***DR***_	Rated reverse voltage of rectifier diode
***W***_***Tr***_	Width of window referred to transformer	***I***_***D***_	Rated forward current of rectifier diode
***ρ***	Resistivity of wire referred to transformer		

**Fig 12:**
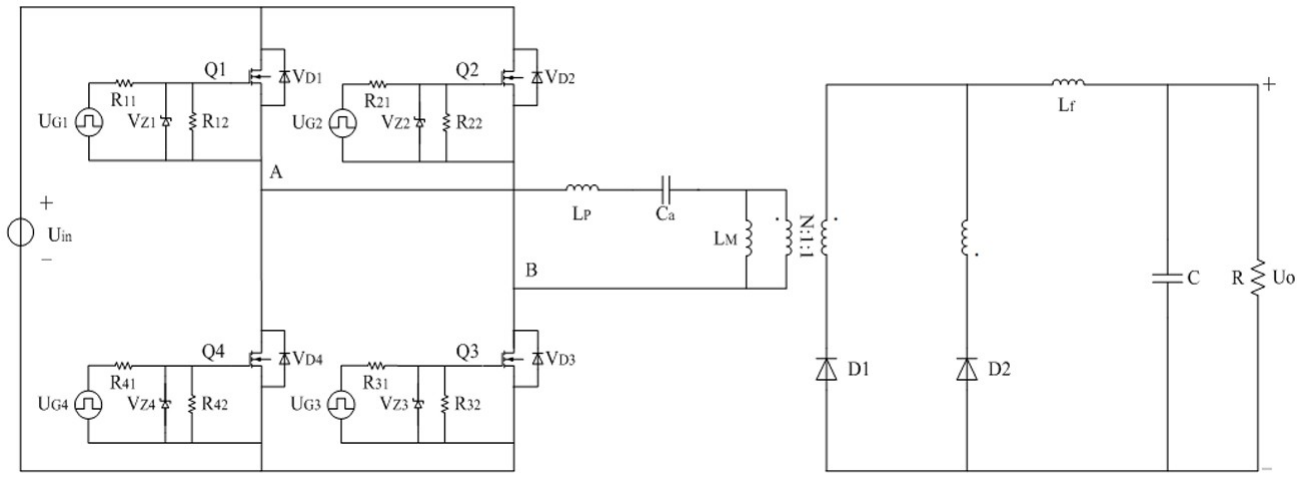
Schematic diagram of converter. This is the combination of driving circuit and main circuit.

Parameters of actual converter are designed and selected according to the Fig 12. It mainly contains driving circuit and main circuit. There are four same driving circuits except the driving sources. Every driving circuit is connected to the gate-source of corresponding MOSFET in parallel. Main circuit is the core of converter and it can be divided into six parts in the following description. Symbols of main circuit are the same as that in Fig 1.

### 6.1 Driving circuit

**Fig 13:**
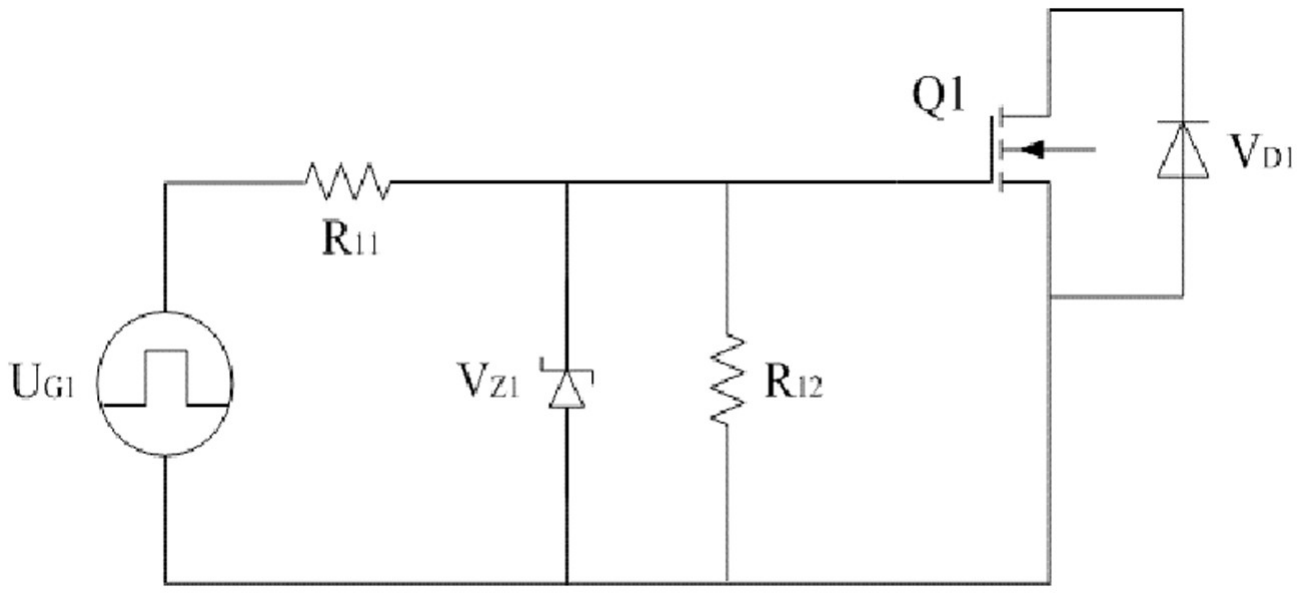
Driving circuit of Q1. This is the diagram of driving circuit.

Driving circuit of Q1 is taken as an example and it is shown in Fig 13. The driving source U_G1_ generates pulse signal and it comes from the TMS320F2812, optocoupler separation circuit, and amplifying circuit. R_11_ is the driving resistor which has effect on the switching action of Q1. V_Z1_ is the zener diode whose reverse voltage is the same as the amplitude of U_G1_. Generally, the value is fifteen volts. R_12_ is the discharge resistor for the gate of Q1 and it is about ten thousand ohms. The design and selection of R_11_ are formulated as follows.

R_11_ is related to the gate-source capacitance *C*_*GS*_ and gate-drain capacitance *C*_*GD*_. So *C*_*GS*_ and *C*_*GD*_ need to be fitted according to the datasheets. Both of them are the nonlinear functions of *u*_*DS*_ and the functions are symbolically shown as follows:

$$C_{GS}=f_{GS}(u_{DS});\;C_{GD}=f_{GD}(u_{DS}).$$ (45)

When the maximum value of *u*_*DS*_ is certain, the average values of *C*_*GS*_ and *C*_*GD*_ will be known and they are expressed as *C*_*GS*(*av*)_ and *C*_*GD*(*av*)_ respectively.

In order to obtain the enough damping, following inequality is listed so that the oscillation of driving current can be suppressed:

$$R_{11}\geq 2\sqrt{\frac{L_{G}}{C_{GS(av)}}}.$$ (46a)

In order to prevent the malfunction of Q1 when it turns off, the inequality can be listed as follows:

$$R_{11}\leq \frac{U_{th}}{C_{GD(av)}\frac{U_{DS(\text{max})}}{t_{f}}}.$$ (46b)

Based on the aforementioned two inequalities and the actual type of chip resistor, R_11_ can be obtained effectively.

### 6.2 Main circuit

Parameters of the main circuit are designed and selected according to the following six parts. They are isolated transformer, resonant capacitor, filter inductor, filter capacitor, MOSFETs and corresponding fast recovery diodes, and rectifier diodes respectively.

#### 1 Isolated transformer

In this paper, the bobbin of isolated transformer is type of EE. Parameters of transformer are designed and selected in the following order.

**Fig 14:**
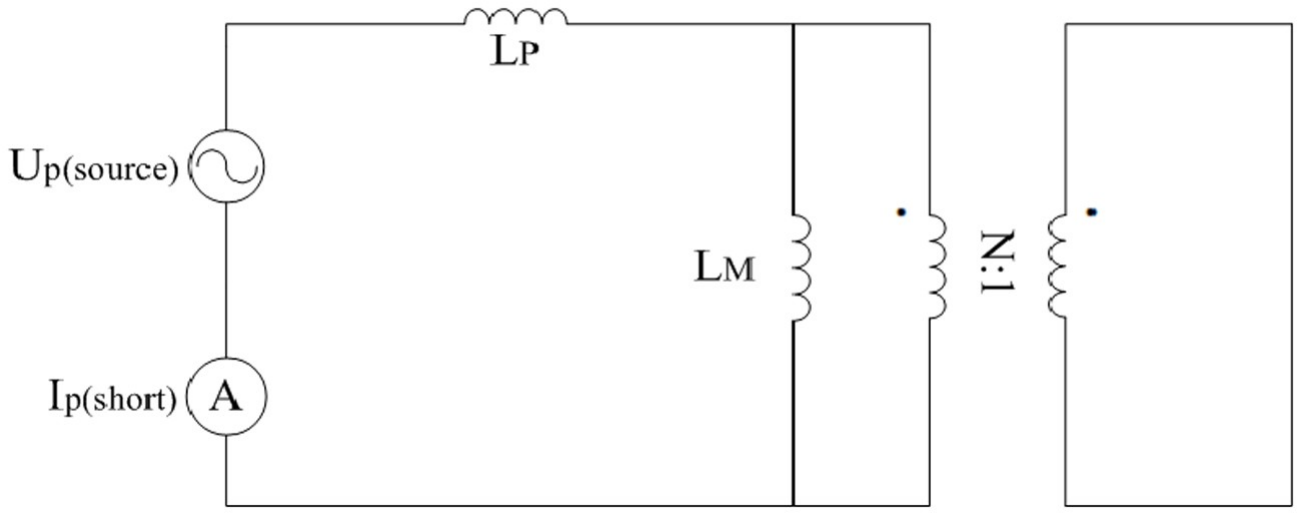
Short-circuit test. This is the schematic of short-circuit test.

**Fig 15:**
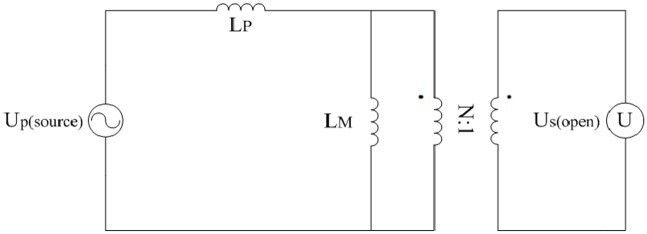
Open-circuit test. This is the schematic of open-circuit test.

1. Turn ratio *N*
  In order to make the output voltage can reach *U*_*o*(*max*)_ when the input voltage is *U*_*in*(*max*)_, following relationship can be obtained:
  $$N=\frac{U_{in(\text{max})}}{U_{o(\text{max})}}$$ (47)
  where *N* needs to be rounded.
2. The turn number of primary side and secondary side
  It is crucial that the output voltage can reach *U*_*o*(*max*)_ when the switching frequency is *f*_*min*(*n*)_. Meanwhile, *B*_*m*(*Tr*)_ cannot exceed *B*_*S*(*Tr*)_ so that magnetic saturation can be avoided. Following relationships are shown:
  $$N_{S}=\frac{U_{o(\text{max})}}{4f_{\text{min}(n)}A_{e(Tr)}B_{m(Tr)}};\;N_{P}=NN_{S};\;B_{m(Tr)}<B_{S(Tr)}$$ (48)
  where the *N*_*P*_ and *N*_*S*_ need to be rounded.
3. Wire of primary side
  Skin effect need to be considered because transformer works under the high frequency. In addition, height of windings cannot exceed *H*_*Tr*_. Following inequalities are shown:
  $$\Delta_{Tr}=\sqrt{\frac{1}{\pi f_{\text{min}(n)}\mu \gamma };}D_{wire(P)}\leq \;2\Delta_{Tr};\;\frac{N_{P}}{L_{wire(P)}}D_{wire(P)}<H_{Tr}.$$ (49)
4. Wire of secondary side
  Given the skin effect, relationships of them are similar to that of primary side and they are presented as follows:
  $$D_{wire(P)}<D_{wire(S)}\leq 2\Delta_{Tr};\;L_{wire(S)}=1;\;\frac{N_{S}}{L_{wire(S)}}D_{wire(S)}<H_{Tr}.$$ (50)
5. Verification of window area
  The windings must meet the following inequality. If it does not meet the expression, the wire has to be twined again.
  $$L_{wire(P)}D_{wire(P)}+2L_{wire(S)}D_{wire(S)}<W_{Tr}$$ (51)
6. AC resistance *R*_(*P*)_ and *R*_(*S*)_
  The AC resistances of wire at primary and secondary side need to be redefined according to Δ_*Tr*_ and DC resistance. They can be formulated as follows:
  $$R_{\left(P\right)}=\frac{\rho l_{wire(P)}}{\pi [{\left(\frac{D_{wire(P)}}{2}\right)}^{2}-{\left(\frac{D_{wire(P)}}{2}-\frac{66.1}{\sqrt{f_{\text{min}(n)}}}\right)}^{2}]}$$ (52a)
  $$R_{\left(S\right)}=\frac{\rho l_{wire(S)}}{\pi [{\left(\frac{D_{wire(S)}}{2}\right)}^{2}-{\left(\frac{D_{wire(S)}}{2}-\frac{66.1}{\sqrt{f_{\text{min}(n)}}}\right)}^{2}]}$$ (52b)
  where the units of *ρ* and *D*_*wire*(*P*)_ are Ω.mm^2^/m and mm respectively.
7. Leakage inductance *L*_*P*_ and excitation inductance *L*_*M*_
  A way of designing the LLC converter is proposed in [45] and [46]. However, the leakage inductance and excitation inductance of transformer are hardly regulated. So in this paper, the *L*_*P*_ and *L*_*M*_ need to be obtained by short-circuit test and open-circuit test which are shown in the following Figs 14 and 15. The U_p(source)_ is a high frequency sinusoidal source. The I_p(short)_ and U_s(open)_ are taken as ideal ammeter and voltmeter respectively.

Furthermore, the following equations are deduced from the aforementioned schematic diagrams. Values of *L*_*P*_ and *L*_*M*_ can be estimated according to the following equations and a large number of tests:

$$\{\begin{array}{l} L_{P}=\frac{U_{P(source)}}{2\pi fI_{P(open)}}\\ \frac{L_{M}}{L_{M}+L_{P}}=\frac{NU_{S(open)}}{U_{P(source)}} \end{array}.$$ (53)

#### 2 Resonant capacitor *C*_*a*_

In order to make the output current always lower than the *I*_*o*(*max*)_ when the switching frequency is *f*_*max*_ and voltage C_a_ reaches half of *U*_*in*(*max*)_, the C_a_ is selected according to the following equation:

$$C_{a}=\frac{I_{o(\text{max})}}{2Nf_{\text{max}}\times U_{in(\text{max})}}$$ (54)

where *C*_*a*_ is obtained in the way of worst case analysis.

#### 3 Filter inductor *L*_*f*_

The bobbin of filter inductor is also type of EE. Parameters of inductor are designed and selected in the following order.

1. Inductance
  Value of *L*_*f*_ is calculated according to the following equation so that the current ripple is not more than ten percent of *I*_*o*(*max*)_ when the switching frequency, input voltage, and output voltage are *f*_*min*(*n*)_, *U*_*in*(*max*)_, and *U*_*o*(*n*)_ respectively:
  $$L_{f}=\frac{1}{2f_{\text{min}(n)}\times (10\%I_{o(\text{max})})}\left[\left(\frac{U_{in(\text{max})}}{N}-U_{D(e)}\right)-U_{o(n)}\right]$$ (55)
  where *L*_*f*_ is obtained in the way of worst case analysis.
2. The turn number
  Based on the definition of inductance with air gap, following relationship is shown:
  $$N_{Lf}=\sqrt{\frac{L_{f}\delta_{Lf}}{\mu_{0}A_{e(Lf)}}}$$ (56)
  where the *N*_*Lf*_ needs to be rounded.
3. Verification of *B*_*S*(*Lf*)_
  The theoretical magnetic flux density must meet the following equation so that *L*_*f*_ is not magnetic saturation. If it does not meet the expression, *δ*_*Lf*_ needs to be modified again.
  $$\frac{\mu_{0}N_{Lf}I_{o(\text{max})}}{\delta_{Lf}}<B_{S(Lf)}$$ (57)
4. Wire
  It is important to consider the skin effect and height of windings. Relationships of relevant variables are similar to that of transformer and they are shown as follows:
  $$\Delta_{Lf}=\sqrt{\frac{1}{2\pi f_{\text{min}(n)}\mu \gamma }};\;D_{wire(Lf)}\leq \;2\Delta_{Tr};\;\frac{N_{Lf}}{L_{wire(Lf)}}D_{wire(Lf)}<H_{Lf}.$$ (58)
5. Verification of window area
  The windings must satisfy the following inequality. If it does not meet the relationship, the wire has to be twined again.
  $$L_{wire(Lf)}D_{wire(Lf)}<W_{Lf}$$ (59)
6. AC resistance *R*_(*Lf*)_
  The AC resistance of wire is redefined in the light of Δ_*Lf*_ and DC resistance. So it can be formulated as follows:
  $$R_{\left(Lf\right)}=\frac{\rho l_{wire(Lf)}}{\pi [{\left(\frac{D_{wire(Lf)}}{2}\right)}^{2}-{\left(\frac{D_{wire(Lf)}}{2}-\frac{66.1\sqrt{2}}{\sqrt{f_{\text{min}(n)}}}\right)}^{2}]}$$ (60)
  where the units of *ρ* and *D*_*wire*(*Lf*)_ are Ω.mm^2^/m and mm respectively.

#### 4 Filter capacitor *C*

Value of *C* is obtained in accordance with the following equation so that the voltage ripple is not more than the standard ripple 0.2V when the switching frequency, input voltage, and output voltage are *f*_*min*(*n*)_, *U*_*in*(*max*)_, and *U*_*o*(*n*)_ respectively:

$$C=\frac{1}{8L_{f}{\left(2f_{\text{min}(n)}\right)}^{2}\times \Delta U_{o(\text{min})}}\left[\left(\frac{U_{in(\text{max})}}{N}-U_{D(e)}\right)-U_{o(n)}\right]$$ (61)

where *C* is obtained in the way of worst case analysis.

#### 5 MOSFETs Q1~Q4 and fast recovery diodes V_D1_~V_D4_

*U*_*Q*_, *I*_*Q*_, *U*_*VDR*_, and *I*_*VD*_ come from datasheets based on the specific type of MOSFET and fast recovery diode respectively. In order to set aside enough margins, the following inequalities need to be satisfied:

$$U_{Q}>2U_{in(\text{max})};\;I_{Q}>2\left(\frac{I_{o(\text{max})}}{N}+\frac{NU_{o(\text{max})}f_{\text{max}}}{L_{M}}\right);$$ (62a)

$$U_{VDR}>2U_{in(\text{max})};\;I_{VD}>2\left(\frac{I_{o(\text{max})}}{N}+\frac{NU_{o(\text{max})}f_{\text{max}}}{L_{M}}\right).$$ (62b)

#### 6 Rectifier diodes D1 and D2

*U*_*DR*_ and *I*_*D*_ come from datasheets based on the specific type of Schottky rectifier diode respectively. They need to meet the following inequalities so that enough margins can be set aside:

$$U_{DR}>2(\frac{2U_{in(\text{max})}}{N});\;I_{D}>2I_{o(\text{max})}.$$ (63)

In this paper, aforementioned parameters are listed in Tables 7 and 8 to 13. Furthermore, all the relationships mentioned above are applied so that the specific components can be obtained. Main specifications of transformer and filter inductor are same and it is listed in Table 7 individually. Values of other components are shown in Tables 8 to 13.

**Table 7:** Specifications of transformer and filter inductor.

Name	Specification
Core material	PC40
Size of core	EE55
B_S_	0.51T
A_e_	354mm^2^
H	37.6mm
W	10.28mm

**Table 8:** Values of components.

Symbol	U_in(max)_	U_o(max)_	f_min(n)_	N_S_	N_P_	B_m(Tr)_	μ	γ
Value	140	15	23	6	54	0.08	4π×10^−7^	5.8×10^7^

The unit of f_min(n)_ is kHz. Other physical quantities are all used the international system of units.

**Table 9:** Values of components.

Symbol	Δ_Tr_	D_wire(P)_	L_wire(P)_	D_wire(S)_	ρ	l_wire(P)_	l_wire(S)_	R_(P)_	R_(S)_
Value	0.77	1.04	3	1.04	0.0175	4.49	0.72	0.0941	0.0154

The units of Δ_Tr_, D_wire(P)_, D_wire(S)_, and ρ are mm, mm, mm, and Ω.mm^2^/m respectively. Other physical quantities are all used the international system of units.

**Table 10:** Values of components.

Symbol	U_o(n)_	U_D(e)_	I_o(max)_	f_max_	δ_Lf_	μ_0_	N_Lf_	Δ_Lf_	D_wire(Lf)_
Value	12	0.5	3	30	0.7	4π×10^−7^	20	0.55	1.04

The units of f_max_, δ_Lf_, Δ_Lf_, and D_wire(Lf)_ are kHz, mm, mm, and mm respectively. Other physical quantities are all used the international system of units.

**Table 11:** Values of components.

Symbol	L_wire(Lf)_	l_wire(Lf)_	R_(Lf)_	ΔU_o(min)_	U_DS(max)_	C_GS(av)_	C_GD(av)_
Value	1	1.69	0.0356	1	140	2428	228

The units of ΔU_o(min)_, C_GS(av)_, and C_GD(av)_ are mV, pF, and pF respectively. Other physical quantities are all used the international system of units.

**Table 12:** Values of components.

Symbol	L_G_	U_th_	t_f_	R_11_	R_12_
Value	20	3	75	6.2	10

The units of L_G_, t_f_, and R_12_ are nH, ns, and kΩ respectively. Other physical quantities are all used the international system of units.

**Table 13:** Values of components.

Symbol	*U*_*Q*_	*I*_*Q*_	*U*_*VDR*_	*I*_*VD*_	*U*_*DR*_	*I*_*D*_
Value	500	20	600	15	100	25

According to aforementioned design and selection of parameters, the actual converter can be obtained. Main components and values are summed up in Table 14. Following numerical verifications are also presented in accordance with this table.

**Table 14:** Parameters of actual converter.

Name	Value/Type
MOSFETs Q1~ Q4	IRFP 460
Fast recovery diodes	MUR3060WT
Block capacitor C_a_	47nF
^1^AC resistance R_P_	1.5149Ω
Leakage inductance L_P_	550μH
Excitation inductance L_M_	12.2mH
Number N	9
AC resistance R_S_	0.0152Ω
Schottky diodes D1 and D2	V50100PW
Filter inductor L_f_	250μH
^2^AC resistance R_Lf_	0.1361Ω
Filter capacitor C	680 μF

^1^ ESR of C_a_ and current sampling resistor are 0.42Ω and 1Ω respectively.

^2^ The current sampling resistor is 0.1Ω.

## 7 Numerical results

According to the Fig 4, main parameters of actual converter are listed in Table 14. It includes the main body of converter except for the switching frequency, input voltage, and load. The later three parameters will be given in Table 15 individually so that the different working conditions described from A1 to A8 can be well presented. In addition, the work region satisfies the (18).

**Table 15:** Different working conditions.

States	Switching frequency (kHz)	Input voltage (V)	Load (Ω)
A1	24	100	24
A2	24	120	24
A3	24	100	18
A4	24	120	18
A5	30	100	24
A6	30	120	24
A7	30	100	18
A8	30	120	18

The following numerical results are classified according to the steady state and transient state in the different models and working conditions. Evaluation criteria of typical FHA model and proposed model are the accuracy of indexes. So the results of key indexes mentioned in the previous section are shown as follows.

### 7.1 Typical FHA model

#### 7.1.1 Results of steady state

When the conclusions from (5) to (9) are applied, the key indexes under different working conditions can be obtained and they are shown in Table 16. They are also the solutions of large signal model from (13a) to (13f) under the steady state. Given that *I*_*Lm_peak*_ cannot be measured directly and it has to be replaced by *I*_*Lp_peak*_*−I*_*Lf_av*_/*N* in the actual converter, so *I*_*Lm_peak*_ are not necessary to be listed in the following sections.

**Table 16:** Key indexes under different working conditions.

States	*U*_*Ca_peak*_(V)	*I*_*Lp_peak*_(A)	*U*_*C_av*_(V)	*I*_*Lf_av*_(A)
A1	12.1675	0.1098	11.4656	0.4777
A2	14.5944	0.1317	13.7587	0.5733
A3	14.6276	0.1320	11.4589	0.6366
A4	17.5532	0.1584	13.7507	0.7639
A5	8.7146	0.0983	11.1556	0.4648
A6	10.4610	0.1180	13.3867	0.5578
A7	10.7802	0.1216	11.1556	0.6198
A8	12.9344	0.1459	13.3867	0.7437

#### 7.1.2 Results of transient state

R_eq0(FHA)_ represents the R_eq(FHA)_ under old steady state and R_eq1(FHA)_ represents the R_eq(FHA)_ under new steady state. When working conditions are sudden to change from A1 to A3, from A2 to A4, from A5 to A7, from A6 to A8 respectively, following relationships derived from the (11) can be concluded according to the old steady state and new steady state shown in Table 16:

$$L_{f}{\dot{I}}_{Lf}<<U_{C(eq)};\;C{\dot{U}}_{C(eq)}<<\frac{U_{C(eq)}}{\frac{8}{\pi^{2}}R};\;{\bar{R}}_{eq(FHA)}\approx R_{eq1(FHA)}.$$ (64)

Aforementioned corresponding transient states are named after state (a), state (b), state (c), and state (d) respectively. Peak values of *u*_*Ca*_ and *i*_*Lp*_ in the transient process can be expressed by the *u*_*Ca*_*peak*_ and *i*_*Lp*_*peak*_ respectively. Δ*t* is coincident with the transition time of *u*_*Ca*_*peak*_ and *i*_*Lp*_*peak*_. Based on the (12), the approximate expressions of *u*_*Ca*_*peak*_ and *i*_*Lp*_*peak*_ are formulated as follows.

State (a) $$u_{Ca\_peak}:-2.4601e^{-8300.4978t}+14.6276$$ (65a)
  $$i_{Lp\_peak}:-0.0222e^{-8300.4978t}+0.1320$$ (65b)
State (b) $$u_{Ca\_peak}:-2.9588e^{-8300.4978t}+17.5532$$ (66a)
  $$i_{Lp\_peak}:-0.0267e^{-8300.4978t}+0.1584$$ (66b)
State (c) $$u_{Ca\_peak}:-2.0656e^{-8300.4978t}+10.7802$$ (67a)
  $$i_{Lp\_peak}:-0.0233e^{-8300.4978t}+0.1216$$ (67b)
State (d) $$u_{Ca\_peak}:-2.4734e^{-8300.4978t}+12.9344$$ (68a)
  $$i_{Lp\_peak}:-0.0279e^{-8300.4978t}+0.1459$$ (68b)

The transition time is 0.8322 milliseconds unanimously. These expressions can be taken as the approximate positive envelopes of large signal model from (13a) to (13f) under the transient state.

### 7.2 Proposed model

**Fig 16:**
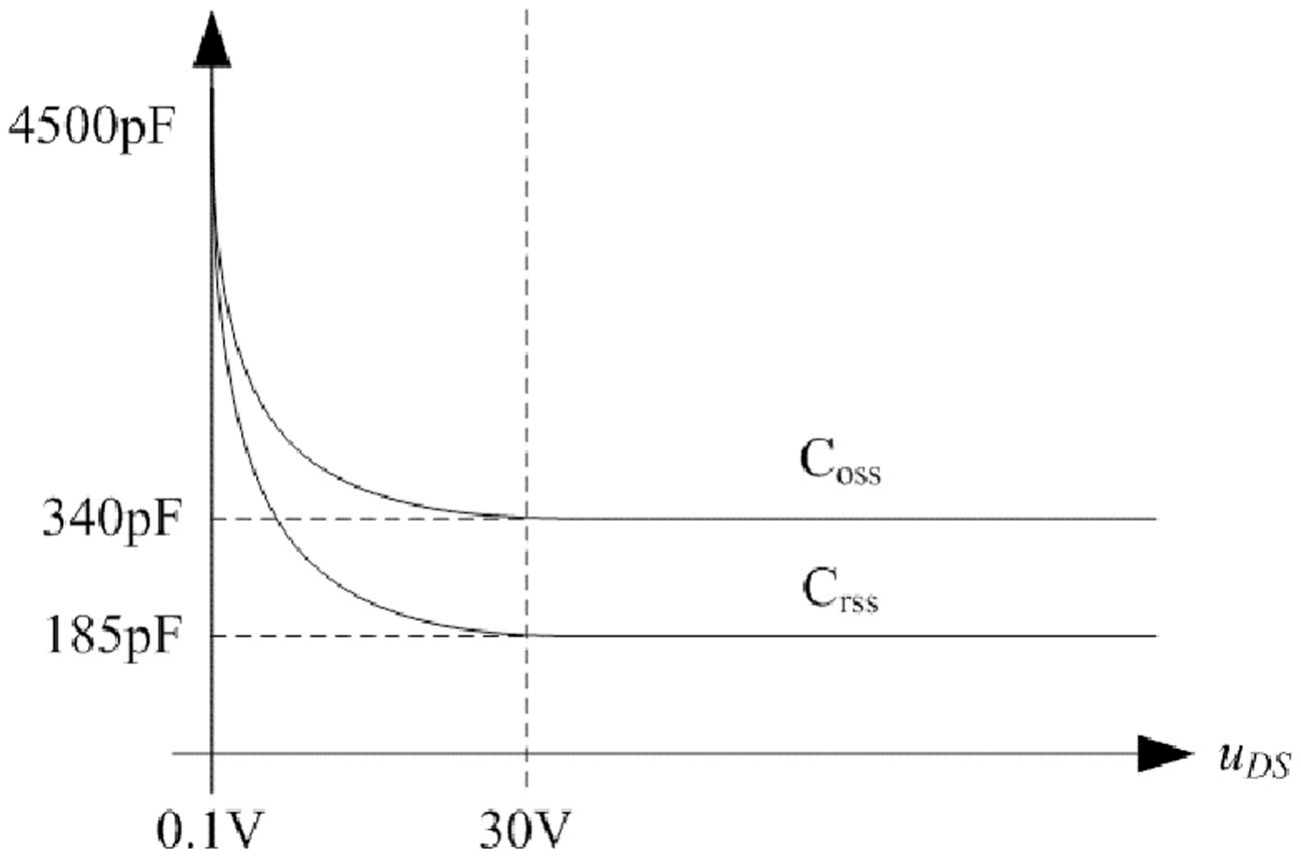
Relationships between C_oss_, C_rss_, and *u*_*DS*_. This is the relationship in the MOSFET.

In order to obtain the typical drain-source capacitance C_DS_ of IRFP 460, relationships between C_oss_, C_rss_, and *u*_*DS*_ are shown in Fig 16 according to the corresponding datasheet.

Following piecewise function is obtained by fitting the two curves in Fig 16:

$$C_{DS}=\{\begin{array}{l} 10^{-8.7996}{\left(u_{DS}\right)}^{-0.4528}-10^{-8.9063}{\left(u_{DS}\right)}^{-0.5595},\;\;\;\;0V\;<\;u_{DS}\;<\;30V\\ 1.55\times 10^{-10},\;\;\;\;u_{DS}\geq \;30V \end{array}.$$ (69)

**Fig 17:**
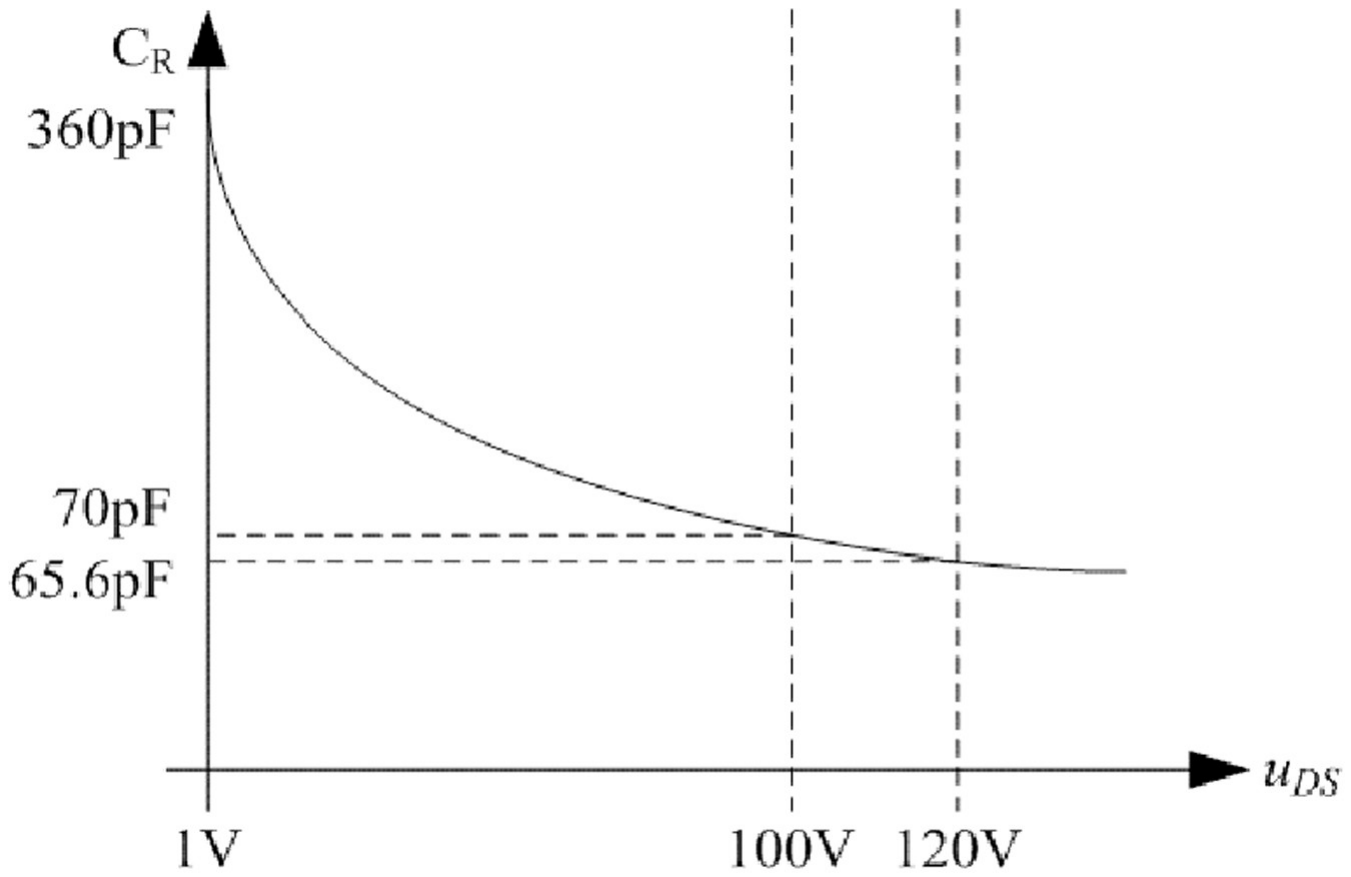
Relationship between C_R_ and *u*_*DS*_. This is the relationship in the fast recovery diode.

Relationship between C_R_ and *u*_*DS*_ is shown in Fig 17 according to the corresponding datasheet of MUR3060WT. Furthermore, the (32) can be applied so that the mathematical relation is clear.

Following function is obtained by fitting the two curves in Fig 17:

$$C_{R}=(3.6\;\times \;10^{-10}){\left(u_{DS}\right)}^{-0.3556}.$$ (70)

The C_eq_ in (33) can be operated by combining (69) and (70). When the input voltage is 100V, the C_eq_ is 277.55pF. When the input voltage is 120V, the C_eq_ is 268.41pF.

**Fig 18:**
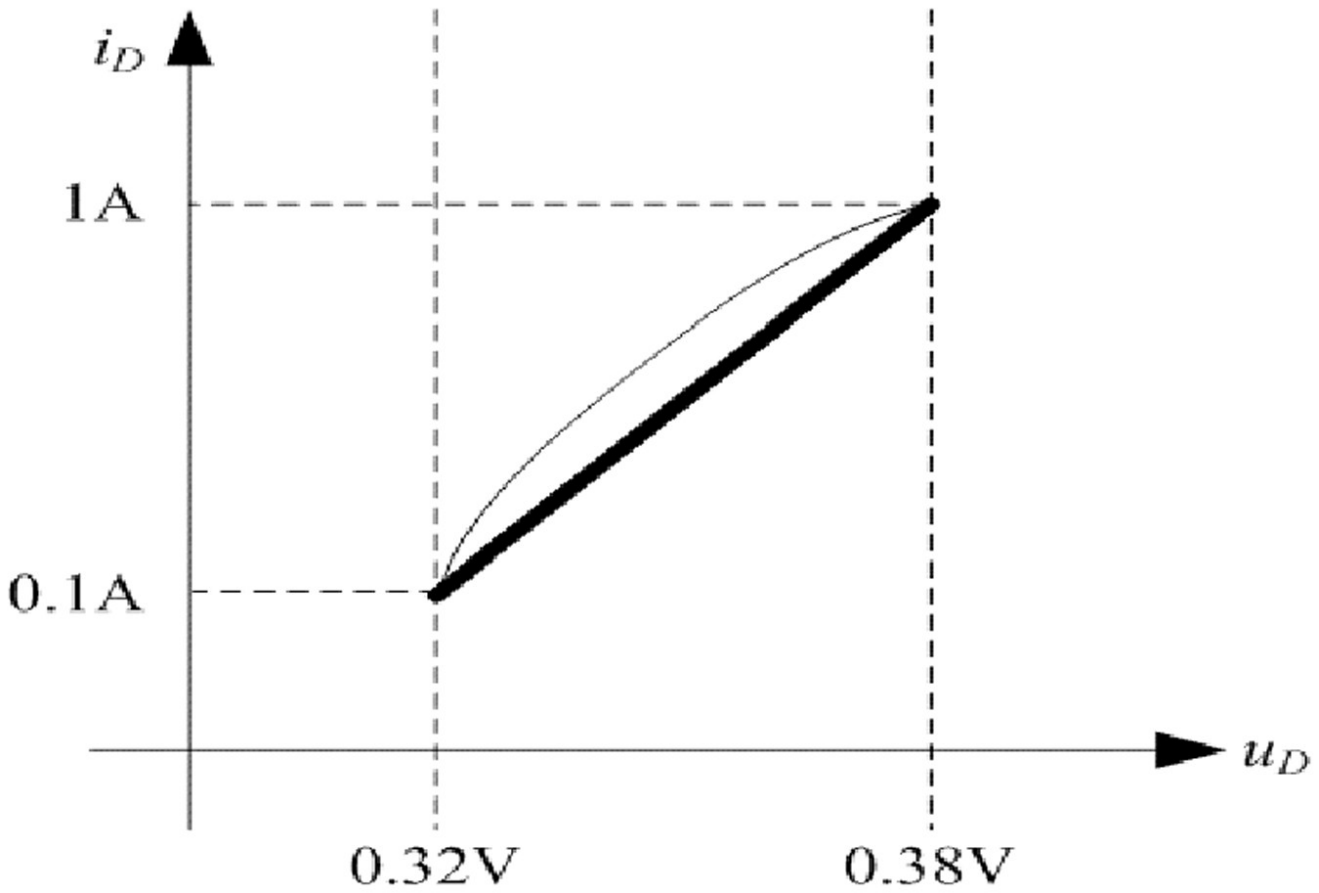
Forward volt-ampere characteristic of V50100PW. This is the relationship in the rectifier diode.

Forward volt-ampere characteristic of V50100PW is shown in Fig 18 according to the relevant datasheet. Then, the (37) can be fitted based on the actual curve. Coarse line in Fig 18 is the fitting result which represents the linear relationship between *i*_*D*_ and *u*_*D*_. Furthermore, the *k* and *b* in (37) are 0.0667 and 0.3133 respectively.

#### 7.2.1 Results of steady state

When the algebraic equations presented from (40a) to (40i) are solved and the equivalent principle of indexes is applied to the large signal model from (44a) to (44f) under the steady state, correction coefficients and R_o(eq)_, *t*_*on*_ shown in Fig 3, and key indexes under different working conditions can be obtained respectively. These numerical results are shown in Tables 17, 18 and 19 respectively.

**Table 17:** Correction coefficients and R_o(eq)_ under different working conditions.

States	*k*_1_	*k*_2_	*k*_3_	*k*_4_	*k*_5_	R_o(eq)_ (Ω)
A1	1.0330	1.0333	0.8665	1.4972	1.1062	14.3742
A2	1.0368	1.0371	0.8646	1.4778	1.1003	14.4848
A3	0.9438	0.9440	0.8665	1.1045	1.1128	14.6985
A4	0.9454	0.9451	0.8665	1.0900	1.1072	14.8219
A5	0.8938	0.8936	0.8567	1.2584	1.0850	16.7733
A6	0.8972	0.8975	0.8551	1.2434	1.0793	16.8850
A7	0.8127	0.8129	0.8580	0.9276	1.0923	17.1805
A8	0.8145	0.8144	0.8562	0.9160	1.0865	17.3067

**Table 18:** The *t*_*on*_ under different working conditions.

States	*t*_*on*_(μs)
A1	20.1339
A2	20.1518
A3	20.1193
A4	20.1371
A5	15.8479
A6	15.8696
A7	15.8294
A8	15.8511

**Table 19:** Key indexes under different working conditions.

States	*U*_*Ca_peak*_(V)	*I*_*Lp_peak*_(A)	*U*_*C_av*_(V)	*I*_*Lf_av*_(A)
A1	14.3111	0.1291	10.3584	0.4316
A2	17.0156	0.1535	12.4968	0.5207
A3	15.4280	0.1392	10.2978	0.5721
A4	18.3684	0.1658	12.4200	0.6900
A5	10.8316	0.1222	10.2816	0.4284
A6	12.8848	0.1453	12.4032	0.5168
A7	11.7041	0.1320	10.2132	0.5674
A8	13.9420	0.1573	12.3210	0.6845

#### 7.2.2 Results of transient state

R_eq0(proposed)_ represents the R_eq(proposed)_ under old steady state and R_eq1(proposed)_ represents the R_eq(proposed)_ under new steady state. When the working conditions are sudden to change from A1 to A3, from A2 to A4, from A5 to A7, from A6 to A8 respectively, following relationships derived from the (42) can be generalized according to the old steady state and new steady state presented in Table 19:

$$L_{f}k_{4}{\dot{I}}_{Lf}<<U_{C(eq)};\;C{\dot{U}}_{C(eq)}<<\frac{U_{C(eq)}}{R_{o(eq)}};\;{\bar{R}}_{eq(proposed)}\approx R_{eq1(proposed)}.$$ (71)

The meanings of state (a), state (b), state (c), state (d), *u*_*Ca*_*peak*_ and *i*_*Lp*_*peak*_ are the same as that of typical FHA model in the transient process. Similarly, Δ*t* is coincident with the transition time of *u*_*Ca*_*peak*_ and *i*_*Lp*_*peak*_. Based on the (43), the approximate expressions of *u*_*Ca*_*peak*_ and *i*_*Lp*_*peak*_ are as follows.

State (a) $$u_{Ca\_peak}:-1.1169e^{-8238.4509t}+15.4280$$ (72a)
  $$i_{Lp\_peak}:-0.0101e^{-8238.4509t}+0.1392$$ (72b)
  The transition time is 0.8385 milliseconds.
State (b) $$u_{Ca\_peak}:-1.3528e^{-8168.8592t}+18.3684$$ (73a)
  $$i_{Lp\_peak}:-0.0123e^{-8168.8592t}+0.1658$$ (73b)
  The transition time is 0.8456 milliseconds.
State (c) $$u_{Ca\_peak}:-0.8725e^{-7034.9679t}+11.7041$$ (74a)
  $$i_{Lp\_peak}:-0.0098e^{-7034.9679t}+0.1320$$ (74b)
  The transition time is 0.9819 milliseconds.
State (d) $$u_{Ca\_peak}:-1.0572e^{-6983.1499t}+13.9420$$ (75a)
  $$i_{Lp\_peak}:-0.0120e^{-6983.1499t}+0.1573$$ (75b)
  The transition time is 0.9892 milliseconds.

These expressions can be taken as the approximate positive envelopes of large signal model from (44a) to (44f) under the transient state.

## 8 Experimental results

Actual full-bridge LLC converter has been designed and the main parameters are shown in Table 14. Working conditions verified by the experiment are the same as that in Table 15. According to the theoretical analyses and previous numerical results, the results of experimental verification are corresponding to the steady state and transient state respectively.

### 8.1 Results of steady state

In terms of input voltage and load at the same switching frequency, the working conditions A1 and A4 can be taken as the boundaries of A2 and A3 respectively. Similarly, the working conditions A5 and A8 can be also treated as the boundaries of A6 and A7 respectively. So the experimental results of key variables related to A1, A4, A5, and A8 are given under the steady state.

#### A1

**Fig 19:**
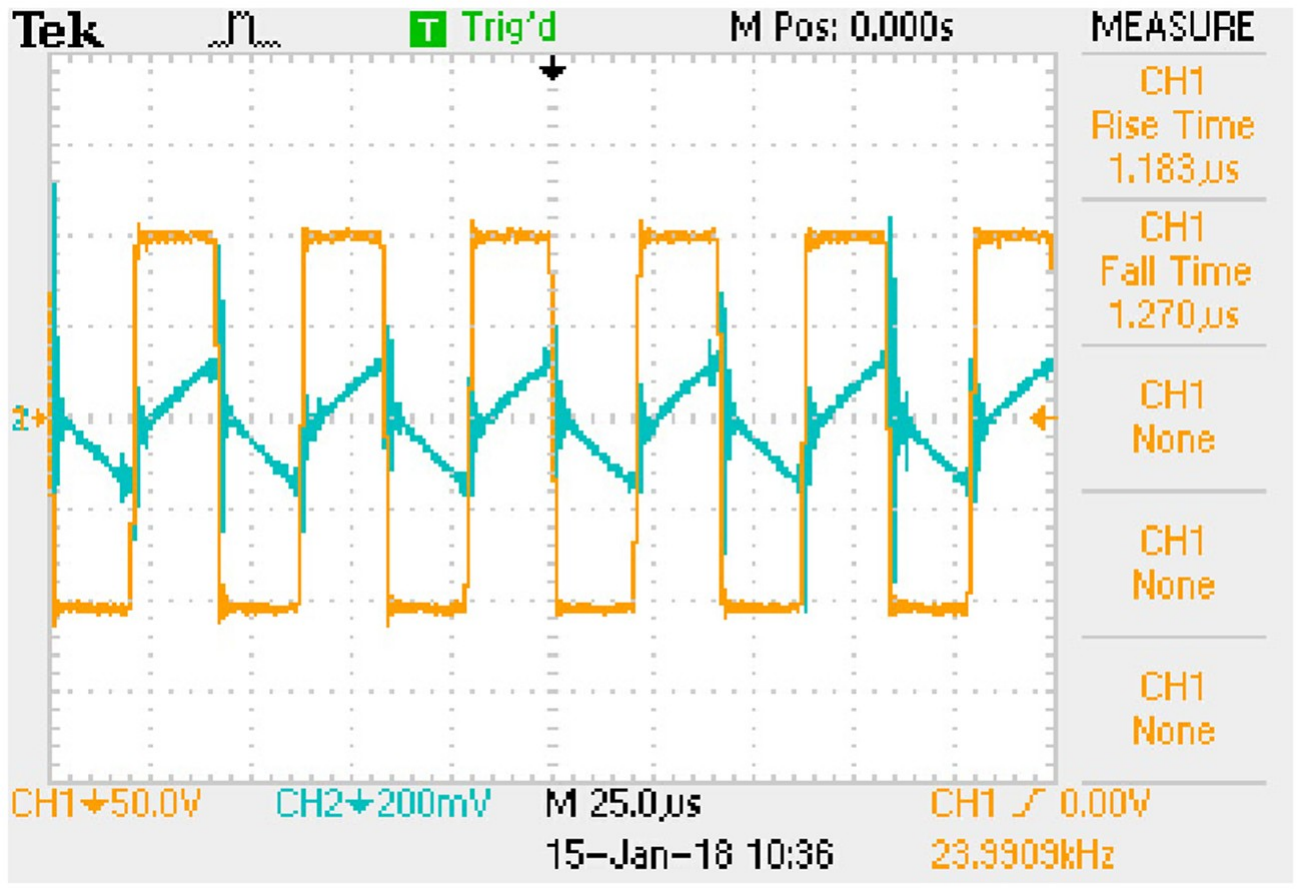
Waveform of key variables: *u*_*AB*_ (CH1) and *i*_*Lp*_ (CH2). This is the measured signal.

**Fig 20:**
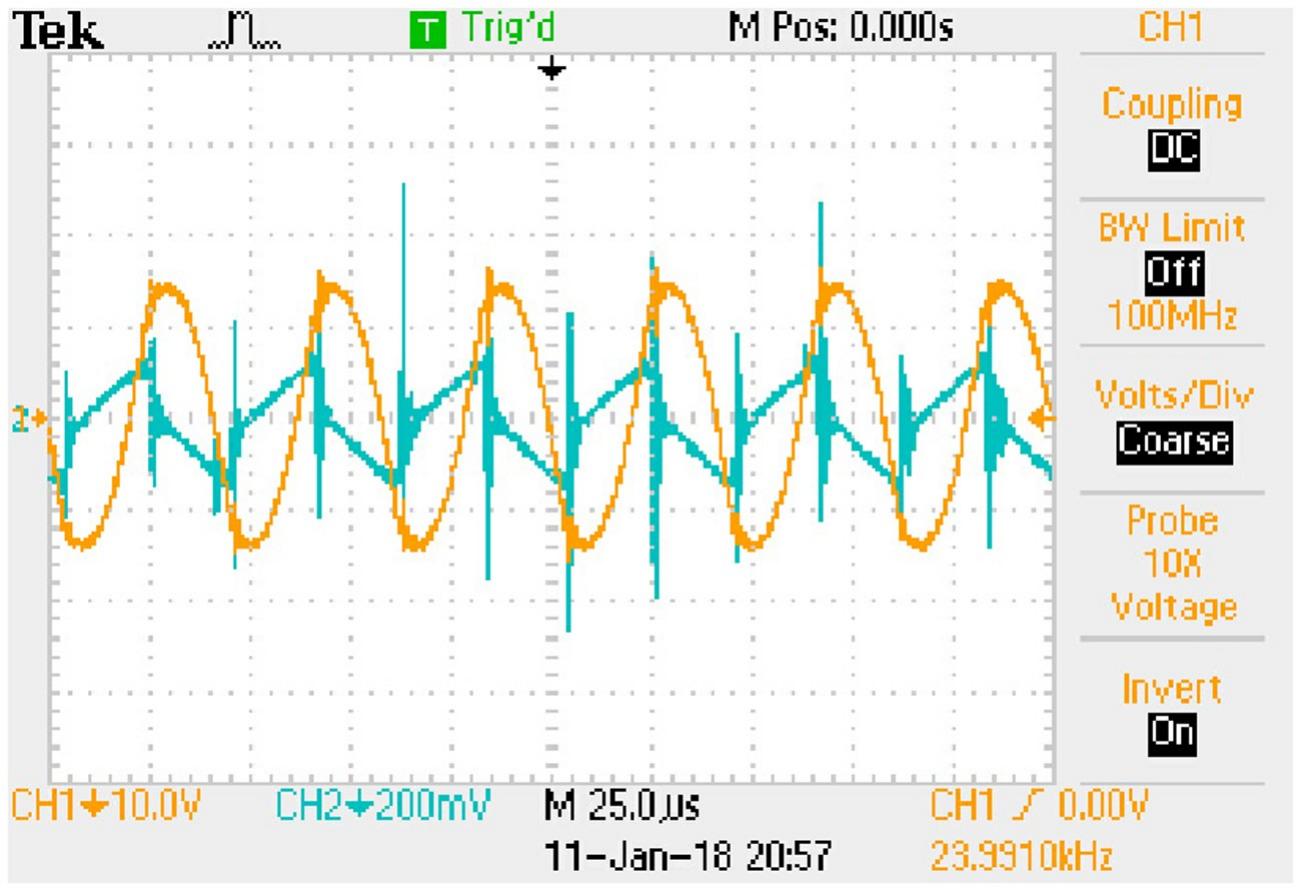
Waveform of key variables: *u*_*Ca*_ (CH1) and *i*_*Lp*_ (CH2). This is the measured signal.

**Fig 21:**
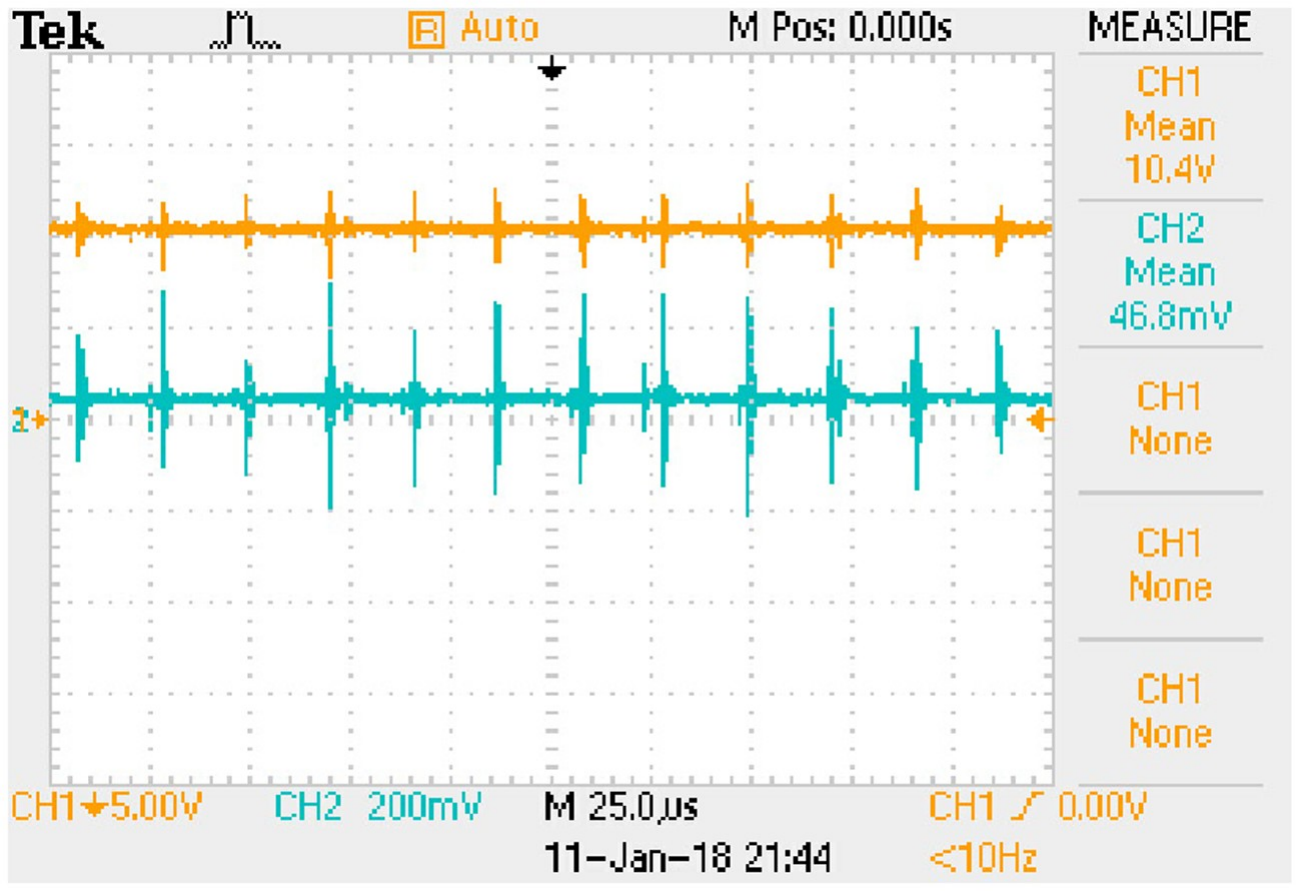
Waveform of key variables: *U*_*C*_ (CH1) and *I*_*Lf*_ (CH2). This is the measured signal.

Under the A1 condition, the *u*_*AB*_ and *i*_*Lp*_ can be seen in Fig 19. The *u*_*Ca*_ and *i*_*Lp*_ can be seen in Fig 20. The *U*_*C*_ and *I*_*Lf*_ can be seen in Fig 21.

The *i*_*Lp*_ is measured by 1Ω sampling resistor and the *I*_*Lf*_ is measured by 0.1Ω sampling resistor.

#### A4

**Fig 22:**
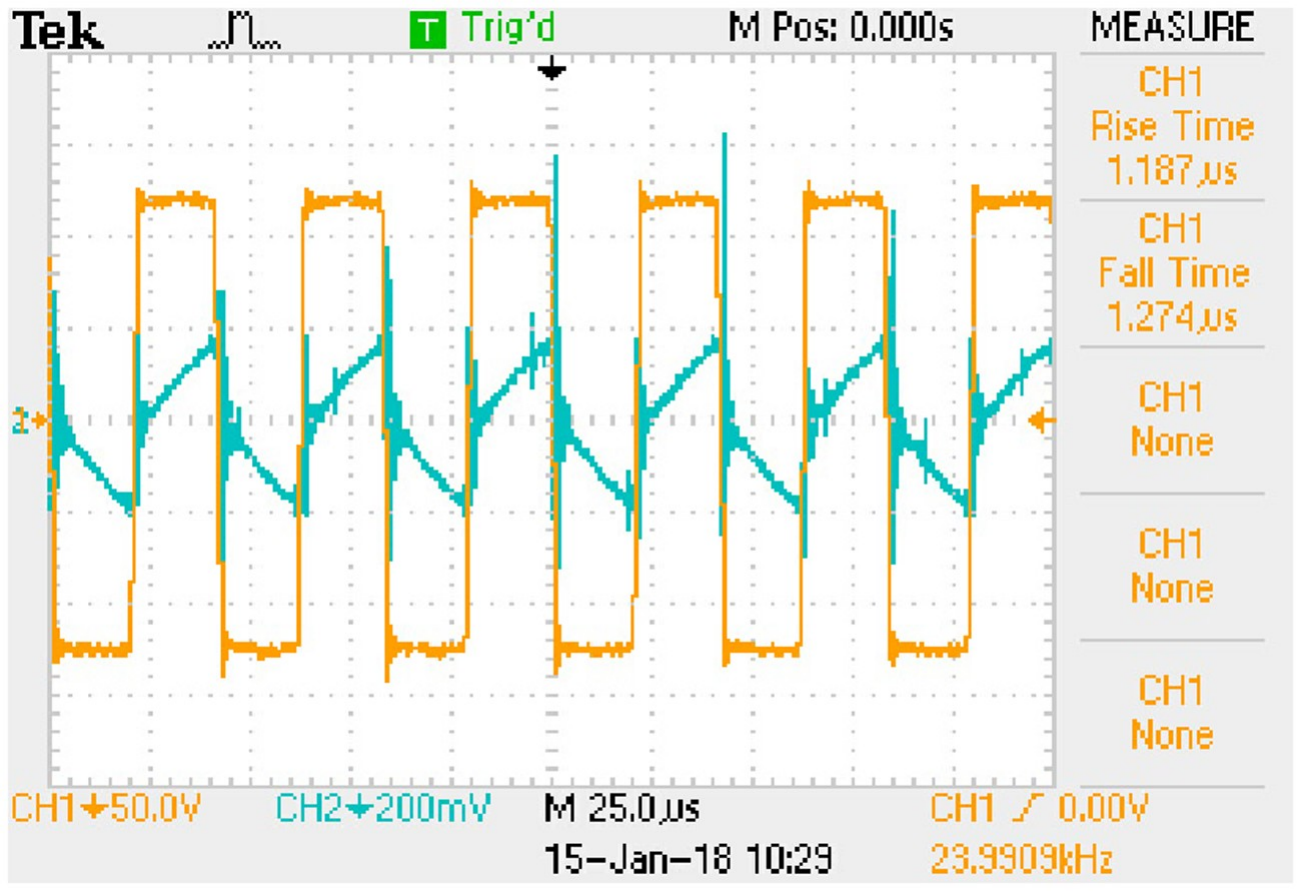
Waveform of key variables: *u*_*AB*_ (CH1) and *i*_*Lp*_ (CH2). This is the measured signal.

**Fig 23:**
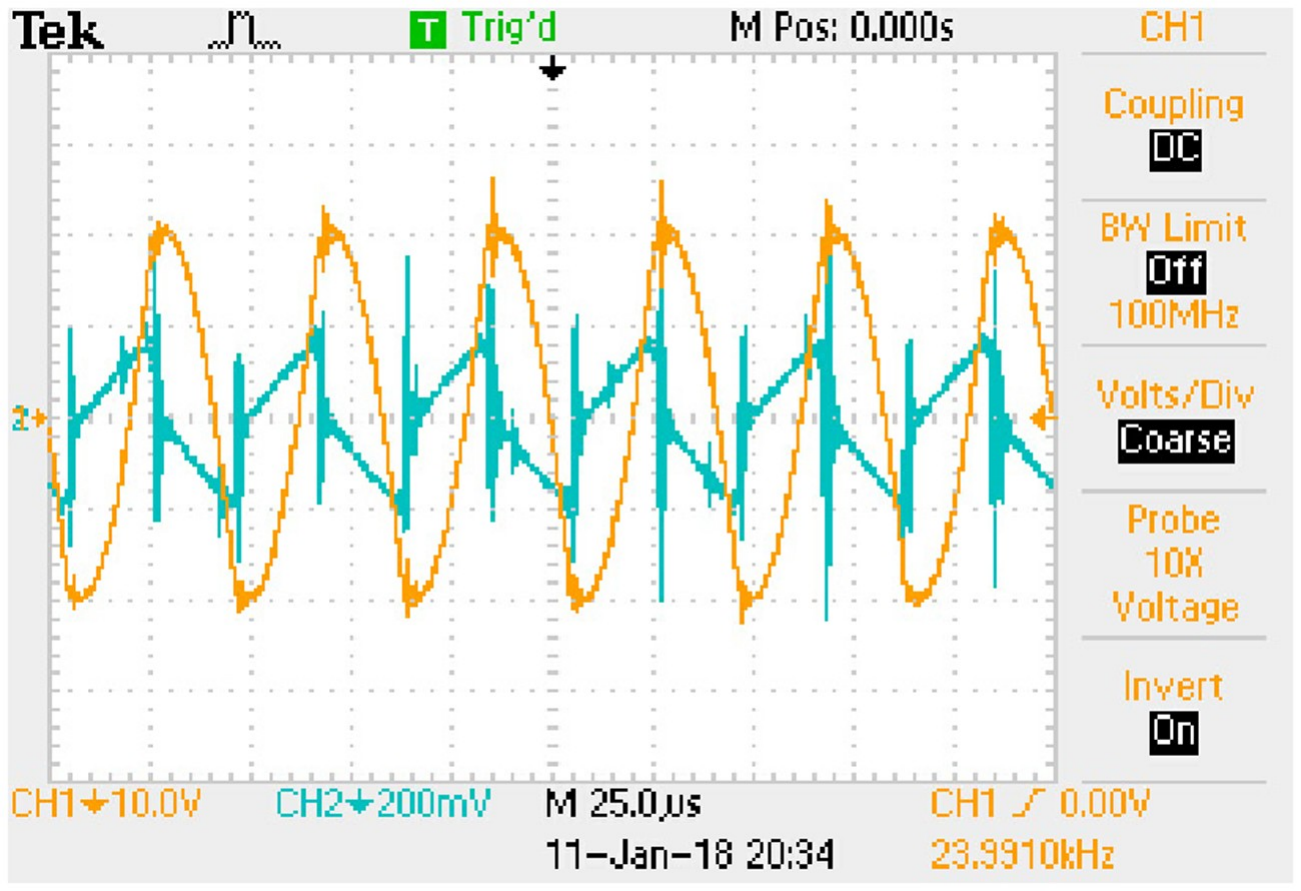
Waveform of key variables: *u*_*Ca*_ (CH1) and *i*_*Lp*_ (CH2). This is the measured signal.

**Fig 24:**
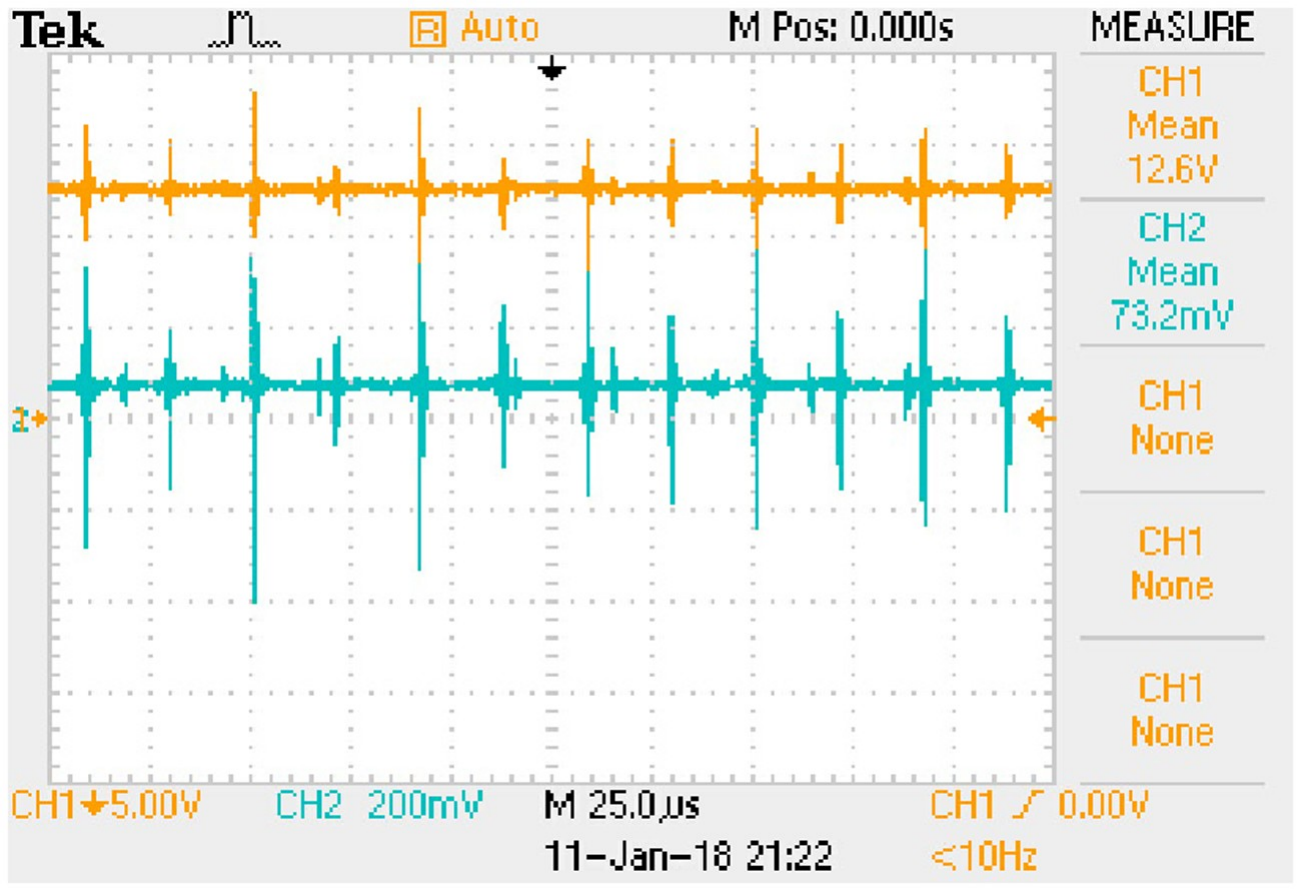
Waveform of key variables: *U*_*C*_ (CH1) and *I*_*Lf*_ (CH2). This is the measured signal.

Under the A4 condition, the *u*_*AB*_ and *i*_*Lp*_ can be seen in Fig 22. The *u*_*Ca*_ and *i*_*Lp*_ can be seen in Fig 23. The *U*_*C*_ and *I*_*Lf*_ can be seen in Fig 24.

The *i*_*Lp*_ is measured by 1Ω sampling resistor and the *I*_*Lf*_ is measured by 0.1Ω sampling resistor.

#### A5

**Fig 25:**
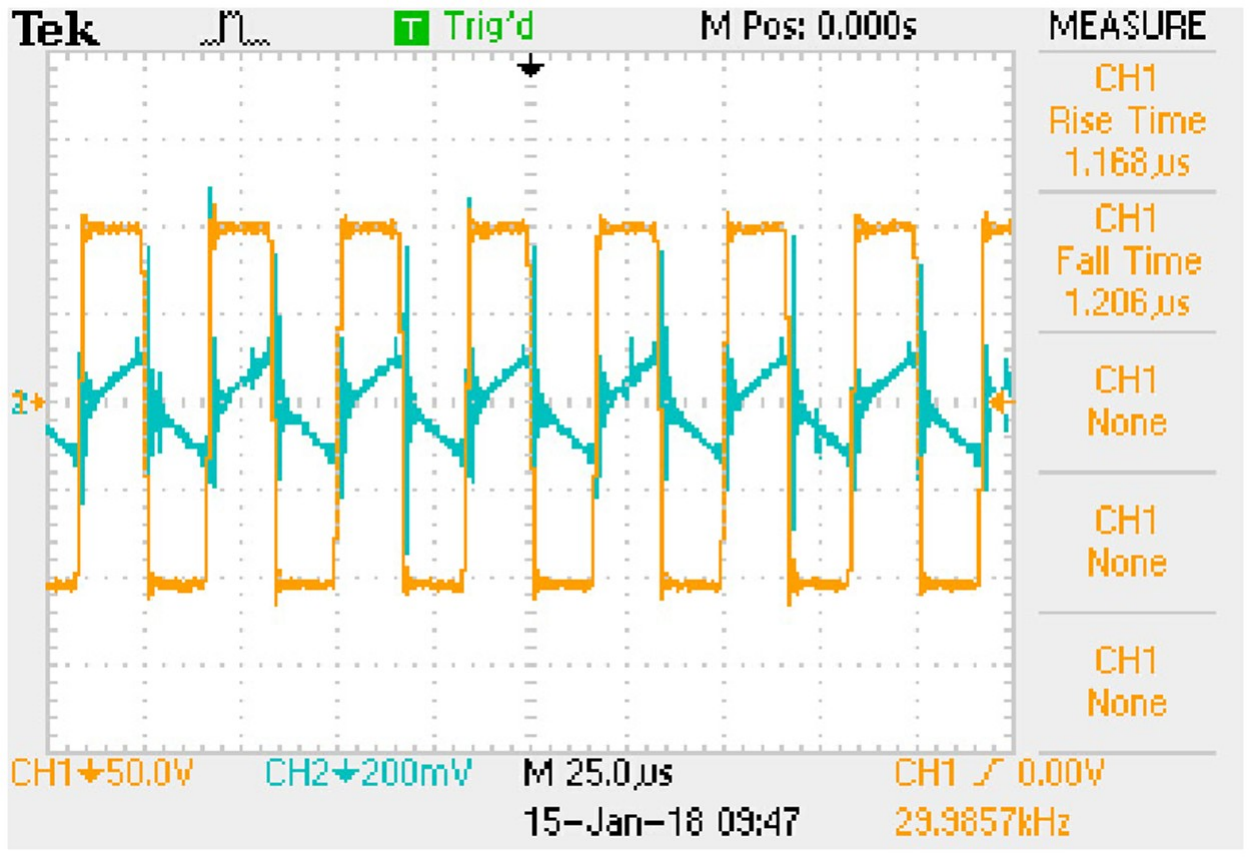
Waveform of key variables: *u*_*AB*_ (CH1) and *i*_*Lp*_ (CH2). This is the measured signal.

**Fig 26:**
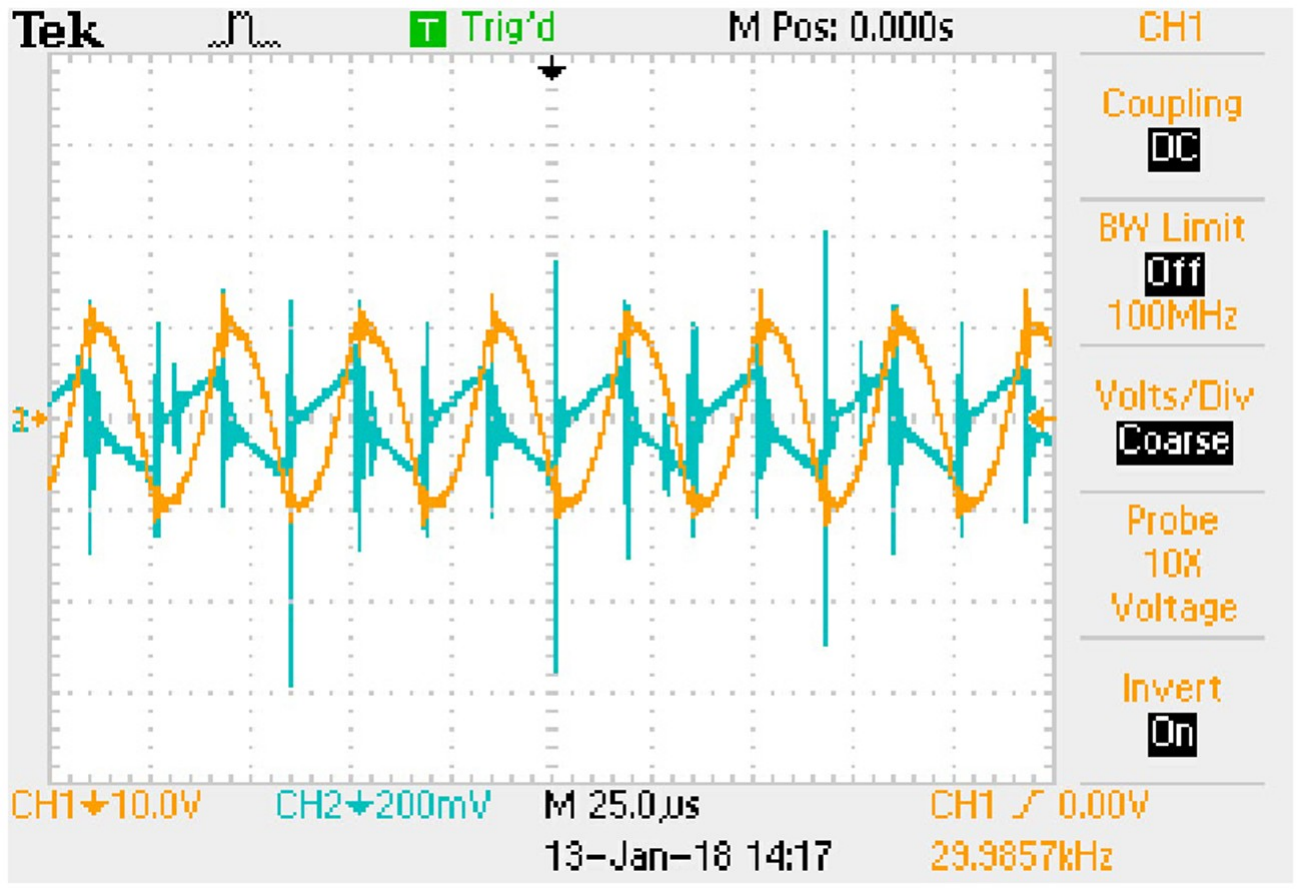
Waveform of key variables: *u*_*Ca*_ (CH1) and *i*_*Lp*_ (CH2). This is the measured signal.

**Fig 27:**
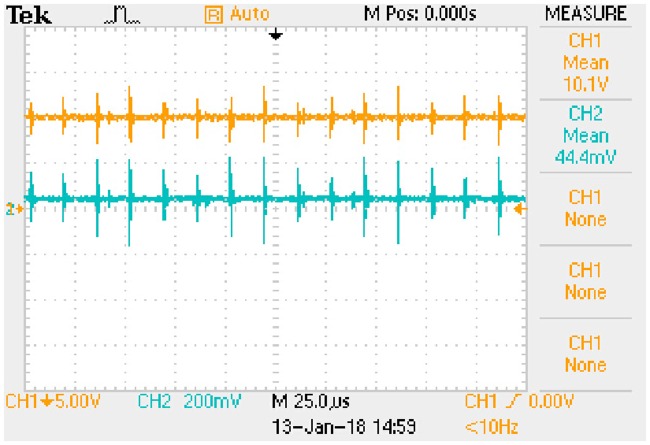
Waveform of key variables: *U*_*C*_ (CH1) and *I*_*Lf*_ (CH2). This is the measured signal.

Under the A5 condition, the *u*_*AB*_ and *i*_*Lp*_ can be seen in Fig 25. The *u*_*Ca*_ and *i*_*Lp*_ can be seen in Fig 26. The *U*_*C*_ and *I*_*Lf*_ can be seen in Fig 27.

The *i*_*Lp*_ is measured by 1Ω sampling resistor and the *I*_*Lf*_ is measured by 0.1Ω sampling resistor.

#### A8

**Fig 28:**
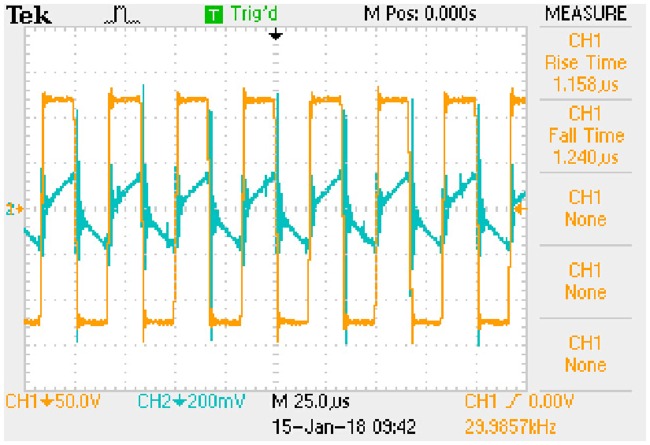
Waveform of key variables: *u*_*AB*_ (CH1) and *i*_*Lp*_ (CH2). This is the measured signal.

**Fig 29:**
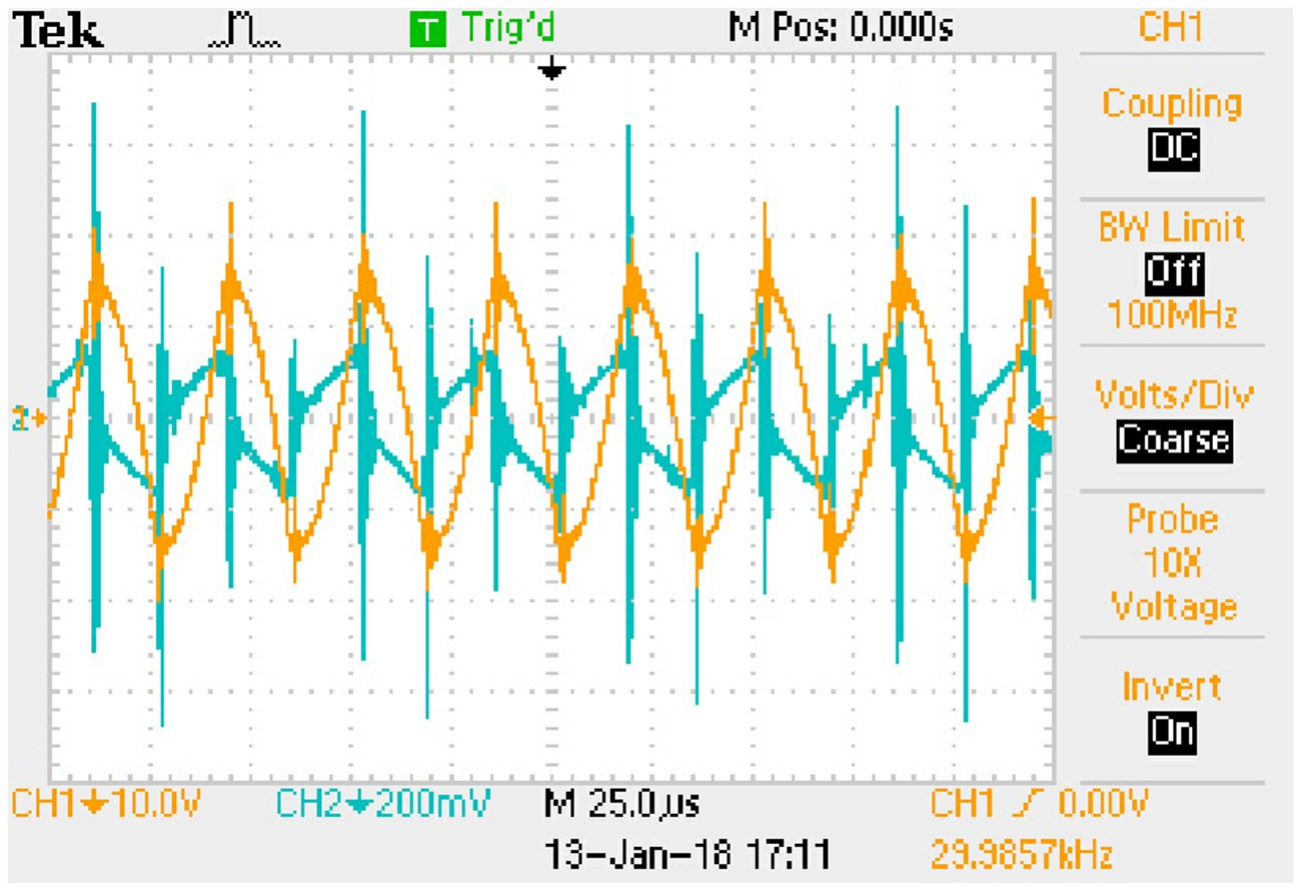
Waveform of key variables: *u*_*Ca*_ (CH1) and *i*_*Lp*_ (CH2). This is the measured signal.

**Fig 30:**
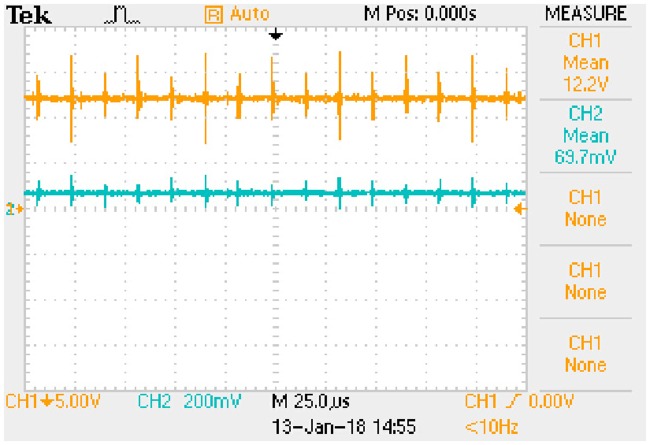
Waveform of key variables: *U*_*C*_ (CH1) and *I*_*Lf*_ (CH2). This is the measured signal.

Under the A8 condition, the *u*_*AB*_ and *i*_*Lp*_ can be seen in Fig 28. The *u*_*Ca*_ and *i*_*Lp*_ can be seen in Fig 29. The *U*_*C*_ and *I*_*Lf*_ can be seen in Fig 30.

The *i*_*Lp*_ is measured by 1Ω sampling resistor and the *I*_*Lf*_ is measured by 0.1Ω sampling resistor.

Based on the storage data in the Tektronix oscilloscope TDS2012B, the actual *t*_*on*_ shown in Fig 3 and key indexes of all the eight working conditions are listed in Tables 20 and 21 respectively.

**Table 20:** The *t*_*on*_ under different working conditions.

States	*t*_*on*_(μs)
A1	19.6068
A2	19.6373
A3	19.5833
A4	19.6028
A5	15.4797
A6	15.4967
A7	15.4447
A8	15.4677

**Table 21:** Key indexes under different working conditions.

States	*U*_*Ca_peak*_(V)	*I*_*Lp_peak*_(A)	*U*_*C_av*_(V)	*I*_*Lf_av*_(A)
A1	14.4	0.128	10.4	0.468
A2	17.6	0.152	12.7	0.559
A3	16.8	0.136	10.3	0.609
A4	20.4	0.168	12.6	0.732
A5	10.4	0.116	10.1	0.444
A6	12.8	0.140	12.2	0.534
A7	12.0	0.130	9.97	0.577
A8	14.8	0.156	12.2	0.697

### 8.2 Results of transient state

**Fig 31:**
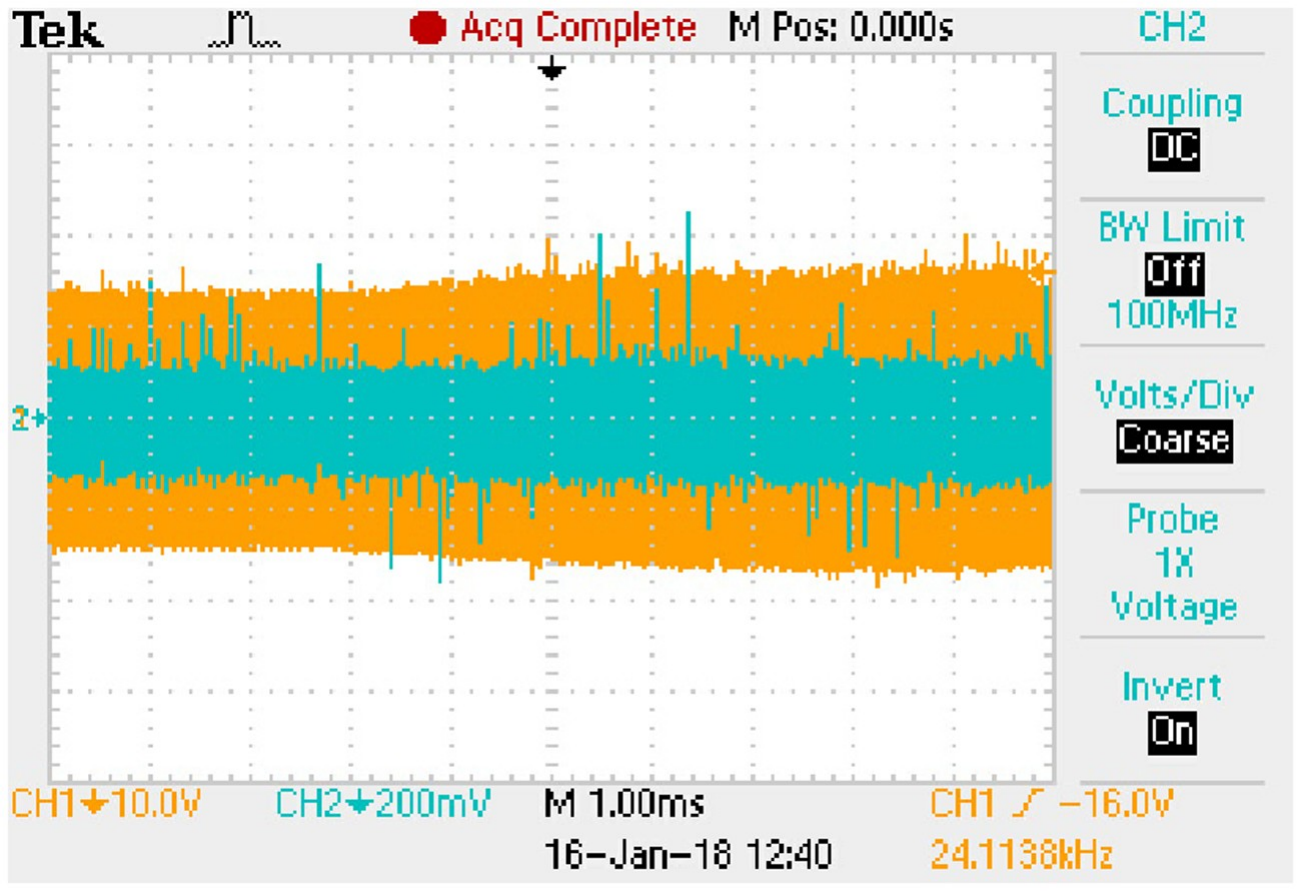
Waveform of *u*_*Ca*_ (CH1) and *i*_*Lp*_ (CH2): State (a). This is the measured signal.

**Fig 32:**
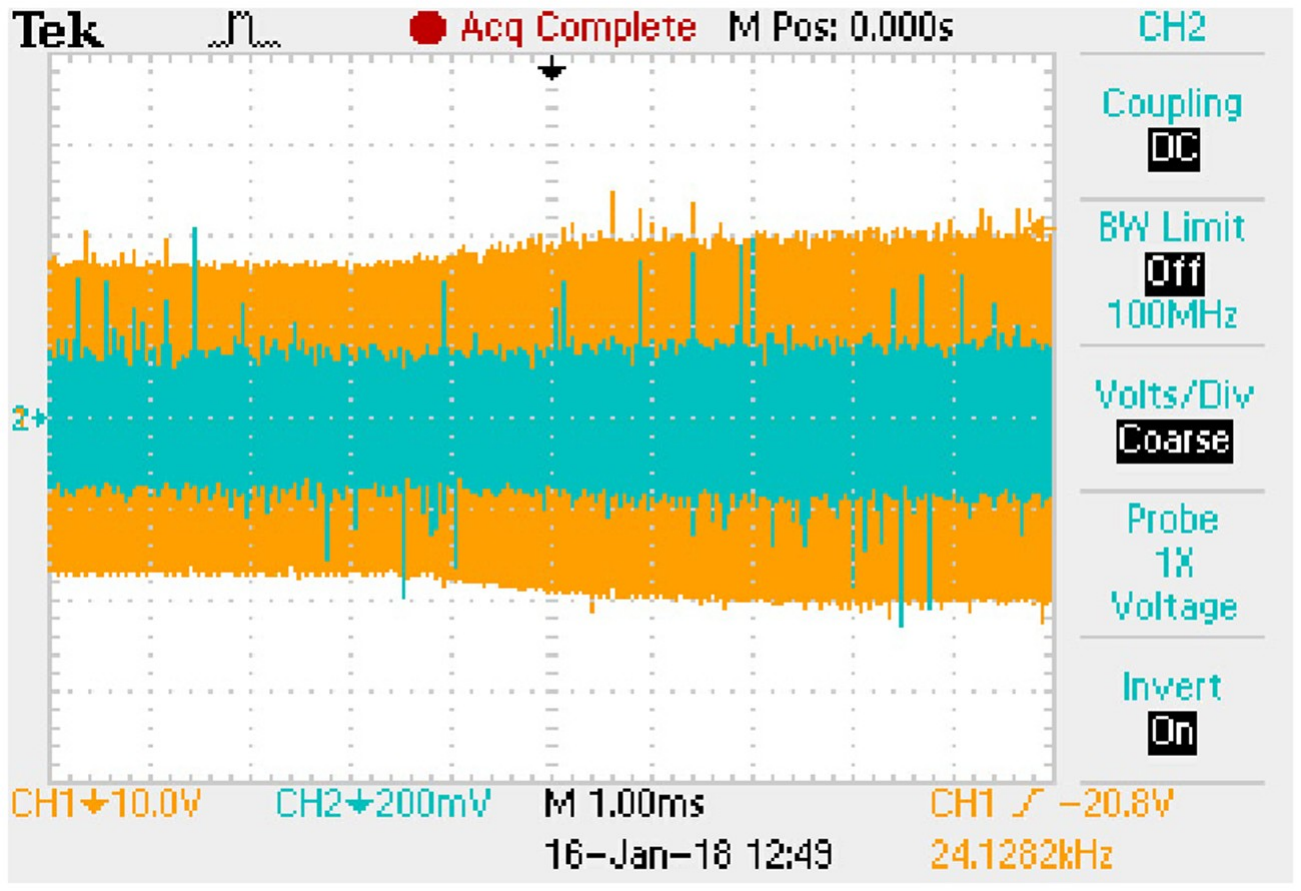
Waveform of *u*_*Ca*_ (CH1) and *i*_*Lp*_ (CH2): State (b). This is the measured signal.

**Fig 33:**
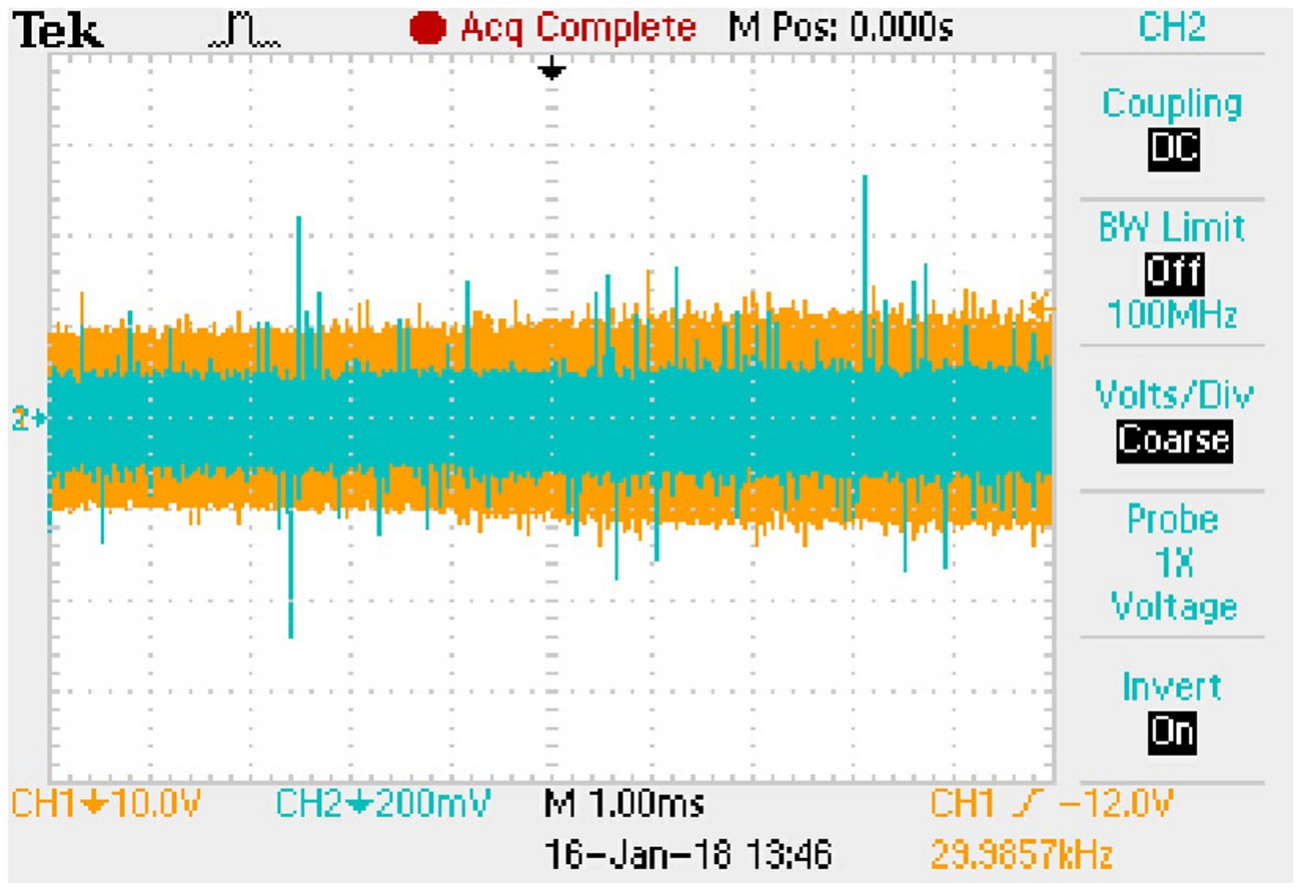
Waveform of *u*_*Ca*_ (CH1) and *i*_*Lp*_ (CH2): State (c). This is the measured signal.

**Fig 34:**
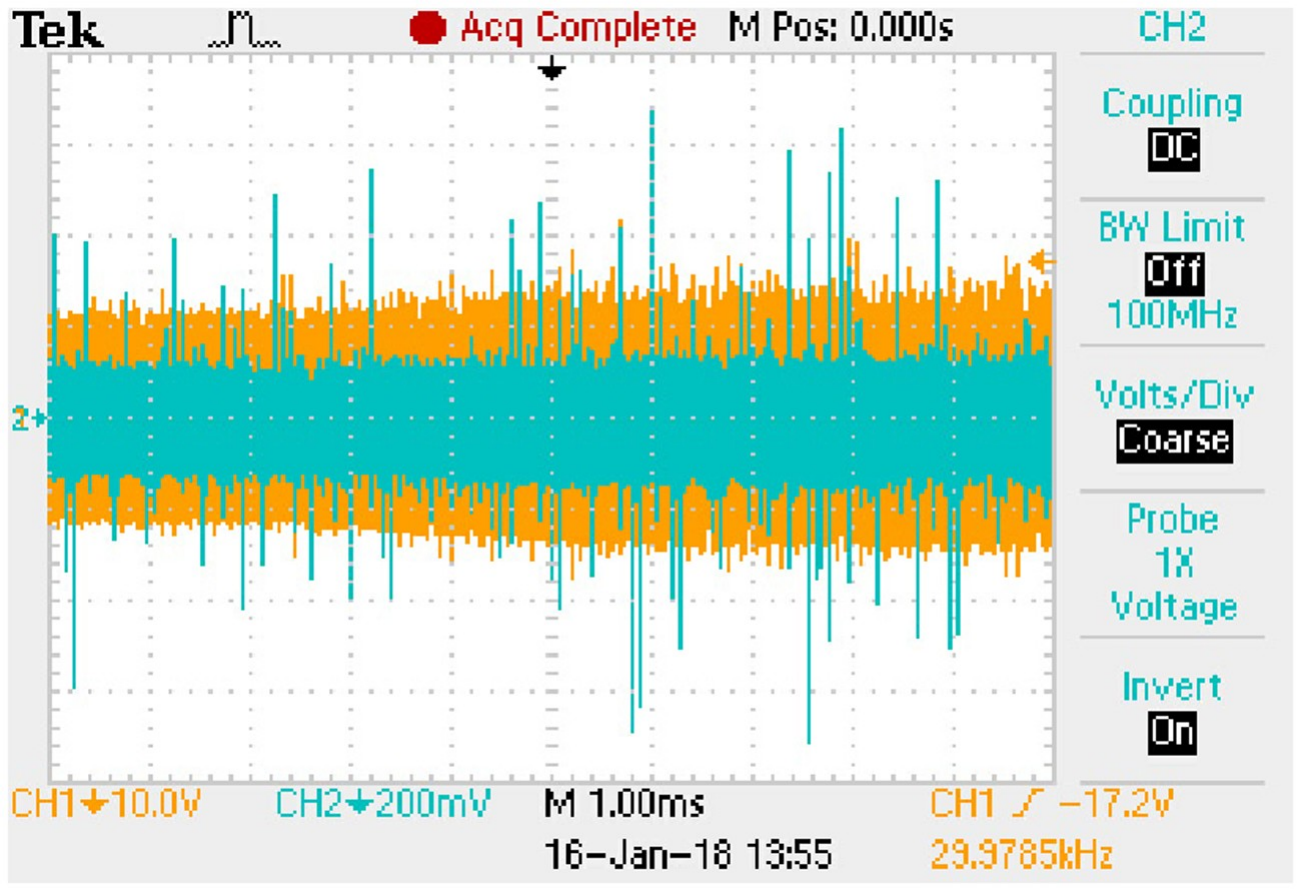
Waveform of *u*_*Ca*_ (CH1) and *i*_*Lp*_ (CH2): State (d). This is the measured signal.

The state (a), state (b), state (c), and state (d) mentioned in the numerical results continue to be used. In order to verify the approximate expressions of *u*_*Ca*_*peak*_ and *i*_*Lp*_*peak*_ obtained in the typical FHA model and proposed model, the actual transient process of *u*_*Ca*_ and *i*_*Lp*_ are given in Figs 31, 32, 33 and 34 respectively.

**Fig 35:**
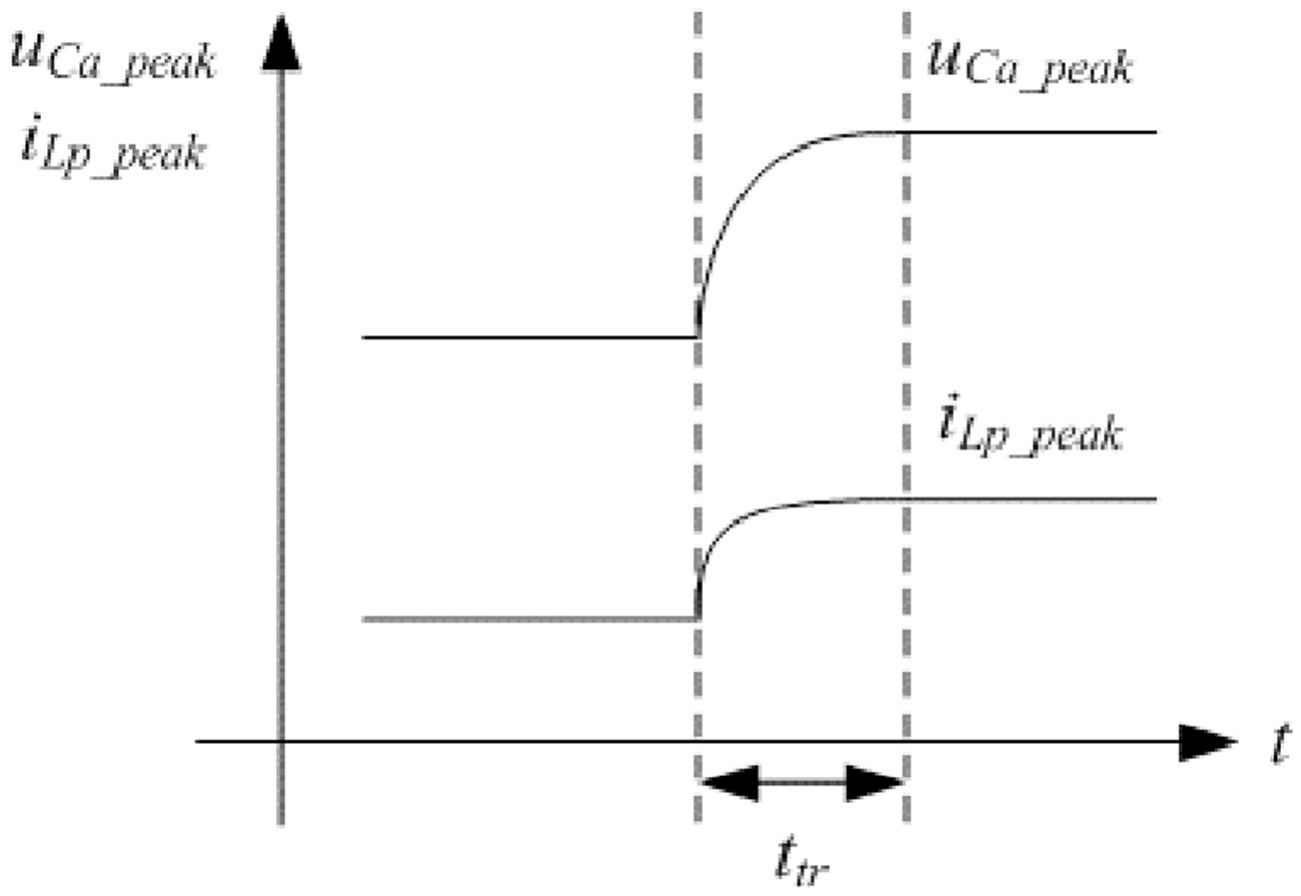
Positive envelopes of *u*_*Ca*_ and *i*_*Lp*_. This is the actual relationship between *u*_*Ca*_*peak*_ and *i*_*Lp*_*peak*_.

Both positive envelopes of *u*_*Ca*_ and *i*_*Lp*_ in Figs 31 to 34 can be illustrated by Fig 35 uniformly. It is these envelopes that represent the *u*_*Ca*_*peak*_ and *i*_*Lp*_*peak*_ respectively.

It can be observed from Figs 31 to 34 and 35 that the transition time of four states is a little greater than 1 milliseconds unanimously. Namely, *t*_*tr*_ in Fig 35 can be taken as 1 milliseconds uniformly under state (a) to state (d). It is known that the Δ*t* in the theoretical analysis is coincident with *t*_*tr*_. The *u*_*Ca*_*peak*_ and *i*_*Lp*_*peak*_ change monotonously and there is no overshoot under the aforementioned four states. In addition, there are two notes as follows.

1. The *u*_*Ca*_*peak*_ and *i*_*Lp*_*peak*_ measured before and after the transient state are different from that only measured under the steady state because range of time axis is relatively large and there are only a few sampling points in every switching period.
2. The *u*_*Ca*_ and *i*_*Lp*_ always satisfy the differential relation in the actual converter. Therefore, the variation tendency of *u*_*Ca*_*peak*_ and *i*_*Lp*_*peak*_ is always coincident in the transient process.

Therefore, the transition time *t*_*tr*_ determines extent of approximation about the indexes under the transient state. The lower fractional errors of *t*_*tr*_ between models and converter mean the higher extent of approximation.

## 9 Discussions

### 9.1 Interpretation of results

1 Observing the key indexes under steady state and transient state in the following indicated tables, equations, and figures, we can draw a conclusion as follows.

**Proposed model**: Table 19, and (72a) to (75b).

**Experiment**: Figs 19 to 30, Table 21, and Figs 31 to 34.

**Conclusion**:

For the proposed model, fractional errors of key variables are listed as follows.

1. Fractional errors of *U*_*Ca_peak*_ are 0.62%, 3.44%, 8.17%, 9.96%, 4.15%, 0.66%, 2.47%, and 5.80% respectively under A1 to A8.
2. Fractional errors of *I*_*Lp_peak*_ are 0.86%, 0.99%, 2.35%, 1.31%, 5.34%, 3.79%, 1.54%, and 0.83% respectively under A1 to A8.
3. Fractional errors of *U*_*C_av*_ are 0.40%, 1.60%, 0.02%, 1.43%, 1.80%, 1.67%, 2.44%, and 0.99% respectively under A1 to A8.
4. Fractional errors of *I*_*Lf_av*_ are 7.78%, 6.85%, 6.06%, 5.74%, 3.51%, 3.22%, 1.66%, and 1.79% respectively under A1 to A8.
5. Fractional errors of transition time are 16.15%, 15.44%, 1.81%, and 1.08% respectively under state (a) to state (d).

Therefore, the maximum fractional errors of *U*_*Ca_peak*_, *I*_*Lp_peak*_, *U*_*C_av*_, and *I*_*Lf_av*_ are 9.96%, 5.34%, 2.44%, and 7.78% respectively under the eight working conditions. In addition, the maximum fractional error of transition time is 16.15% under the state (a) to state (d). Differences of key indexes between proposed model and experiment are acceptable under steady state and transient state. Furthermore, the Table 17 is verified and it shows that the equivalent principle of indexes is reasonable.

2 Observing the *t*_*on*_ in the following indicated tables and figures, we can draw a conclusion as follows.

**Proposed model**: Table 18.

**Experiment**: Figs 19, 22, 25, 28 and Table 20.

**Conclusion**:

For the proposed model, fractional errors of *t*_*on*_ are 2.69%, 2.62%, 2.74%, 2.73%, 2.38%, 2.41%, 2.49%, and 2.48% under A1 to A8 respectively.

Therefore, the maximum fractional error of *t*_*on*_ is 2.74% in the eight working conditions. Fig 3 is verified in accordance with aforementioned figures and the differences of *t*_*on*_ between proposed model and experiment are acceptable. Furthermore, theoretical analyses on the deficiencies of FHA can be supported by the fact.

3 Observing the peak values of *u*_*Ca*_ and *i*_*Lp*_ under the steady state in the following indicated tables and figures, we can draw a conclusion as follows.

**Typical FHA model**: Table 16.

**Proposed model**: Table 19

**Experiment**: Figs 20, 23, 26, 29 and Table 21.

**Conclusion**:

For the typical FHA model, fractional errors of *U*_*Ca_peak*_ and *I*_*Lp_peak*_ are listed as follows. On the other hand, fractional errors in the proposed model have been given in the 1.

1. Fractional errors of *U*_*Ca_peak*_ are 15.50%, 17.08%, 12.93%, 13.95%, 16.21%, 18.27%, 10.16%, and 12.61% respectively under A1 to A8.
2. Fractional errors of *I*_*Lp_peak*_ are 14.22%, 13.36%, 2.94%, 5.71%, 15.26%, 15.71%, 6.46%, and 6.47% respectively under A1 to A8.

Therefore, the average fractional errors of *U*_*Ca_peak*_ and *I*_*Lp_peak*_ in the typical FHA model are 14.59% and 10.02% respectively under the eight working conditions. Similarly, the corresponding average fractional errors in the proposed model are 4.41% and 2.13% respectively. In other words, the differences of aforementioned tables between proposed model and experiment are less than that between typical FHA model and experiment.

4 Observing the average values of *u*_*C*_ and *i*_*Lf*_ under the steady state in the following indicated tables and figures, we can draw a conclusion as follows.

**Typical FHA model**: Table 16.

**Proposed model**: Table 19

**Experiment**: Figs 21, 24, 27, 30 and Table 21.

**Conclusion**:

For the typical FHA model, fractional errors of *U*_*C_av*_ and *I*_*Lf_av*_ are listed as follows. On the other hand, fractional errors in the proposed model have been given in the 1.

1. Fractional errors of *U*_*C_av*_ are 10.25%, 8.34%, 11.25%, 9.13%, 10.45%, 9.73%, 11.89%, and 9.73% respectively under A1 to A8.
2. Fractional errors of *I*_*Lf_av*_ are 2.07%, 2.56%, 4.53%, 4.36%, 4.68%, 4.46%, 7.42%, and 6.70% respectively under A1 to A8.

Therefore, the average fractional errors of *U*_*C_av*_ and *I*_*Lf_av*_ in the typical FHA model are 10.10% and 4.60% respectively under the eight working conditions. Similarly, the corresponding average fractional errors in the proposed model are 1.29% and 4.58% respectively. In other words, the differences of aforementioned tables between proposed model and experiment are less than that between typical FHA model and experiment.

5 Observing the peak values of *u*_*Ca*_ and *i*_*Lp*_ under the transient state in the following indicated equations and figures, we can draw a conclusion as follows.

**Typical FHA model**: (65a) to (68b).

**Proposed model**: (72a) to (75b).

**Experiment**: Figs 31 to 34.

**Conclusion**:

For the typical FHA model, fractional errors of transition time are nearly the same. On the other hand, fractional errors in the proposed model have been given in the 1.

Therefore, the average fractional error of transition time in the typical FHA model is 16.78% under the state (a) to state (d). Similarly, the corresponding average fractional error in the proposed model is 8.62%. Namely, the differences of aforementioned equations and corresponding figures between proposed model and experiment are less than that between typical FHA model and experiment.

### 9.2 Advantages of proposed model

Based on the Table 2 and aforementioned interpretation of results, advantages of proposed model can be listed as follows.

1. For models in the existing literatures and this paper, complexity of both typical FHA model and proposed model is lowest.
  There are five same basic state variables in the typical FHA model and proposed model. They are *u*_*Ca*_, *i*_*Lp*_, *i*_*Lm*_, *I*_*Lf*_, and *U*_*C*_ respectively. So the dimension of state variables is five. On the other hand, the variable *ω* in the typical FHA model and proposed model is uniform function of time. So the number of internal functions related to *ω* is one. According to the two points, complexity of both typical FHA model and proposed model is five and it is lower than the Table 2.
2. Precision of proposed model is higher than that of typical FHA model when converter works under the steady state and transient state.
  Precision of models can be verified by the fractional errors of key indexes under the steady state and the fractional errors of transition time in the transient process. When converter works under the steady state, fractional errors of key indexes in the proposed model are generally lower than that in the typical FHA model. In addition, the average fractional errors of key indexes in the proposed model are all lower than that in the typical FHA model. When converter works under the transient state, fractional errors of transition time in the proposed model are all lower than that in the typical FHA model. So the average fractional error of transition time in the proposed model is lower than that in the typical FHA model. Therefore, it can be concluded that the precision of proposed model is higher than that of typical FHA model.
3. Applicability of proposed model is better than that of typical FHA model when resonant variables are not standard sinusoidal signals.
  There are a number of harmonics exist in the resonant variables under studied CCM condition. It has been fully considered in the improved analyses which are the foundation of proposed model. Maximum fractional errors of key indexes, *t*_*on*_ under the steady state, and maximum fractional error of transition time are all acceptable and reasonable. On the other hand, precision of the two models and deficiencies of FHA are considered. It can be draw a conclusion that proposed model is more applicable than typical FHA model when nonstandard sinusoidal signals occur in the LLC network.

In summary, complexity of proposed model are the same that of typical FHA model but precision of proposed model is higher than that of typical FHA model when current of filter inductor works under CCM situation. Facing with the condition, proposed model is more applicable than typical FHA model.

### 9.3 Summarization of observations

According to the whole theoretical analyses, numerical results, and experimental results, key observations of typical FHA model and proposed model can be summarized in terms of complexity, precision, and effectiveness respectively.

#### 1 Complexity

1. Complexity of models can be defined as the size of each table in the DSP.
  Given the application of models and the universality of look-up table method, complexity of models is defined according to the size of each table which means state variables at next moment under the certain state variables, input voltage, and load at present moment. Therefore, complexity of models is determined by the dimension of state variables and the number of internal functions related to switching angular frequency.
2. Peak values of resonant state variables and average values of slow state variables can be taken as indexes between models and actual converter.
  For the LLC converter, peak values of the three resonant state variables and average values of the two slow state variables are basic elements which can reflect properties of converter. In addition, they can be taken as the direct references in further design. Therefore, they are the indexes which are used to measure different models and actual converter.

#### 2 Precision

1. Deficiencies of typical FHA can be explained by harmonic generation mechanism of primary current and influence of Fourier series on the equivalent circuit.
  Typical FHA analyses are based on the well sinusoidal selectivity of LLC network. When the current of filter inductor works under CCM, resonant variables are distorted because the phenomenon results from the transient process of MOSFETs and rectifier diodes. Therefore, insufficiencies of typical FHA can be analyzed on the harmonic generation mechanism of primary current and influence of Fourier series on the equivalent circuit.
2. For the proposed model, it is important to take fully use of the improved analyses on the steady state and transient state.
  Proposed model is originated from the improved analyses on the converter. It is similar to the typical FHA analyses that the steady state and transient state are further analyzed in the improved method. The transient process of MOSFETs and related fast recovery diodes, transient process of Schottky rectifier diodes, piecewise equivalent circuit, appropriate selection of variables, and simplified modified dynamic equations are all considered in the improved analyses. It is these considerations that lay the foundation for proposed model.

#### 3 Effectiveness

It is an effective way that the equivalent principle of indexes between improved analyses and proposed model is utilized.

Proposed model is established according to the equivalent principle of indexes between improved analyses and proposed model. It can ensure the proposed model has low complexity and high precision. Given the importance and reference value of indexes under the steady state and transient state, it is appropriate that improved analyses and proposed model is equivalent in meaning of indexes.

In summary, the improved analyses and the equivalent principle of indexes are the core of proposed model when converter works under CCM. For the proposed model, complexity and precision are guaranteed by the core.

## 10 Conclusion

This paper proposes an improved FHA model based on the improved analyses and the equivalent principle of indexes in the certain work region so that large signal model has low complexity and high precision when the current of filter inductor works under CCM. Firstly, complexity of models is defined as the size of each table in the DSP. Peak values of resonant state variables and average values of slow state variables are regarded as the indexes between models and actual converter. Complexity of models based on the FHA is lowest in the existing literatures and this paper. Secondly, equivalent circuit based on the typical FHA is derived. Steady state and transient state are briefly analyzed. Therefore, corresponding large signal model called typical FHA model is obtained. Thirdly, insufficiency of typical FHA under this CCM condition is formulated in detail. The two main points are harmonic generation mechanism of primary current and influence of Fourier series on the equivalent circuit. Meanwhile, the work region is given. Furthermore, transient process of switches at the arms of full-bridge, transient process of Schottky rectifier diodes, and piecewise equivalent circuit under the steady state are taken into account. Appropriate selection of variables and simplified modified dynamic equations under the transient state are also considered. Equivalent circuit and improved FHA model based on the equivalent principle of indexes are obtained. Corresponding large signal model is called proposed model. Lastly, numerical results and experimental results are achieved. Rationality of analyses on the proposed model can be verified by comparing numerical results of proposed model with related experimental results. Complexity of proposed model is the same as that of typical FHA model. Meanwhile, it can be further generalized that proposed model is closer to actual converter in meaning of indexes by comparing the differences between typical FHA model, proposed model, and actual converter.

## 11 Appendix

### 1 Solving process of (11)

In this paper, the derivative variables in (11) represent the change rate of *I*_*Lf*_ and *U*_*C*(*eq*)_ from old steady state to new steady state when the converter happens to step change. Under this situation, *I*_*Lf*_ and *U*_*C*(*eq*)_ vary monotonously because of the over-critical damping peculiarity in the LCR network composed of filter inductor L_f_, filter capacitor C, and equivalent load.

*I*_*Lf*_ and *U*_*C*(*eq*)_ are described as *I*_*Lf*_o_ and *U*_*C*(*eq*)_o_ under the old steady state respectively. Correspondingly, they are described as *I*_*Lf*_n_ and *U*_*C*(*eq*)_n_ under the new steady state respectively. All the values are known by the means of steady-state analysis. The time interval between *t*_0_ and *t*_1_ in (11) is described as Δ*t*. Derivative variables in (11) can be estimated according to the following relationships:

$$R_{o}=\frac{8}{\pi^{2}}R$$

$$\frac{I_{Lf\_n}+I_{Lf\_o}}{2}=C\frac{U_{C(eq)\_n}-U_{C(eq)\_o}}{\Delta t}+\frac{U_{C(eq)\_n}+U_{C(eq)\_o}}{2R_{o}}$$

$$\left|{\dot{I}}_{Lf}\right|=\frac{\left|I_{Lf\_n}-I_{Lf\_o}\right|}{\Delta t}$$

$$\left|{\dot{U}}_{C(eq)}\right|=\frac{\left|U_{C(eq)\_n}-U_{C(eq)\_o}\right|}{\Delta t}.$$

Furthermore, *U*_*C*(*eq*)_ is expressed approximately in the following function under the transient state:

$$U_{C(eq)}(t)=\frac{U_{C(eq)\_n}-U_{C(eq)\_o}}{\Delta t}(t-t_{0})+U_{C(eq)\_o}.$$ (76)

From what has been analyzed above, average value of R_eq(FHA)_ in (11) can be obtained because the Δ*t*, derivative variables, and expression of *U*_*C*(*eq*)_ are all known by the approximate analysis.

### 2 Solving process of typical FHA model

In fact, it is the reverse process of steady state analysis and transient state analysis. Solutions under steady state and approximate solutions under transient state are obtained respectively according to the following description.

#### A Solving process under the steady state

1. First-order derivatives of *I*_*Lf*_ and *U*_*C*(*eq*)_ are equal to zero. Namely, it is expressed as follows:
  $${\dot{I}}_{Lf}=0;\;{\dot{U}}_{C(eq)}=0.$$
2. The R_eq(FHA)_ in Fig 2 is solved according to (13d) and (13e). Namely, it is formulated as follows:
  $$R_{eq(FHA)}=\frac{\frac{2}{T}\int_{t}^{t+\frac{T}{2}}\left|L_{M}{\dot{i}}_{Lm}\right|d\tau }{\frac{I_{Lf}}{N}}=N^{2}[\frac{U_{C(eq)}}{\frac{U_{C(eq)}}{\frac{8}{\pi^{2}}R}}]=\frac{8}{\pi^{2}}RN^{2}.$$
3. State variables *u*_*Ca*_, *i*_*Lp*_, and *i*_*Lm*_ in (13a) to (13c) are calculated in the light of following equations:
  $$\{\begin{array}{ll} {\dot{u}}_{Ca} & =\frac{1}{C_{a}}i_{Lp}\\ {\dot{i}}_{Lp} & =\frac{1}{L_{P}}\left[-R_{eq(FHA)}i_{Lp}+R_{eq(FHA)}i_{Lm}-u_{Ca}+\frac{4}{\pi }U_{in}\;\text{sin}(2\pi ft)\right]\\ {\dot{i}}_{Lm} & =\frac{R_{eq(FHA)}}{L_{M}}\left(i_{Lp}-i_{Lm}\right) \end{array}.$$
4. The solved *i*_*Lp*_ and *i*_*Lm*_ are substituted into (13c). Then the *I*_*Lf*_ is solved.
5. The solved *i*_*Lm*_ is substituted into (13d). Then the *U*_*C*(*eq*)_ is solved.
6. The solved *U*_*C*(*eq*)_ is substituted into (13f). Then the *U*_*C*_ can be obtained.

#### B Solving process under the transient state

1. The expression of *U*_*C*(*eq*)_ in (76) is taken as the approximate solution of (13a) and (13f).
2. The known *U*_*C*(*eq*)_ is substituted into (13e). Then the *I*_*Lf*_ can be solved.
3. The known *U*_*C*(*eq*)_ is substituted into (13f). Then the *U*_*C*_ can be solved.
4. Average value of R_eq(FHA)_ is obtained according to the aforementioned analyses. The equivalent load shown in Fig 2 is replaced by the average value of R_eq(FHA)_.
5. State variables *u*_*Ca*_, *i*_*Lp*_, and *i*_*Lm*_ in (13a) to (13c) are calculated approximately in the light of following equations:
  $$\{\begin{array}{ll} {\dot{u}}_{Ca} & =\frac{1}{C_{a}}i_{Lp}\\ {\dot{i}}_{Lp} & =\frac{1}{L_{P}}\left[-{\bar{R}}_{eq(FHA)}i_{Lp}+{\bar{R}}_{eq(FHA)}i_{Lm}-u_{Ca}+\frac{4}{\pi }U_{in}\;\text{sin}(2\pi ft)\right]\\ {\dot{i}}_{Lm} & =\frac{{\bar{R}}_{eq(FHA)}}{L_{M}}\left(i_{Lp}-i_{Lm}\right) \end{array}.$$

In summary, the complete solving process of (13a) to (13f) has been generalized. It can be regarded as the applying process of steady state analysis and transient state analysis on the converter.

## Supporting information

S1 File Parameters of actual converter. (PDF)
