# Supplementary material for: An improved large signal model of full-bridge LLC converter
Source: PLoS One. 2018 Oct 19;13(10):e0205904. doi: 10.1371/journal.pone.0205904 (PMC6195276; doi:10.1371/journal.pone.0205904)
Supplement: S1 File — (PDF) [file pone.0205904.s001.pdf]

Parameters of actual converter

Name	Value/Type
MOSFETs Q1~ Q4	IRFP 460
Fast recovery diodes	MUR3060WT
Block capacitor C_a	47nF
[1]AC resistance R_P	1.5149Ω
Leakage inductance L_P	550μH
Excitation inductance L_M	12.2mH
Number N	9
AC resistance R_S	0.0152Ω
Schottky diodes D1 and D2	V50100PW
Filter inductor L_f	250μH
[2]AC resistance R_{Lf}	0.1361Ω
Filter capacitor C	680 μF

1 ESR of C_a and current sampling resistor are 0.42Ω and 1Ω respectively.

2 The current sampling resistor is 0.1Ω.
